# 36th Annual CAPO Conference: Advocating for All: Psychosocial Oncology at the Intersections of Equity, Diversity, and Inclusion, 8–10 June 2021

**DOI:** 10.3390/curroncol28040234

**Published:** 2021-07-15

**Authors:** Peter Traversa

**Affiliations:** Canadian Association of Psychosocial Oncology, Toronto, ON M5A 1S2, Canada; manager@capo.ca

**Keywords:** psychosocial, oncology, cancer, research

## Abstract

On behalf of the Canadian Association of Psychosocial Oncology, we are pleased to present the Abstracts from the 2021 Annual Conference, titled “Advocating for All: Psychosocial Oncology at the Intersections of Equity, Diversity, and Inclusion”. The Conference was held virtually from 8 June 2021 to 10 June 2021. This conference brought together key stakeholders including multidisciplinary professionals from nursing, psychology, psychiatry, social work, spiritual care, nutrition, medicine, rehabilitation medicine, occupational health and radiation therapy for both adult and pediatric populations. Participants included clinicians, researchers, educators in cancer care, community-based organizations and patient representatives. Patients, caregivers and family members presented abstracts that speak to their role in managing cancer experiences and care. Over one hundred (100) abstracts were selected for presentation as symposia, 20-min oral presentations, 10-min oral presentations, 90-min workshops and poster presentations. We congratulate all the presenters on their research work and contribution.

## Abstract Themes

A. Equity, diversity, inclusion, and advocacy in cancer care and/or research
B. Cancer care across the life span (children, adolescents and young adults, adults, and older adults)
C. Complementary and integrative cancer care
D. Community-based and volunteer cancer care services
E. Sociodemographic, culture, and sex/gender issues in cancer
F. Digital health and cancer care
G. Exercise/pre-habilitation and rehabilitation in cancer
H. Implementation science, knowledge translation, and synthesis
I. Survivorship
J. Palliative and end-of-life care
K. Primary, secondary, and tertiary cancer prevention
L. Innovation in psychosocial oncology interventions
M. Oncology clinicians: Workplace issues, multidisciplinary collaboration, and resilience
N. Cancer treatment-related symptom and toxicity management
O. Pandemics and cancer care issues
P. Other value-based and person-centered cancer care

## 

*Symposia*

**S1 Real-World Implementation of Patient-Reported Outcomes (PROs) Programs across Canada: Opportunities, Challenges, Reach, and Innovations**

## 

TungJasmine†Canadian Partnership against Cancer, Toronto, ON, Canada†Moderator.

 

## 1. Summary

Objective: The systematic and standardized screening of patient-reported outcomes (PROs) has been shown to enhance patient–clinician communication, improve symptom management, reduce health service utilization, and even improve survival. Despite this, PRO programs remain scarce outside of clinical trials. In this symposium, the implementation of real-world PRO programs from across Canada will be presented. Presentations will focus on unique opportunities, challenges, reach, and/or innovations. Sample and setting: In Canada, there have been “early adopters” of PRO programs, under various stages of implementation, in different cancer care settings and for various patient populations. Results: In Ontario, the feasibility has been shown for remote symptom monitoring and real-time symptom intensification during systemic treatment. Quebec centers have been working on the implementation of a mobile-based PRO and caregiver-reported outcome (CRO) program, integrating stepped-care clinical pathways. Alberta’s ambulatory oncology model of care integrates a PRO-derived symptom complexity score. Dalhousie University’s Radiation Oncology Department is planning widespread electronic platform installation to facilitate PRO adoption across three Atlantic provinces. Conclusion: Transferrable PRO tools and lessons learned will be shared to inform future PRO program adoption and expansion.

## 

**S1-16 Using Patient-Reported Outcomes (PROs) in Ambulatory Oncology: Utilizing Symptom Complexity to Deliver Targeted, Personalized Care, and Create Clinical Efficiencies**

## 

WatsonLinda^1^,^2^QiSiwei^1^HildebrandApril^1^ChmielewskiLindsi^1^SmithLouise^1^DeIureAndrea^1^WhitworthJames^1^1Alberta Health Services, Calgary, AB, Canada2University of Calgary, Calgary, AB, Canada

 

## 1. Background/Rationale or Objectives/Purpose

Research on symptom complexity among cancer patients is limited, and there is no standardized classification to measure it. In Alberta, the Patient-Reported Outcomes (PRO) project team proposed and developed a symptom complexity algorithm, derived from validated and standardized PRO measures, to classify symptom complexity at an individual level. 

## 2. Methodology or Methods

Starting with a random selection of 520 patients who visited the Tom Baker Cancer Centre (TBCC) from October 2018 to November 2018, a symptom complexity algorithm was developed to classify these patients based on the severity of their symptom scores and their problems/concerns reported. Then, this algorithm was validated with a retrospective chart review with cancer patients who visited a cancer facility in Alberta, Canada from February 2016 to November 2017 (N = 1466).

## 3. Impact on Practice or Results

Based on established validity and clinical relevance, the PRO-derived symptom complexity algorithm was integrated into clinical tools that make the identification of patients who have high or moderate symptom complexity levels easily identifiable. The algorithm acts as a symptom complexity flag, helping healthcare teams identify which patients may need more timely, targeted, and individualized symptom management.

## 4. Discussion or Conclusions

The utility of this type of symptom complexity score is broad. It has been used to identify a personalized dose of care that a patient requires (micro); to identify which clinics may benefit from adjustment to their clinical booking templates or additional staffing (meso), or as a component of program capacity planning (macro). Examples such as these will be shared in this session. 

## 

**S1-21 Remote Symptom Monitoring: A Feasibility Trial of the Mobile Phone-Based Canadian Advanced Symptom Monitoring and Management System (CAN-ASyMS)**

## 

HowellDorisPrincess Margaret Cancer Research Institute, Toronto, ON, Canada

 

## 1. Background/Rationale or Objectives/Purpose

Remote symptom monitoring and real-time symptom management using digital technology can improve symptoms, quality of life, and survival. The Canadian Advanced Symptom Monitoring and Management System (CAN-ASyMS) is one of the most evolved digital technologies for remote daily reporting of PROs followed by back-end risk scoring, automation of self-care advice, and alerting of clinicians for intensification of symptom management support using evidence-based decision support tools using pan Canadian triage guidelines. In this sequential, mixed-method randomized clinical trial, we tested the feasibility and effects of CAN-ASyMS on symptoms, quality of life, healthcare use, and self-efficacy outcomes. In this paper, we present the results of remote monitoring and implications for implementation in “real-world” cancer care. 

## 2. Methodology or Methods

In this sequential, mixed-method randomized clinical trial, we tested feasibility and effects of CAN-ASyMS on symptoms, quality of life, healthcare use, and self-efficacy in adjuvant breast and lymphoma cancer patients receiving systemic therapies. Mixed model regressions for repeated measures and *t*-tests were used to examine effectiveness endpoints. Qualitative interviews were also conducted following the trial and analyzed using content analysis. 

## 3. Impact on Practice or Results

A total of 79 patients completed the study, and we met our feasibility outcomes. Group differences were shown for quality of life, distress, and symptom outcomes. Participants (n = 12) described feeling “safe and secure” and were “reassured” that they were still “connected” to their clinical team. Clinicians described concerns about the number of “alerts” and how these could be managed in current workflow. 

## 4. Discussion or Conclusions

Remote monitoring and real-time symptom management requires moving beyond implementation to service redesign for sustainability. 

## 

**S1-34 Real-World Electronic Implementation of Patient-Reported Outcomes across Cancer Centres in Quebec (e-IMPAQc): Challenges and Successes**

## 

LambertSylvie^1^,^2^FariaRosanna^3^FortinMarie-Andrée^4^KildeaJohn^1^,^5^RosbergerZeev^1^HijalTarek^6^MarchandEvelyne^7^CharpentierDanielle^8^MagalhaesMona^2^1McGill University, Montreal, QC, Canada2St. Mary’s Research Centre, Montreal, QC, Canada3St. Mary’s Hospital Centre, Montreal, QC, Canada4CISSS Laval, Laval, QC, Canada5McGill University Health Centre Research Institute, Montreal, QC, Canada6McGill University Health Centre (MUHC), Montreal, QC, Canada7Hôpital Maisonneuve-Rosemont, Montreal, QC, Canada8Centre Hospitalier de l’Université de Montréal (CHUM), Montreal, QC, Canada

 

## 1. Background/Rationale or Objectives/Purpose

Patient-reported outcome (PRO) programs are complex interventions that require extensive clinical resources and stakeholder buy-in, and they inevitably change clinical and managerial care processes. The purpose of this presentation is to share the lessons learned of a large collaboration among clinicians, managers, patients, and researchers for the implementation of electronic PROs across cancer centers in Quebec (called e-IMPAQc).

## 2. Methodology or Methods

Five adult cancer centers in Quebec have participated in the development of e-IMPAQc over a period of 2 years. Seven working groups were established with representation from each center to ensure the co-design of key aspects of e-IMPAQc (e.g., PRO measures, evaluation, education, technology, and implementation and change management).

## 3. Impact on Practice or Results

Key lessons learned include building electronic PRO screening on knowledge gained from paper-based screening (e.g., using same tools) and, where possible, minimize workflow disruption by completing workflow mapping activities. In turn, this had implications regarding what was included in the program, and determining what is sufficient in real-world PRO screening vs. best evidence. Support from leadership (local and provincial) and leveraging win–win opportunities facilitated buy-in. The most significant challenge was technology, and lessons learned include understanding what is feasible (vs. possible), developing clear deliverables, monitoring budget, being aware of provincial approval needed to facilitate connections, and ensuring participation of IT in the development. With over 49 stakeholders involved, communication was another challenge as well as having a clear governance structure that is maintained.

## 4. Discussion or Conclusions

Findings deliver applicable lessons for development of PRO programs and inform the process of integrating innovations in oncology using real-world evidence.

## 

**S1-52 Electronic Distress Screening and Data Analysis across Dalhousie Cancer Centers in Nova Scotia and New Brunswick (DRO PRO): Multidisciplinary Team Approach**

## 

CaissieAmandaDalhousie Department of Radiation Oncology, Halifax, NS, Canada

 

## 1. Background/Rationale or Objectives/Purpose

Increased uptake of distress screening is challenged by a lack of information technology (IT) resources to allow distress screen scores to be captured efficiently and used effectively, either at point of care or within quality improvement (QI) initiatives. Our project allows for an expansion of electronic symptom screening for point of care use across three Dalhousie Department of Radiation Oncology (DRO) cancer centers (Halifax and Sydney, Nova Scotia, and Saint John, New Brunswick) and large-scale “Big Data” collection and implementation of QI initiatives.

## 2. Methodology or Methods

A multidisciplinary team approach helping identify needs for training, testing, and clinical workflow. 

Three phased rollouts will be planned for the radiation centers spanning a year (2021–2022) and beginning with a chosen center (Halifax) and tumor site (Head and Neck) as pilot. CPQR-endorsed screening tools and existing evidence-based care algorithms will be used to start. The implementation of the data analytics tool will occur in the Halifax center and setup to receive distress screening data transmissions each quarter.

Project measures include uptake of screenings, satisfaction rates of training, identification of referrals made as a result of distress screening, and prevalence of symptom burden.

## 3. Impact on Practice or Results

Introducing electronic distress screening places data collection into the hands of the patient, thereby requiring extensive application training and support for them in addition to the typical staff training needed for new applications. The point of care review of the data builds on current clinical workflow. Data analysis of distress data is to be determined at the committee level.

## 4. Discussion or Conclusions

Engaging multi-disciplinary radiotherapy teams in the planning and messaging of the project are key steps in planning. Building from existing CPQR-endorsed deliverables and utilizing evidence-based algorithms and screening tools allows for focus on the technology rollout planning.

## 

**S2 Transition to Virtual Cancer Care and Psychosocial Support in the Hospital and the Community: Lessons Learned During the COVID-19 Pandemic**

## 

BenderJackie†Cancer Rehabilitation and Survivorship Program, Department of Supportive Care, Princess Margaret Cancer Centre, University Health Network, Toronto, ON, Canada†Moderator.

 

## 1. Summary

There is considerable interest in the potential of virtual solutions to improve the accessibility, patient-centeredness, and cost-effectiveness of cancer care and psychosocial support. Virtual care demonstration projects have shown high levels of satisfaction among patients and providers. Patients value the convenience, time, and cost savings of virtual care, while providers find it useful for reaching a wider patient population and providing patient-centered care. Despite these promising findings, the adoption of virtual care/ support has been slow due to fears that it may be less safe or acceptable, as well as technical, logistical, and regulatory challenges. Furthermore, virtual care may increase healthcare disparities by widening the digital divide.

The transition to virtual care/support in response to the COVID19 pandemic presents an ideal opportunity to examine its acceptability. This symposium will begin with an in-depth look at virtual cancer care usage patterns and patient and provider perspectives at the Princess Margaret Cancer Centre (PM). Next, we will examine the adaptations made to deliver virtual cancer rehabilitation at PM and explore patient and provider experiences. Finally, we will explore client perspectives on virtual community-based support programs at Wellspring. The symposium will conclude with a discussion of priorities for future research and practice.

## 

**S2-30 Virtual Community-Based Support Proves Effective for Those Impacted by Cancer**

## 

BrinkertJudi PerryWellspring Cancer Support Foundation, Toronto, ON, Canada

 

## 1. Background/Rationale or Objectives/Purpose

The Wellspring Cancer Support Foundation provides community-based support to meet the emotional, physical, practical, and informational needs of cancer patients and caregivers. In response to the COVID-19 pandemic, all support programs were adapted for virtual access. Virtual programs included support groups, psycho-educational webinars, individual counseling, and self-paced learning. Wellspring undertook an evaluation to understand barriers to access, uptake, ease of access, satisfaction, and perceived benefit compared to in-person programming. 

## 2. Methodology or Methods

Metrics collected included new memberships, registered program attendance, video views and resource downloads. An electronic survey was distributed to 3722 individuals who had completed memberships between September 2019 and October 2020. Within this group were individuals who participated only in-person, only virtual, or both virtually and in-person.

## 3. Impact on Practice or Results

Between April and October 2020, virtual programs had a total of 27,177 visits, video views, and resource downloads. New memberships were received from 10 provinces/territories. A total of 621 members responded to the survey. Barriers to accessing virtual programs included a focus on other priorities (35%), preference for in-person programs (28%), and lack of awareness (25%). Satisfaction with virtual programs was strong (registered groups—97%, webinars—96%). Respondents accessing virtual programs indicated they were coping better (87%) and feeling less isolated (81%). Most (95%) of respondents found programs easy to access, with 85% expressing minimal technical difficulties, and 87% of respondents indicated a desire for virtual programs post-pandemic.

## 4. Discussion or Conclusions

Overall, virtual program delivery was successful, resulting in a wider audience across Canada, similar outcomes for in-person programming and a confirmed desire for continued virtual delivery post-pandemic.

## 

**S2-24 Delivering Virtual Cancer Rehabilitation Programming during the First 90 Days of the COVID-19 Pandemic: A Multimethod Study**

## 

JonesJenniferLopezChristianChafranskaiaAleksandraEdwardsElizabethChangEugeneLangelierDavidPrincess Margaret Cancer Centre, Toronto, ON, Canada

 

## 1. Background/Rationale or Objectives/Purpose

To describe the adaptations and modifications made to deliver virtual cancer rehabilitation at the onset of the COVID-19 pandemic and understand the experiences of patients and providers adapting to virtual care.

## 2. Methodology or Methods

**Sample and setting:** Patients attending the Cancer Rehabilitation and Survivorship Program (Princess Margaret Cancer Centre Cancer) during the first 90 days of the COVID-19 pandemic.

**Procedure:** Data collection included a framework-driven categorization of modifications made to the program and qualitative interviews with patients and providers. Program data on service delivery of virtual care were compared to the previous 90 days of in-person care. 

## 3. Impact on Practice or Results

A total of 1968 virtual patient visits were completed during the study period. The majority of visits were able to be adapted to virtual delivery. Modifications to the program included format changes, setting changes, and content changes. Virtual care demonstrated a decrease in wait times and an increase in capacity for a variety of visit types. There was an increase/maintenance in the number of completed visits compared to in-person care, with attendance rates ranging from 80 to 93%. Three themes emerged from the interviews and include (1) access to care; (2) meeting support needs; and (3) confidence with assessment and care plan. The interviews revealed that virtual care was an acceptable alternative that provided timely access to care and needed support.

## 4. Discussion or Conclusions

Findings suggest that many appointments can be successfully adapted to virtual formats to deliver cancer rehabilitation programming. We provide several practical recommendations that can be implemented by clinicians and programs to facilitate the adoption and delivery of virtual care.

## 

**S2-67 Patient and Provider Perspectives on Virtual Cancer Care during COVID-19**

## 

BenderJacqueline^1^BabinskiStephanie^1^SamadiOsai^1^LovasMike^2^MelwaniSheena^2^BadzynskiAdam^2^PfaffDamon^2^TroungTran^3^BerlinAlejandro^4^1Cancer Rehabilitation and Survivorship Program, Department of Supportive Care, Princess Margaret Cancer Centre, University Health Network, Toronto, ON, Canada2Smart Cancer Care Program and Healthcare Human Factors, University Health Network, Toronto, ON, Canada3Techna Institute, Univeristy Health Network, Toronto, ON, Canada4Radiation Medicine Program and Smart Cancer Care Program, Princess Margaret Cancer Centre, University Health Network, Toronto, ON, Canada

 

## 1. Background/Rationale or Objectives/Purpose

To gain insight into the acceptability of virtual cancer care (VCC) among patients and providers during the COVID-19 pandemic.

## 2. Methodology or Methods

Two surveys were distributed at the Princess Margaret Cancer Centre. Patients (n = 2627) completed the Your Voice Matters survey adapted for VCC following appointments between August and December 2020, and providers (n = 158) completed a VCC evaluation survey within two months of VCC launch. A total of 171 VCC-related open-ended responses were collected from patients, and 90 were collected from providers. Quantitative responses were analyzed using descriptive statistics, and qualitative open-ended responses were thematically analyzed.

## 3. Impact on Practice or Results

A total of 635 (25%) patients had a virtual visit: 107 (17%) via video and 522 (83%) via phone. The majority of patients (565; 89%) and providers (111; 70%) were satisfied or very satisfied with VCC. Of all patient respondents, 1414 (53.2%) would ask for a virtual visit for their next appointment. Four themes emerged from patients’ open-ended responses: (1) greater accessibility and convenience; (2) scheduling and follow-up communication breakdowns; (3) need for improved functionality; and (4) concerns about care quality and safety. Three themes emerged from providers’ open-ended responses: (1) support for virtual care; (2) need for improved functionality; and (3) concerns about care quality and coordination. 

## 4. Discussion or Conclusions

Patients and providers expressed high levels of satisfaction with VCC, but some raised concerns about technological functionality and the impact on care processes and quality. Recommended improvements include virtualizing care workflows to optimize coordination and efficiency and to enable patients to participate more actively in their care.

## 

**S3 Better Together: Partnering with a Community-Based Support Organization to Understand the Challenges of Men with Testicular Cancer**

## 

BultzBarry^1^,^2^†KellyBrian^3^†1Department of Oncology, Cumming School of Medicine, University of Calgary, Calgary, AB, Canada2Department of Psychosocial Oncology, Tom Baker Cancer Centre, Calgary, AB, Canada3School of Medicine and Public Health, University of Newcastle, Newcastle, NSW, Australia†Moderator.

 

## 1. Summary

Testicular cancer (TC) is most common among adolescent and young adult (AYA) men and has increased in incidence over the past two decades. Further work is needed to understand the psychosocial needs of this young population in order to provide effective psychosocial care.

We will describe the methodology, results, and translational aspects of a qualitative study of AYA TC patients while highlighting a unique collaboration between a TC community support organization (CSO) and the research team.

The symposium will include the following: (1) a description of the qualitative methodology employed to analyze the four focus groups designed to elicit the challenges facing these men in various spheres of their lives (n = 21); (2) a discussion of findings: how the TC experience affects psychosocial dimensions of well-being, including relationships between vulnerability, self-esteem, and body-image; and (3) reflections on the process and impacts of this innovative collaboration shared by a member of the CSO.

This unique international collaboration (from study inception to implications for our community partner) provides a greater understanding of AYA TC patients’ experiences of and views about unmet psychosocial needs including views about care. In addition, it will improve translation of the results into intervention models designed to enhance quality of life.

## 

**S3-83 Exhaustive or Exhausting? Describing the Qualitative Methodology Employed to Analyze Men’s Testicular Cancer Focus Groups**

## 

TavenerMeredith^1^ForbesCaitlin^2^BultzBarry D.^2^,^3^KellyBrian^1^RosbergerZeev^4^SchulteFiona^2^,^5^,^6^1School of Medicine and Public Health, University of Newcastle, Newcastle, Australia2Department of Oncology, Cumming School of Medicine, University of Calgary, Calgary, AB, Canada3Department of Psychosocial Oncology, Tom Baker Cancer Centre, Calgary, AB, Canada4Lady Davis Institute for Medical Research, Jewish General Hospital, Montreal, QC, Canada5Departments of Psychology, Oncology and Psychiatry, McGill University, Montreal, QC, Canada6Hematology, Oncology, Transplant Program, Alberta Children’s Hospital, Calgary, AB, Canada

 

## 1. Background/Rationale or Objectives/Purpose

Focus groups can provide a time-effective way to gather experiential evidence from multiple participants. However, analysis techniques to generate the best evidence vary. The objective of this presentation is to demonstrate how analytical rigor can be achieved. 

## 2. Methodology or Methods

Researchers from four institutions came together to conceptualize, conduct, and analyze focus groups conducted with 21 men aged 25 to 50, who experienced testicular cancer. Four groups were analyzed intensively, coded (1) line-by-line using iterative comparisons of individual and (2) group narratives, with (3) reflexive thematic analysis techniques applied inductively using descriptive coding, then (4) deductive coding was framed by a priori domains of interest, (5) interaction between participants was identified, (6) codes were categorized, and (7) meta-themes were constructed. 

## 3. Impact on Practice or Results

After 14 weeks to recruit participants, 8 h to conduct four focus groups, and 128 h for transcription, the interrogation of 67,918 words of testicular cancer discussion group data took 12 weeks. Two primary meta-themes were constructed, representing “negotiating identity” and “needing to adjust”, under which sat a priori categories including masculinity, vulnerability, sexuality, quality of life, support, relationships, and stress; with additional inductive findings encompassing trauma, loss, time stress, and how we talk about cancer. 

## 4. Discussion or Conclusions

This presentation provides an accessible view of rigorous methods to examine focus group data. The interpretations produced are authentic representations of the men’s testicular cancer experiences, which continue to be explored further with the participants, and key representatives from testicular cancer services to translate findings to clinicians and support organizations. 

## 

**S3-84 Creating an Environment of Support: Understanding the Unique Challenges and Unmet Needs of Young Men Diagnosed with Testicular Cancer**

## 

ForbesCaitlin^1^BultzBarry D.^1^TavenerMeredith^3^RailtonCindy^4^,^5^KellyBrian^3^RosbergerZeev^6^,^7^OberoiDevesh^1^LloydChristopher^8^WhiteBennett^8^SchulteFiona^1^,^9^1Department of Oncology, Cumming School of Medicine, University of Calgary, Calgary, AB, Canada2Department of Psychosocial Oncology, Tom Baker Cancer Centre, Calgary, AB, Canada3School of Medicine and Public Health, University of Newcastle, Newcastle, NSW, Australia4Faculty of Nursing, University of Calgary, Calgary, AB, Canada5CancerControl Alberta, Tom Baker Cancer Centre, Calgary, AB, Canada6Lady Davis Institute for Medical Research, Jewish General Hospital, Montreal, QC, Canada7Departments of Psychology, Oncology, & Psychiatry, McGill University, Montreal, QC, Canada8Oneball Charitable Cancer Organization, Calgary, AB, Canada9Hematology, Oncology, Transplant Program, Alberta Children’s Hospital, Calgary, AB, Canada

 

## 1. Background/Rationale or Objectives/Purpose

Testicular cancer (TC) is the most common cancer affecting adolescent and young adult men. Despite a 5-year survival rate of 96%, patients are faced with immense physical, emotional, and practical challenges. Yet, 95% choose not to complete clinical screening for distress questionnaires. The purpose of this study was to better understand the biopsychosocial needs of patients throughout their TC journey.

## 2. Methodology or Methods

This study was developed in collaboration with Oneball, which is a community organization for TC patients, and an international team of researchers and clinicians. TC patients and survivors were recruited to participate in one of four focus groups exploring the challenges faced throughout the TC journey (i.e., diagnosis, treatment, and survivorship). Focus groups were voice recorded and transcribed. Themes were identified through an iterative deductive and inductive approach. Themes will be finalized through conceptualization with researchers and Oneball members. 

## 3. Impact on Practice or Results

Twenty-one men participated (mean age = 33.5 (SD = 6.2) mean time since diagnosis = 4.2 years (SD = 3.4)). Priorities for biopsychosocial support varied throughout the TC journey. Priorities for biopsychosocial support varied throughout the TC journey. Participants reflected on the value of informational support from clinicians and peers with lived experience when transitioning to life with cancer. A lack of early psychosocial support resulted in a legacy of unmet needs. Interestingly, many participants valued the supportive peer environment within the focus group despite their reluctance to participate in peer support groups. 

## 4. Discussion or Conclusions

This research engaged a community organization and young men with lived experience to explore the support needs of TC patients over time. This work will inform the development of supports tailored to TC patients with an emphasis on peer interactions.

## 

**S3-86 Value Added: Research Partnership with a Community-Based Testicular Cancer Charity**

## 

LloydChristopher^1^StukalinIgor^1^,^2^WhiteBennett^1^BerJeff^1^ForbesCaitlin^3^SchulteFiona^3^,^4^BultzBarry D.^3^,^5^1Oneball—Charitable Cancer Organization, Calgary, AB, Canada2Cumming School of Medicine, University of Calgary, Calgary, AB, Canada3Department of Oncology, Cumming School of Medicine, University of Calgary, Calgary, AB, Canada4Hematology, Oncology, Transplant Program, Alberta Children’s Hospital, Calgary, AB, Canada5Department of Psychosocial Oncology, Tom Baker Cancer Centre, Calgary, AB, Canada

 

## 1. Background/Rationale or Objectives/Purpose

Oneball’s mission is to provide meaningful assistance throughout the testicular cancer (TC) journey and to destroy the stigma around talking about men’s health. Our goal was to integrate patient voices in all stages of a research study designed to understand the needs of young men diagnosed with TC. 

## 2. Methodology or Methods

As survivors of TC and members of Oneball, we were involved in early discussions with researchers to develop the study and research priorities. We piloted the focus group and used our network to recruit participants. We brought our experiences with TC to discussions about the results. We will continue to work together to share the results of this study with our network of survivors.

## 3. Impact on Practice or Results

Bringing our lived experience to early discussions helped guide research priorities including important themes to explore during focus groups. Approximately one-third of participants were recruited through our network. The value gained by this collaboration goes both ways. Oneball members were given a platform to speak candidly and get our voices heard. The relationships developed through this partnership have provided additional avenues for advocacy and will impact our programming.

## 4. Discussion or Conclusions

Oneball believes that cancer is not just a disease, it is a community. We need to give young survivors the tools to navigate their own unique journey far beyond the disease itself. Working together is an important part of achieving this goal. 

## 

**S4 Equity, Diversity, and Inclusion in Pediatric Oncology and Hematology Research and Practice: A Knowledge-to-Action Plan**

## 

PattonMichaela†University of Calgary, Calgary, AB, Canada†Moderator.

 

## 1. Summary

The lack of equity, diversity, and inclusion (EDI) in oncology and hematology research and practice is a serious and complex problem. There is a limited understanding of how diversity influences and shapes the experiences for youths and their families with cancer or hematological conditions. Individuals from different racial, ethnic, and cultural backgrounds often face a disproportionate number of barriers to accessing high-quality care. Due to the overrepresentation of dominant populations in research, findings are typically not generalizable to members of non-dominant groups. The current symposium is guided by a knowledge-to-action framework and seeks to address EDI issues in pediatric oncology research and care. Empirical studies and knowledge translation work are presented. First, a systematic review protocol on the study of cultural influences in pediatric cancer survivors is shared. Second, findings on the effects of perceived racism on social adjustment in children with sickle cell disease is described. Third, the development of a call-to-action proposal aimed to address EDI issues in medical education and practice is outlined. This symposium concludes with a discussion on the implications of existing efforts to advance the field toward greater equity in research and care. Future avenues for EDI endeavors in the field are also explored.

## 

**S4-101 A Systematic Review of Cultural Influences in Pediatric Cancer Survivors: Preliminary Steps**

## 

HouSharonSchulteFionaUniversity of Calgary, Calgary, AB, Canada

 

## 1. Background/Rationale or Objectives/Purpose

(1) To describe the studies that investigate cultural influences (e.g., migration status, acculturation level) in pediatric cancer survivors, including study characteristics (e.g., operationalizations) and current methods used (e.g., study design). (2) To identify the effects and interaction of cultural influence on the psychosocial outcomes of pediatric cancer survivors.

## 2. Methodology or Methods

Database search includes EMBASE, MEDLINE, and PsycINFO. Search terms were developed with the expertise of a Medical Librarian. Articles are included if they met the following criteria: (1) are original research studies; (2) are published in English; (3) include children diagnosed with cancer between 0 and 21 years of age; (4) describe survivors who are at least 5 years from diagnosis and/or 2 years post-treatment; and (5) include an assessment of cultural influences on survivors’ psychosocial outcomes. This study adheres to PRISMA guidelines for completion of systematic reviews and is registered with PROSPERO. Data extraction is based on the Children’s Oncology Group Long-Term Follow-Up Guidelines Late Effects Evidence Table. Extraction also includes analytical approaches, type of cultural variables, and effect measures.

## 3. Impact on Practice or Results

Data extraction is ongoing. Findings will be reported in accordance with PRISMA guidelines and submitted for publication in a peer-reviewed journal. 

## 4. Discussion or Conclusions

Understanding the complex and dynamic process in which culture shapes children and families’ cancer experience is critical to provide optimal care. Knowledge of the experience of childhood cancer survivors from diverse backgrounds can help to alleviate important aspects of their cancer experience, such as their transition from pediatric to adult care, experience of any long-term or late effects of their treatment, and quality of life.

## 

**S4-103 The Impact of Perceived Racism in Children with Sickle Cell Disease and Their Social Adjustment**

## 

ZwickerHaileyForbesCaitlinHouSharonSchulteFionaUniversity of Calgary, Calgary, AB, Canada

 

## 1. Background/Rationale or Objectives/Purpose

Sickle cell disease (SCD) is a severe genetic disorder that impacts the health of over 5000 Canadians. Children with SCD predominantly are from African or Middle Eastern descent and historically experience higher rates of racial discrimination during their healthcare experiences, disease trajectories, and in day-to-day life. The aim of this study is to better understand how perceived racism impacts social adjustment (SA) in children with SCD.

## 2. Methodology or Methods

This study aims to recruit 100 youths (8–18 years old) who either have or have not undergone a transplant for SCD, and their parent/caregivers from across Canada. Individuals are being recruited via social media to complete an online study. SA is being measured by the *Patient-Reported Outcomes Measurement Information System*, and perceived racism is being captured by the *Perceived Racism in Children and Youth.* Additionally, non-disease-related factors such as immigration status and familial socioeconomic status (SES) are being collected. The association between non-disease related factors (i.e., perceived racism, immigration status, and SES) and SA in children will be evaluated using a linear regression model.

## 3. Impact on Practice or Results

Recruitment is ongoing.

## 4. Discussion or Conclusions

The results from this study may incite future research on SCD to explore how intersectional medicine is necessary to establish better treatment and care options. Understanding how factors beyond disease impact other domains of health allows for modifiable targets to be created. In turn, this knowledge can reduce associated barriers to create holistic and inclusive health management and treatment plans.

## 

**S4-102 Increasing Diversity in Medical Education and Healthcare**

## 

NwarohChideraEbdallaAyaKeriMcNiel-InyaniMohamudMursalUniversity of Calgary, Calgary, AB, Canada

 

## 1. Background/Rationale or Objectives/Purpose

The tragic deaths of George Floyd, Ahmaud Arbery, Breonna Taylor, and Kenneth Ross Jr. in 2020 sparked international public outcry on the treatment of Black, Indigenous, and People of Color (BIPOC). Several Canadian public health sectors have described racism as a public health crisis, acknowledging the ways in which race-based health inequities disproportionally affect BIPOC communities. In the context of healthcare and medical education, racism is one of many obstacles that BIPOC students face. In turn, there is a decreased representation of BIPOC individuals in academic and healthcare positions of leadership, which holds implications for the delivery of inclusive and equitable care for BIPOC individuals.

## 2. Methodology or Methods

An evidence-based call-to-action plan was developed by Black medical students at the Cumming School of Medicine from University of Calgary, in collaboration with faculty advisors. This proposal aimed to address issues of inequality in medical education. A needs assessment was conducted to examine the gaps in training for BIPOC medical students. Tools to improve representation, such as community initiatives, were proposed. Wellness resources to meet the unique challenges racialized students face were also outlined. 

## 3. Impact on Practice or Results

These efforts resulted in a number of policy changes at the institutional level as well as informed the anti-racist strategy at the Cumming School of Medicine. Notably, students involved in this proposal received the 2021 Equity, Diversity, and Inclusion Award at the University of Calgary in recognition for their leadership and advocacy work on inclusivity and diversity in medical education.

## 4. Discussion or Conclusions

The increasing representation of BIPOC medical students encourages a greater understanding of and delivery of care to BIPOC communities. In turn, this diversity in medical education can enhance healthcare to vulnerable patients, especially those from BIPOC communities. 

## 

**S5 Innovative Online Interventions in Pediatric, Adolescent, and Young Adults (AYA) Diagnosed with Cancer: A Pediatric/AYA Special Interest Group Symposium**

## 

SchulteFiona†University of Calgary, Calgary, AB, Canada†Moderator.

 

## 1. Summary

Pediatric, adolescents, and young adults (AYA) who have been diagnosed with cancer are at risk of significant physical and psychological symptoms following their diagnosis and treatment. Given the vulnerable stage of development during which these youth receive toxic therapies, young people diagnosed with cancer may experience pain, fatigue, fear of cancer recurrence, and relationship issues. As the survival rates among these populations are high, the number of survivors is increasing dramatically; there are more than 30,000 survivors of childhood cancer, and approximately one in every 530 AYAs aged 20 to 39 years is a cancer survivor in Canada. These individuals will live 50–60 years beyond their diagnosis and treatment. Thus, it is critical that we develop interventions aimed to address their unmet needs.

This symposium will feature innovative online interventions currently being trialed among pediatric and AYA populations. First, we will describe a protocol for a novel web-based pain intervention for survivors of childhood cancer. Next, we will describe virtual movement interventions including physical activity and yoga for pediatric and AYA patients and survivors. Finally, we will discuss a virtual psychosocial group therapy program for AYA cancer patients. The symposium will conclude with a discussion including priorities for future research.

## 

**S5-23 Protocol and Preliminary Results for a Pilot Study of an Online Psychosocial Group Therapy Intervention for Adolescent and Young Adult Cancer Patients**

## 

MoranChelsea^1^BeattieSara^2^KhuMelanie^3^HowlettMelissa^3^,^4^ScheidlJessica^2^SchulteFiona^5^1University of Caglary, Calgary, AB, Canada2Tom Baker Cancer Centre, Calgary, AB, Canada3Alberta Children’s Hospital, Calgary, AB, Canada4Dalhousie University, Halifax, NS, Canada5University of Calgary, Calgary, AB, Canada

 

## 1. Background/Rationale or Objectives/Purpose

Adolescents and young adults (AYA) with cancer have distinct supportive care needs, yet few evidence-based interventions exist. This research aims to evaluate the acceptability, feasibility, and preliminary outcomes of an eight-week psychosocial group intervention for AYAs with cancer.

## 2. Methodology or Methods

Participants are patients currently receiving care from the Alberta Children’s Hospital or Tom Baker Cancer Centre in Calgary, Alberta. Participants must be 15–29 years old, diagnosed with cancer at 15 years old, and within 5 years of end of treatment. The intervention consists of eight weekly 90-min sessions delivered virtually, which were developed based on a literature review and feedback from five AYA patient partners. Each session begins with psychoeducation about a specific topic (e.g., coping with cancer, relationships/dating, body image, fear of cancer recurrence, sexual health), which is followed by a facilitated group discussion. Feasibility is assessed by collecting data on recruitment and retention. Participant satisfaction is used to assess acceptability. Secondary outcomes include quality of life (Functional Assessment of Cancer Therapy-General (FACT-G)), cancer worry, body image, and sexual health.

## 3. Impact on Practice or Results

Nine participants have completed the group to date (16 screened, 11 enrolled, 2 dropouts). All participants indicated that they were satisfied with the service received and would recommend the group to a friend. Post-intervention FACT-G scores increased on average from baseline (baseline mean = 63.90 ± 13.79, post-intervention = 70.38 ± 8.02). Recruitment and data collection are ongoing.

## 4. Discussion or Conclusions

Preliminary results show promise for feasibility, acceptability, and effect of the intervention. The results of this study will be used to refine intervention content, inform future efficacy research, and guide implementation into routine clinical care.

## 

**S5-115 Moving Online to Promote Wellness among Children, Adolescents, and Young Adults Diagnosed with Cancer**

## 

WurzAmandaMcLaughlinEmmaEllisKelseyCulos-ReedS. NicoleUniversity of Calgary, Calgary, AB, Canada

 

## 1. Background/Rationale or Objectives/Purpose

Movement can improve health, functional capacity, and quality of life among young people (i.e., children, adolescents, and young adults) diagnosed with cancer. Programs to promote movement among young people diagnosed with cancer are rare and have been further reduced due to COVID-19 restrictions. We developed two online movement programs: the IMPACT program (one-on-one physical activity for children and adolescents affected by cancer) and the YYA program (group-based yoga for young adults affected by cancer).

## 2. Methodology or Methods

Children and adolescents (5–18 years) with any cancer or blood disease, who are receiving, scheduled to receive treatment, or completed treatment <3 months in Alberta are eligible to participate in the IMPACT program. The IMPACT program offers participants one-on-one, online physical activity sessions 2–3 times/week over 8–12 weeks. Young adults (18–39 years) with any cancer, on- or off-treatment, from across Canada are eligible to participate in the YYA program. The YYA program offers participants group-based, online yoga sessions 1 time/week over 8 weeks. Both programs are collecting measures of wellness and tracking factors impacting implementation.

## 3. Impact on Practice or Results

Findings will be among the first examining the effectiveness and implementation of online movement programs for young people diagnosed with cancer.

## 4. Discussion or Conclusions

The IMPACT and YYA programs represent unique opportunities to offer movement. Beyond the pandemic, online movement programs are a feasible and sustainable way to continue promoting wellness among young people diagnosed with cancer throughout their complex, highly variable treatment and survivorship timelines, when many enter/exit the hospital repeatedly and are from diverse (and remote) areas across Canada.

## 

**S5-113 Internet-Delivered Cognitive Behavioral Treatment for Survivors of Childhood Cancer with Chronic Pain: Proposal for a Pilot Feasibility Study**

## 

PattonMichaelaCarlsonLindaNoelMelanieSchulteFionaUniversity of Calgary, Calgary, AB, Canada

 

## 1. Background/Rationale or Objectives/Purpose

One in four survivors of childhood cancer (SCCs) experiences chronic pain. Youth with chronic pain report increased anxiety, depression, activity limitations, and sleep disturbances. An 8-week web-based cognitive–behavioral treatment for chronic pain (WebMAP) has demonstrated reduction in the burden of pain in youth but has not yet been tested in SCCs. The aims of the current study are to (1) test the feasibility of WebMAP, (2) test WebMAP’s effect on anxiety and depression, activity limitations, and sleep disturbances, and (3) test WebMAP’s effect on parent pain catastrophizing and parental response to their child’s pain. 

## 2. Methodology or Methods

Participants will be 34 SCCs, and one of their parents was recruited from Alberta Children’s Hospital. Inclusion criteria are (1) cancer diagnosis, (2) age 10–17 years, (3) ≥2 years post-treatment or ≥5 years post-diagnosis, and (4) pain present over prior 3 months impairing ≥1 area of daily life and occurring ≥1/month. Participants will complete a pre-treatment questionnaire, including the pain intensity Numerical Rating Scale, Child Activity Limitations Interview, PROMIS-Anxiety, Depression, and Pain Interference, and Adolescent Sleep Wake Scale. Feasibility will be assessed via recruitment and retention rates. Treatment fidelity will be measured by number of modules completed. Upon completion of pre-treatment questionnaires, survivors will begin WebMAP. After 8 weeks, survivors will complete the same questionnaires. We will conduct post-treatment interviews to gather feedback on the program. Participants will complete the same questionnaires 3 months later. 

## 3. Impact on Practice or Results

We expect SCCs will enjoy WebMAP and find it helpful in reducing symptoms of anxiety, depression, activity limitations, and sleep disturbances. 

## 4. Discussion or Conclusions

Demonstrating that WebMAP is useful to SCCs will be an important step in improving pain management in this population.

## 

*Abstract Theme: Equity, Diversity, Inclusion, and Advocacy in Cancer Care and/or Research*

**The Value of the Patient Voice**

## 

BubberVikramBC Patient Safety & Quality Council, Vancouver, Canada. Canadian Partnership against Cancer, Vancouver, BC, Canada

 

## 1. Background/Rationale or Objectives/Purpose

The healthcare system has become a business, and similar to any business, it needs to listen to its customers to succeed. As long as I have been a “customer” of the healthcare system, I have seen a lot from my own eyes. However, there are those working in the system who have not walked the steps I have on my journey and have not seen what I have seen. 

## 2. Impact on Practice or Results

The barrier between the decision makers and the patients needs to be eliminated. The only way is if the patient voice is at the table every time a decision, which will affect their physical or mental care, is made. Every patient voice shares knowledge and experience that is unique to them but often includes feedback from multiple patient voices. You have ears because you are supposed to actively listen and imagine what it would be like to trade places with us and walk in our shoes.

## 3. Discussion or Conclusions

The inherent value of the patient voice and story is priceless. We have evolved from our ancestors who shared stories with each other in times of need and support. We learn from stories, whether it be from highly priced educational institutions or from life experiences. We as patients strive to share our experiences and knowledge not for enrichment but fulfillment. We want to make a difference not for ourselves but for our greater community.

## 

**39 Patient Advocacy in Psychosocial Oncology: Toward a Shared National Agenda**

## 

AttiehSamar^1^ThibodeauKimberley^2^SavasSevtap^3^PagnuttiTeresa^4^LebelSophie^5^MclverChristine^6^GillisHope^7^LaiznerAndréa Maria^8^CrowellPhilip^9^LoiselleCarmen G.^1^,^10^1McGill University, Montreal, QC, Canada2McGill University Health Center, Montreal, QC, Canada3Memorial University, St. John’s, NL, Canada4N/A, Toronto, ON, Canada5University of Ottawa, Ottawa, ON, Canada6Kids Cancer Care Foundation of Alberta, Calgary, AB, Canada7NovaHope, Nova Scotia, Canada8McGill University Health Centre, Montreal, QC, Canada9University of British Columbia, Vancouver, BC, Canada10CIUSSS West-Central, Montreal, QC, Canada

 

## 1. Background/Rationale or Objectives/Purpose

Psychosocial oncology (PSO) advocacy means publicly recommending and defending timely access to comprehensive support for individuals affected by cancer. The CAPO Advocacy Committee (CAC) is currently working toward a shared national advocacy agenda integrating concepts of person-centered approaches and streamlined PSO care pathways. For this purpose, we identified Canadian organizations that advocate for PSO. The CAC now plans to survey these organizations to garner key advocacy priorities. Then, targeted action items will be taken back to provincial cancer agencies and national bodies.

## 2. Methodology or Methods

Initially, 63 Canadian cancer advocacy organizations were identified. Following information exchange and discussions, 46 were retained for their explicit PSO advocacy activities.

To identify PSO advocacy groups, diverse search strategies were used, including searches on Google, social media platforms, and Medline using relevant keywords and MeSH terms. Multidisciplinary members and patient representatives (N = 12) on our CAPO Advocacy committee reviewed the initial list of advocacy organizations for relevance and proactive PSO advocacy initiatives.

## 3. Impact on Practice or Results

Of the initial 63, 46 organizations were found to primarily focus on enhancing types of and access to PSO support, with 26 being Ontario-based, 11 in Quebec, 4 in Alberta, 2 in Newfoundland and Labrador, 2 in British Columbia, and 1 in Manitoba.

## 4. Discussion or Conclusions

Given the significant number of Canadian PSO advocacy organizations identified, the CAC is now working toward consolidating a priority advocacy agenda with specific action items. The CAC would subsequently seek the endorsement of these action items from key stakeholders including clinical, community, and health and social service decision makers. 

## 

**50 The Role of Indigenous Cancer Patient Navigation in Addressing Psychosocial Aspects of Care to Reduce Cancer Burden in Alberta Indigenous Populations**

## 

SmokeArrow Big^1^Petricone-WestwoodDanielle^1^LetendreAngeline^2^1Department of Psychosocial and Rehabilitation Oncology, Tom Baker Cancer Centre, Cancer Care Alberta, Alberta Health Services, Calgary, AB, Canada2Indigenous Communities, Alberta Cancer Prevention Legacy Fund, Alberta Health Services, Calgary, AB, Canada

 

## 1. Background/Rationale or Objectives/Purpose

The purpose of this presentation is to discuss strategies to address psychosocial aspects of cancer care for Indigenous people; leading to reduced cancer burden amongst these populations.

Indigenous people face barriers on entering the cancer pathway related to finances, geography, and a lack of culturally safe care, including racism. This presentation will introduce practical efforts—both Western and Indigenous—that have been made to improve psychosocial care for/with Indigenous people in Alberta. 

## 2. Objectives

1. Identify barriers to psychosocial aspects of care across the cancer continuum,
2. Discuss the role of Indigenous Cancer Patient Navigation (CPN) in reducing cancer burden in Indigenous populations,
3. Demonstrate how professional practice grounded in Indigenous knowledge systems and philosophies of care can improve cancer outcomes for Indigenous people.

## 3. Methodology or Methods

Indigenous people suffer inequities, resulting in added complexities to meeting their needs and providing the best care. This contributes to extra efforts required by Indigenous CPNs to build relationships across multiple health systems, understand the resources available to different Indigenous groups, have knowledge of different Indigenous cultures/practices, and the skills to support and raise awareness amongst non-Indigenous members of the care team. 

## 4. Impact on Practice or Results

Indigenous practitioner-led efforts to ground their practice in Indigenous ways of being, doing, and understanding improve cancer care experiences for Indigenous people, alleviate the burden of work placed on family caregivers, and build confidence and capacity amongst non-Indigenous care professionals. 

## 5. Discussion or Conclusions

Research examples with Alberta Indigenous people will be presented to provide evidence of integrating experiential knowledge into Indigenous professional practice.

## 

**51 Acceptability, Availability, Awareness, Accessibility, and Affordability: Reflections and Recommendations to Promote Equitable Access to Complementary Therapies, Humanitarian Aid, and Other Supportive Care Programs in a COVIDian World**

## 

LeeVirginia^1^,^2^MouchantafRola^1^,^2^BurrSabrina^1^,^2^D’AddarioJoseph^1^,^2^DoumanianRita^1^,^2^1McGill University Health Centre, Montreal, QC, Canada2Cedars CanSupport, Montreal, QC, Canada

 

## 1. Background/Rationale or Objectives/Purpose

Under extraordinary pandemic circumstances, Cedars CanSupport, a charitable organization located in the Cedars Cancer Centre of the McGill University Health Centre, successfully adapted its services from a 32-year history of foot traffic to a calming virtual presence. Compassion, continuity of care, stakeholder engagement, and respect for strict infection control measures were key drivers behind the rapid development and deployment of virtual services and uninterrupted humanitarian aid for cancer patients during the COVID-19 pandemic. Paradoxically, a reflection on these best practices brought to light some unintended disparities about equitable access to complementary therapies and other supportive care programs. Drawing on the Cedars CanSupport experience, this presentation will describe the gradual adaptation toward a hybrid approach consisting of virtual complementary therapies, biopsychoeducational workshops, humanitarian aid, as well as outreach to other supportive care services (psychosocial support, palliative care, pain, medical cannabis, rehabilitation, lymphedema clinics) during the pandemic. 

## 2. Methodology or Methods

A gap analysis was utilized with particular attention to criteria such as acceptability, availability, awareness, accessibility, and affordability.

## 3. Impact on Practice or Results

As healthcare providers collectively shift practices in a culture-changing COVIDian world, infection control guidelines and live, virtual, or hybrid approaches will become important considerations in the new normal of supportive care programs and services for oncology patients and their families. 

## 4. Discussion or Conclusions

Time will be provided to discuss the personal and professional resources needed to maintain equitable access in the face of ongoing uncertainty and change. 

## 

**58 Developing a Standardized Comprehensive Demographic Data Collection Form to Promote Health Equity in Psychosocial Oncology Research**

## 

TongEryn^1^AnEkaterina^1^AveryJonathan^1^,^2^ShapiroGilla^1^,^3^KubantAlla^1^,^4^MiljanovskiMelissa^1^,^4^ChuAlanna^5^FitzgibbonKyle^1^HalesSarah^1^,^3^BenderJacqueline^1^,^4^1Princess Margaret Cancer Centre, Toronto, ON, Canada2University of British Columbia, Vancouver, BC, Canada3Global Institute of Psychosocial, Palliative and End-of-Life Care, Toronto, ON, Canada4University of Toronto, Toronto, ON, Canada5University of Ottawa, Ottawa, ON, Canada

 

## 1. Background/Rationale or Objectives/Purpose

Background: A key priority for cancer care is to achieve equitable, evidence-informed, and quality health systems. Demographic data collection is fundamental to identifying inequities in care. However, demographic data in psychosocial oncology research remain inconsistently measured and reported. This initiative aimed to develop a standardized and comprehensive demographic data collection form: the Health Equity in Collecting Demographic Information (HECDI). 

## 2. Methodology or Methods

Methodology: The HECDI development process was informed by the Consolidated Framework for Implementation Research. Our working group in the Department of Supportive Care (DSC), Princess Margaret Cancer Centre, conducted an environmental scan of existing resources to inform HECDI development. The form was iteratively revised based on feedback from DSC clinicians, researchers, and leadership. Additional feedback will be obtained from external organizations representing diverse stakeholders. An implementation toolkit is being developed for dissemination. DSC leadership was engaged early in the development process to support future implementation success.

## 3. Impact on Practice or Results

Impact on practice: A standardized demographic data collection form and implementation toolkit will enable the needs of minority populations to be investigated and addressed. Using a standardized form across research studies permits data pooling where necessary and more accurate comparisons across studies. This can allow for the development of inclusive supportive care interventions to improve patient- and family-centered cancer care.

## 4. Discussion or Conclusions

Discussion: We discuss the need for standardized and comprehensive data collection in identifying and addressing inequities in psychosocial oncology research access, participation, and output, and ultimately, clinical care and health outcomes. The next steps include HECDI dissemination across the University Health Network and professional groups in Canada, including CAPO.

## 

**61 Addressing Equity, Diversity, and Inclusion (EDI) in Exercise Oncology Research: The Role of an EDI Committee**

## 

BansalMannatDuchekDelaneyChenAmyWurzAmandaEsterManuelDaunJuliaCulos-ReedNicoleUniversity of Calgary, Calgary, AB, Canada

 

## 1. Background/Rationale or Objectives/Purpose

Currently, limited efforts have been made to address issues of equity, diversity, and inclusion (EDI) within exercise oncology research and subsequent knowledge translation efforts (e.g., exercise programs). To begin to address EDI within this field, the Health and Wellness Lab at the University of Calgary developed an EDI committee. The objectives of this committee are to (i) listen to, acknowledge, and learn from the past offences of systemic and societal racism, discrimination, and oppression; and (ii) make purposeful changes to improve EDI within exercise oncology research and practice.

## 2. Methodology or Methods

The EDI committee drafted an anti-racism statement to declare non-performative allyship, and to clarify the benchmarks to track progress. Initiatives to enhance awareness amongst lab members include EDI meetings twice a month with a focus on: (i) developing an EDI resource document and onboarding checklist; (ii) sharing resources; and (iii) providing EDI workshops to work on addressing EDI issues in exercise oncology.

## 3. Impact on Practice or Results

While the EDI committee is facilitating learning for its lab members, the impact will ultimately be on upcoming and ongoing research and knowledge translation efforts. Addressing EDI issues within exercise oncology research and programs holds potential to remove barriers to engagement and facilitate access to wellness for all individuals living with cancer.

## 4. Discussion or Conclusions

Lab-based EDI committees, such as this one, have the potential to enhance EDI awareness amongst members, foster a space for critical dialogue, and promote learning. Such initiatives may also spur changes in research processes, implementation, and dissemination.

## 

**63 Diversity and Inclusion in Exercise Oncology: Exploring Barriers to Physical Activity among Canadian Ethnic Cancer Populations**

## 

BansalMannatBridelWilliamKing-ShierKathrynCulos-ReedNicoleUniversity of Calgary, Calgary, AB, Canada

 

## 1. Background/Rationale or Objectives/Purpose

Physical activity (PA) interventions can alleviate negative side effects of cancer treatment, and improve quality of life for adults with cancer. Ethnic minorities are underrepresented in PA oncology interventions, despite worse health outcomes than non-minority populations. This highlights the need for supportive care, such as PA interventions, to improve health status for ethnic minorities. Understanding the barriers and facilitators to PA participation is required to more effectively engage ethnic minorities in PA oncology programs. 

## 2. Methodology or Methods

A convenience sample of adults living with cancer, who identify as being part of an ethnic minority group undergoing cancer care at the Tom Baker Cancer Center in Calgary. Through collaboration with patient translation services at the Tom Baker Cancer Center and community-based cultural organizations, ethnic minority groups in oncology will be recruited. Trained interviewers will conduct semi-structured interviews in participants’ preferred language, exploring facilitators and barriers to PA. Interviews will be transcribed verbatim, translated, and analyzed via thematic analysis using Straussian grounded theory.

## 3. Impact on Practice or Results

The experiences of ethnic minorities will provide an understanding of barriers and facilitators to PA participation.

## 4. Discussion or Conclusions

The findings of this study will provide insights into PA participation in ethnic minorities living with cancer. This information will be used to help improve the reach of PA programs to minority populations, fostering PA participation, as well as improved health and quality of life. The proposed research is critical to increase equity and reduce health disparities for all adults living with cancer.

## 

**95 Addressing the Needs of Those Who Identify as Two-Spirit and LGBTQ+, and Are Living with Cancer**

## 

McLeodAlbertUniversity of Winnipeg, Winnipeg, MB, Canada

 

## 1. Background/Rationale or Objectives/Purpose

The concerns, challenges, and needs of Two-Spirit and LGBTQ+ people living with cancer are poorly understood and often underserved in palliative care. This workshop will highlight new tools available to healthcare providers. The goal is to improve palliative and end of life cancer care for 2SLGBTQ+ patients and their caregivers by helping healthcare providers approach their patients in a more open and inclusive way.

## 2. Methodology or Methods

With funding from Health Canada, Canadian Virtual Hospice worked with a diverse group of people who identify as 2SLGBTQ+ and experts in health and palliative care to develop a series of evidence-informed support tools grounded in the lived experience. An initial scoping meeting brought together leading researchers in the field as well as community members to identify needs and gaps that existed in services. A literature review was also conducted to help ensure that the needs were evidence-based and that it identified the information and support needs of caregivers within the SLGBTQ+ community.

## 3. Impact on Practice or Results

Canadian Virtual Hospice worked with members of the 2SLGBTQ+ community to create inclusive care supports. The tools include a healthcare bill of rights, planning for care, choices for care, articles, and a grief learning module.

## 4. Discussion or Conclusions

Clinical implications for healthcare providers are as follows:

- Identify strategies for providing safe, respectful, and responsive care to those who identify as 2SLGBTQ+, as well as to their chosen families and care community.
- Articulate common concerns, challenges, and needs of those who identify as 2LGBTQ+ when accessing palliative care.
- Learn how to ask inclusive questions and avoid making assumptions.
- Access resources to support their practice and empower those who identify as 2SLGBTQ+ to receive quality palliative care.

## 

**96 Tailoring a Physical Activity Program to Meet Equity and Accessibility Challenges: A Qualitative Study Informed by Cancer Survivors and Hospital Professionals**

## 

St-CyrJany^1^,^2^St-OngeKadia^1^,^2^MontminySarah^2^AudreyPlante^2^,^3^PichéAlexia^2^,^4^GauvinLise^2^,^3^DoréIsabelle^2^,^4^1Department of Psychology, Université du Québec à Montréal, Montréal, QC, Canada2Research Centre of the Centre Hospitalier de l’Université de Montréal, Montréal, QC, Canada3School of Public Health, Université de Montréal, Montréal, QC, Canada4School of Kinesiology and Physical Activity Sciences, Faculty of Medicine, Université de Montréal, Montréal, QC, Canada

 

## 1. Background/Rationale or Objectives/Purpose

The purpose of this study was to (1) identify differences in equity and accessibility to a physical activity program for patients in oncology and (2) identify courses of action to tailor implementation to different patient needs and realities.

## 2. Methodology or Methods

Semi-structured interviews were conducted with 21 oncology patients who had completed a physical activity program tailored for people having received a cancer diagnosis as well as with 20 health professionals working in hospital and public health settings in Montreal, Québec.

Interviews were transcribed verbatim, and their content was submitted to an inductive–deductive thematic analysis process. Similar units of discourse were labeled as meaningful codes. The latter were grouped into themes, and themes were cast into categories in an iterative group process including inter-rater agreement and critical analysis team meetings.

## 3. Impact on Practice or Results

Evidence shows that select subgroups of people having received a cancer diagnosis would be more comfortable following a physical activity program in groups limited to their own affiliations and levels of fitness. The greatest challenge noted by participants was integrating physical activity into their occupational schedules and the transition to being active outside of the program.

## 4. Discussion or Conclusions

Program provision could benefit specific subgroups of oncology patients by creating different groups that group younger patients, certain cultural affiliations, persons with varying levels of fitness, and women. Selected accessibility issues could also be overturned by including online provision and most importantly by planning for transition back to a new active work–life balance.

## Acknowledgments

This research was supported by an FSISSS grant from MedTEQ+, which is a partner of the Ministry of Economy and Innovation, Government of Québec.

## 

**114 Research with Indigenous Peoples Living with Cancer: Ethical Considerations, Community Engagement, and Cultural Adaptation in Indigenous Health**

## 

BomfimEmiliana^1^Adams-BeavereyeDakota^2^1McGill University, Montreal, QC, Canada2University of Saskatchewan, Saskatoon, SK, Canada

 

## 1. Background/Rationale or Objectives/Purpose

Persistent health disparities have established biocultural roots with many intersectional aspects playing an integral role in cancer care and survivorship. This work emerged as a result of reflective practices and competencies developed by both authors through lived experience and in their academic journeys, one of the authors being a Canadian Indigenous woman. The purpose of this work is to provide a reflection on the ethical considerations, community engagement approaches, and cultural adaptation principles when conducting research with Indigenous peoples in the context of oncology research, with a particular focus on psychosocial oncology research. 

## 2. Methodology or Methods

This is a concise report that used the CIHR Guidelines for Health Research Involving Aboriginal People as an a priori standard framework to systematically describe authors’ competencies, experiences, and recommendations in working with Indigenous peoples in Canada. 

## 3. Impact on Practice or Results

Four key points were grouped according to data gathered: (1) why cultural adaptation of psychosocial interventions is needed in Indigenous health; (2) what it means to ethically engage with Indigenous peoples for health research; (3) the importance of self-determination in Indigenous health and research; and (4) how research can be used as a tool toward reconciliation. In addition, a few recommendations for future practice and research include (1) Design and adaptation of psychosocial interventions for Indigenous peoples that reflect their unique needs, including accessing culturally sensitive interventions; (2) Incorporating language, traditional spirituality, and peer navigation services; and (3) Exploring the benefits of a trauma-informed approach into cancer care for Indigenous peoples.

## 4. Discussion or Conclusions

The impact that psychosocial oncology research has in closing the gap in health disparities is promising. Future research in this field will lead to tremendous improvements in the health status of racial and ethnic underrepresented cancer patients and survivors.

## 

*Abstract Theme: Cancer Care across the Life Span (Children, Adolescent and Young Adults, Adults, and Older Adults)*

**3 Which Factors May Contribute to Cancer-Related Fatigue and Are They Amenable to Change with Non-Pharmacological Interventions? A Scoping Review**

## 

LevesqueAriane^1^,^2^CaruMaxime^1^DuvalMichel^1^,^3^LaverdièreCaroline^1^,^3^SultanSerge^1^,^2^1Sainte-Justine University Health Center, Research Center, Montreal, QC, Canada2Department of Psychology, University of Montreal, Montreal, QC, Canada3Department of Pediatrics, University of Montreal, Montreal, QC, Canada

 

## 1. Background/Rationale or Objectives/Purpose

We aimed to formulate a definition of cancer-related fatigue (CRF) specific to childhood cancer survivors (CCS). We identified contributors to CRF in CCS to clarify their role as key characteristics of CRF. We reviewed the evidence as to whether these contributors have been addressed by non-pharmacological interventions aiming to reduce fatigue.

## 2. Methodology or Methods

We performed a search in PubMed, PsycINFO, CINAHL, EMBASE, Cochrane Library, Grey Matters, OAlster, and OpenGrey. We used the PRISMA-ScR checklist to ensure systematic reporting. In line with the method recommended by Tricco et al., 2018, data collection and analysis were performed by two independent reviewers and were classified in summary tables. We performed an inductive qualitative thematic analysis of definitions reported by authors using QDA Miner 5.0.

## 3. Impact on Practice or Results

Our search retrieved 1832 articles, of which 36 were identified as pertinent. We identified various contributors to CRF, among which depression symptoms and physical activity level were some of the most commonly cited. Four of the seven identified non-pharmacological interventions were found to target contributors of CRF. The remaining three interventions were found to globally target CRF. 

## 4. Discussion or Conclusions

This review addresses the need to pinpoint contributors of CRF and non-pharmacological interventions aimed at reducing CRF in CCS. Using such information makes it possible to identify knowledge gaps such as unclear definitions of CRF and its contributors, and unclear benefits of non-pharmacological interventions. This review will help direct future research efforts through the formulation of recommendations to better address CRF targets in the context of pediatric cancer aftercare.

## 

**7 Compassion in Pediatric Oncology: A Patient, Parent, and Healthcare Provider Empirical Model**

## 

SinclairShane^1^BouchalShelley Raffin^1^SchulteFiona^1^GuilcherGreg^1^KuhnSusan^1^RapoportAdam^2^PunnettAngela^2^FernandezConrad^3^LetoruneauNicole^1^ChungJoanna^4^1University of Calgary, Calgary, AB, Canada2University of Toronto, Toronto, ON, Canada3Dalhousie University, Halifax, NS, Canada4BC Children’s Hospital, Vancouver, BC, Canada

 

## 1. Background/Rationale or Objectives/Purpose

Compassion is considered a cornerstone of quality pediatric cancer care. However, little is known about the nature, components, and delivery of compassion in pediatric oncology. This study aimed to define and develop a patient, parent, and healthcare provider informed empirical model of compassion in pediatric oncology.

## 2. Methodology or Methods

Pediatric patients (n = 33), parents (n = 16), and healthcare providers (n = 17) were recruited from 4 Canadian academic pediatric hospitals. Data were collected via semi-structured interviews both online (Skype) and in person. Qualitative analysis was guided by Straussian Grounded Theory (SGT), with members of the research team analyzing each transcript in accordance with the three stages of SGT. 

## 3. Impact on Practice or Results

Four domains and 13 related themes were identified, generating the Pediatric Compassion Model, depicting the domains of compassion and their relationship to one another. The four domains include Beneficence, Human Relating, Seeking to Understand, and Attending to Needs. Participants defined compassion as a beneficent response that seeks to address the suffering and needs of a patient and their family through relational understanding, shared humanity, and action.

## 4. Discussion or Conclusions

An evidence-based and clinically informed pediatric model of compassion was generated from this study providing insight into compassion from both those who experience it and those who express it. Future research on compassion in pediatric oncology and healthcare should focus on barriers and facilitators of compassion, measure development, and intervention research aimed at equipping healthcare providers and system leaders with the tools and training to improve it.

## 

**10 Challenges and Difficulties of Saudi Mothers of Children Recently Diagnosed with Cancer**

## 

AlanaziJamilahHuntNigelUniversity of Nottingham, Nottingham, EMA, UK

 

## 1. Background/Rationale or Objectives/Purpose

Despite improvements in medical treatment and increased survival rates, childhood cancer remains a strenuous challenge. The lifestyle of the family is severely disrupted. The situation places an enormous burden on mothers as the primary caregivers. Most research on maternal experience has been conducted in Western countries, and little information is available on the maternal experience of pediatric cancer in non-Western countries. We explore this topic using a new cultural sample of Saudi mothers.

## 2. Methodology or Methods

Semi-structured interviews were conducted to understand maternal experiences of childhood cancer, including the challenges and difficulties the mothers encountered, the impact of the illness, and how they coped with the related stress. Thirty-one mothers whose children were recently diagnosed with cancer participated in this study, and the data were analyzed using a thematic analysis method

## 3. Impact on Practice or Results

Five core themes were extracted: (1) anxiety over the severity of the illness (fear of deterioration of the child’s health condition, fear of the child dying), (2) anxiety about the treatment impact (uncertainty regarding the treatment, fear of chemotherapy), (3) challenges related to the requirements of the treatment (child’s hospitalization, handling the child’s behavioral and emotional problems), (4) effects of the illness (positive and negative), and (5) strategies for coping with the stress. 

## 4. Discussion or Conclusions

This study’s findings can potentially bridge the gap between the needs of the mothers and the healthcare services provided by aiding in the development of psychosocial interventions that address both the challenges faced by the mothers and the issues related to the children’s illness.

## 

**37 Develop a Holistic Strategy to Better Support Adolescents and Young Adults throughout Their Cancer Journey**

## 

KiflenRuhi^1^EdwardsAnnemarie^1^HarrisChristine^1^AveryJonathan^2^JivaniKhairun^3^1Canadian Cancer Society, Toronto, ON, Canada2University of British Columbia, Vancouver, BC, Canada3Canadian Cancer Society, Vancouver, BC, Canada

 

## 1. Background/Rationale or Objectives/Purpose

AYAs diagnosed with cancer face unique challenges. AYAs with cancer do not receive appropriate information and referrals to support services. They experience an intense symptom burden and face challenges in decision making. This project seeks to:

1. Understand the gaps in supportive care and how AYA needs vary amongst geography, cultures, and life stages.
2. Co-design a multi-prong strategy and interventions to address their unmet needs.
3. Evaluate interventions to measure impact on AYA quality of life.

## 2. Methodology or Methods

We used a mixed-methods approach. In Phase 1, we completed a literature review to summarize the supportive care needs of AYAs with cancer. In Phase 2, we are partnering with the Princess Margaret Cancer Center to conduct primary research through a survey and focus groups to co-design solutions. In addition, a multi-disciplinary advisory council will be formed to advise CCS in formulating, implementing, and evaluating a holistic strategy.

## 3. Impact on Practice or Results

In Phase 1, we identified a total of nine unmet needs: career supports; age-specific information; financial burden; psychosocial supports; physical challenges; palliative care; access to clinical trials; supports for fertility services; and self-management. Proven strategies for improving information and support services for AYA include the increasing availability of life stage-specific information, access to peer support programs, and practical supports for financial, fertility, and mental health services.

## 4. Discussion or Conclusions

The needs of AYA vary along the continuum of care, in addition to their age, stage in life, and geographic location. This work provides a foundation to develop an AYA strategy to implement the necessary solutions to better support AYAs with cancer.

## 

**69 Understanding Peer Support-Seeking Behaviors among Adolescents and Young Adults with Cancer using Andersen’s Behavioral Model of Health Services Use**

## 

BabinskiStephanie^1^KhanAmina^1^D’AgostinoNorma^2^ChalifourKarine^3^DrakeEmily^4^GarlandSheila^5^GuptaAbha^2^HowardFuchsia^6^TsimicalisArgerie^7^BenderJacqueline^1^1Cancer Rehabilitation and Survivorship Program, Department of Supportive Care, Princess Margaret Cancer Centre, University Health Network, Toronto, ON, Canada2Princess Margaret Cancer Centre, University Health Network, Toronto, ON, Canada3Young Adult Cancer Canada, St. John’s, NL, Canada4Dalhousie University, Halifax, NS, Canada5Memorial University of Newfoundland, St. John’s, NL, Canada6Univeristy of British Columbia, Vancouver, BC, Canada7McGill University, Montreal, QC, Canada

 

## 1. Background/Rationale or Objectives/Purpose

Adolescents and young adults with cancer (AYA) frequently report feelings of social isolation and a desire to access peer support. Despite this, their uptake of peer support programs is low. We investigate the factors that facilitate and impede peer support seeking among AYA.

## 2. Methodology or Methods

A diverse sample of 17 AYA were purposively selected from a survey sample of AYA recruited from the Princess Margaret Cancer Centre and online. Three semi-structured focus groups were conducted, which were co-facilitated by four patient partners. Focus groups were transcribed verbatim. Data were thematically analyzed, first inductively, and then deductively using Andersen’s Behavioral Model of Health Services Use.

## 3. Impact on Practice or Results

Participants were on average 31 years of age and 5.1 years post-diagnosis, and most (71%) were female. AYA peer support program utilization is influenced by the interactions between individual and contextual pre-disposing and enabling (or disabling) factors, and perceived/influenced need. Predisposing factors include previous social support experiences, fears of emotional vulnerability, and knowledge of support programs. Enabling (or disabling) factors include time, energy, and means to attend programs, and the availability of relevant programs. Factors that influence perceived need for support are existing coping mechanisms and emotional readiness to accept support. Andersen’s theory helps conceptualize the confluence of individual and contextual factors that shape use (or non-use) of peer support programs among AYA. 

## 4. Discussion or Conclusions

AYA peer support programs must be designed to overcome individual and contextual barriers to participation while leveraging and enhancing enabling factors. 

## 

**74 Creating Community and the Way Forward: A Supportive Expressive Therapy Group for People in Their 30s and 40s Navigating the Ways Cancer Changes Life Plans**

## 

MaleDanaPinkJenniferRaithbyTessTom Baker Cancer Centre, Calgary, AB, Canada

 

## 1. Background/Rationale or Objectives/Purpose

People in their 30s and 40s represent a unique demographic within cancer care. A cancer diagnosis during a life stage when significant plans and development (i.e., career advancement, family planning, marriage/dating, personal growth) may be underway can result in paramount experiences of loss, disruption, and isolation. People in this age range may not feel they belong in available support groups tailored toward younger people (i.e., adolescents and young adults) or with an older average age of members. A formative program evaluation will be conducted of supportive expressive group therapy (SEGT) with individuals aged 30–49 years with various cancer types and stages. It is hypothesized that for this population, the ways cancer confronts important life plans represents a more meaningful commonality than diagnosis. 

## 2. Methodology or Methods

Group sessions will be recorded, transcribed, and evaluated using a thematic analysis method. Data collection will continue until saturation is achieved and no new themes emerge. Questionnaires will evaluate pre- and post-psychosocial outcomes (e.g., mood, quality of life, perceived social support).

## 3. Impact on Practice or Results

This study will inform our understanding of the unique impacts of cancer following adolescence/young adulthood and prior to middle-age as well as the effectiveness of SEGT for this specific population. The identification of patient-derived themes will inform an adaptation of the SEGT manual for this population and subsequent iterations of the intervention.

## 4. Discussion or Conclusions

Questions for audience: (1) Are other psychosocial clinicians identifying patients in this age range as having unique unmet psychosocial needs? (2) How might inclusion of different types and stages of cancer influence group cohesion? (3) How can we take a gender-inclusive approach while also promoting group safety and cohesion?

## 

**100 The Impact of the Financial Burden among the Patients with Cancer: A Scoping Review**

## 

KhlifiRabebUniversité de Sherbrooke, Longueuil, QC, Canada

 

## 1. Background/Rationale or Objectives/Purpose

The financial burden of cancer and its treatment causes negative effects on the quality of life among people living with cancer. This study is proposed in order to draw the different elements of the financial burden and its impact on the people living with cancer, especially in Western countries. The reason why those countries were chosen is their similarity at the health system level. 

## 2. Methodology or Methods

A scoping review was based on the method of Peters and Tricco. The principal inclusion criteria were as follows: (1) adults who live with cancer; (2) Western countries; and (3) studies published in the last ten years. However, we excluded (1) children who live with cancer, since the financial burden has an impact on the parents instead; (2) non-Western countries; (3) families of people living with cancer; and (4) health professionals who are associated with the care of patients in cancerology. Two reviewers followed the selection strategy of articles, and the role of the third one is to decide between the non-common articles that were selected.

## 3. Impact on Practice or Results

The preliminary results of this study are (1) reduced income or non-remuneration for a work stoppage during treatment; (2) payment for care and drugs not covered by insurance; (3) other expenses requiring a new lifestyle (e.g., specific diet, transportation costs, caregiver, and home care costs); (4) etc. However, the residence status, which could be one of the factors of financial burden among immigrant people living with cancer, has not been studied. 

## 4. Discussion or Conclusions

This current study would be useful for future studies, such as a study based on the interventional approach to target a decrease of the financial burden among the people living with cancer. 

## 

*Abstract Theme: Complementary and Integrative Cancer Care*

**11 Yoga for Children and Adolescents Affected by Cancer or Blood Disease: Findings from a Systematic Review**

## 

EllisKelsey^1^WurzAmanda^1^McLaughlinEmma^1^SchulteFiona^2^SungLillian^3^Culos-ReedNicole^1^,^2^1Faculty of Kinesiology, University of Calgary, Calgary, AB, Canada2Cumming School of Medicine, University of Calgary, Calgary, AB, Canada3The Hospital for Sick Children, University of Toronto, Toronto, ON, Canada

 

## 1. Background/Rationale or Objectives/Purpose

There is growing evidence regarding the effects of yoga for youth affected by cancer or blood diseases. This systematic review explored patterns across studies and outcomes and identified gaps in knowledge. 

## 2. Methodology or Methods

Inclusion criteria were studies published in English of any study design reporting the effects of a yoga intervention with individuals <18 years with cancer or blood diseases. Eight electronic databases and one trial registry were searched from database inception to February 2021. The included articles’ reference lists were searched for additional articles. Data were extracted, risk of bias was assessed, and summaries were prepared following narrative synthesis guidelines. 

## 3. Impact on Practice or Results

Eleven articles were included from n = 4849 in the initial search, comprising n = 204 participants. Studies consisted of single-group (n = 1) or randomized controlled trial design (n = 10). Most articles (n = 9) included participants who were on treatment for cancer or blood diseases. There was a high variability in outcomes studied and measurement tools used. The most commonly explored outcome was feasibility, which was defined as session attendance and study completion rate. A range of patient-reported (e.g., physical activity levels, sleep, pain, quality of life, anxiety) and clinical outcomes (e.g., length of hospital stay, opioid use) were described.

## 4. Discussion or Conclusions

Findings suggest that yoga is feasible and may enhance quality of life, decrease pain and anxiety, and enhance physical function. Yoga for youth with cancer or blood disease holds potential as a supportive care intervention. Given the number of studies, heterogeneity observed, small sample sizes, and lack of a comparator group, further research is required to determine the effects of yoga for this population. 

## 

**49 Let’s Talk about CAM: Standardizing Assessment and Documentation of Complementary and Alternative Medicine in Oncology**

## 

BalneavesLynda G.^1^WatlingCody Z.^1^HaywardEmilie N.^1^TruantTracy L.O.^2^PorcinoAntony J.^3^Taylor-BrownJill^4^1University of Manitoba, Winnipeg, MB, Canada2University of British Columbia, Vancouver, BC, Canada3BC Cancer, Vancouver, BC, Canada4Canadian Virtual Hospice, Winnipeg, MB, Canada

 

## 1. Background/Rationale or Objectives/Purpose

Complementary and alternative medicine (CAM) is commonly used by cancer patients; however, it is infrequently assessed as part of standard care by oncology healthcare professionals (HCP), hampering efforts to provide safe and informed care. A practice guideline and CAM assessment tool was introduced at CancerCare Manitoba to address this gap in care. 

## 2. Methodology or Methods

Oncology HCPs completed a baseline survey of their knowledge, attitudes, and practices related to CAM and attended an education session about CAM. A standardized CAM assessment form was provided for use in clinical settings. Over the next 4 months, HCPs implemented the form at point of care, with data entered into the electronic health record. HCPs completed a follow-up survey at the end of the study. Changes in HCPs’ CAM knowledge, attitudes, and practices were analyzed using paired *t*-tests. Descriptive analyses summarized CAM therapies and patients’ demographics, as well as uptake of the assessment form by HCPs. Multivariable logistic regressions were conducted to determine factors associated with CAM use. 

## 3. Impact on Practice or Results

A significant positive change in HCPs’ CAM knowledge, readiness to address CAM, and willingness to consult other HCPs about CAM was observed. Among the 2604 patients assessed, 74% reported using at least one CAM therapy. Women, older individuals, those with breast or hematological malignancies, or reporting high anxiety, tiredness, or informational needs were significantly more likely to report CAM use. 

## 4. Discussion or Conclusions

This practice guideline implementation project highlights the feasibility, importance, and impact of standardizing the assessment and documentation of CAM use as part of standard oncology care. 

## 

**55 Promoting Connection, Creativity, and Community: The 3 Cs Fostered by a Chair-Side Creative Art-Making Program in an Outpatient Chemotherapy Treatment Room**

## 

BlackKathleen^1^DuffMiriam^2^1Artists in Healthcare, Winnipeg, MB, Canada2CancerCare Manitoba, Winnipeg, MB, Canada

 

## 1. Background/Rationale or Objectives/Purpose

The value of art programs for adult cancer patients is well documented. This program was created to bring these benefits to the outpatient chemotherapy treatment setting. Advocacy and education to raise awareness about the patient experience and the benefits of arts programming was undertaken to obtain support of clinical staff and secure community funding. 

## 2. Methodology or Methods

Familiarization with infection control and consultation with a hospital-based arts program helped inform planning. An artist facilitator with an oncology nursing background delivered individualized services to patients, empowering them to exercise personal agency in choosing their creative–expressive process. The program has demonstrated known benefits of art making, specifically that it is a multisensory brain-wise activity (Malchiodi, 2008, 2012) that supports cognitive functioning, promotes empowerment, and enables externalization of feelings and relaxation. Ongoing assessment via patient feedback consistently demonstrates increases in creativity, self-expression, communication, relaxation, community, and stress reduction. 

## 3. Impact on Practice or Results

This program has demonstrated success in bringing creative arts into a chemotherapy outpatient treatment setting. The COVID-19 pandemic interrupted this program, creating the opportunity to explore an alternate intervention, offering patients Art Outreach Kits. Met with mixed response, the absence of the artist facilitator suggests this role may be necessary to realize therapeutic outcomes.

## 4. Discussion or Conclusions

This unique program’s success is evidence that artist-facilitated creative outlets in outpatient chemotherapy treatment settings can lead to improved quality of life, reduced anxiety, greater sense of connection, and community for participants. Noting very few supports available in the adult outpatient setting, this program offers an innovative means of integrating complementary care.

## 

**82 Virtual Reality Guided Mindfulness for Chronic Pain in Cancer Survivors: Protocol for a Single-Group Feasibility Trial**

## 

BaydounMohamad^1^BirnieKatie^1^PattonMichaela^1^GreenCraig^2^SzewczykPaulina^2^CartwrightStephen^1^CarlsonLinda^1^1University of Calgary, Calgary, AB, Canada2Patient Research Partner, Calgary, AB, Canada

 

## 1. Background/Rationale or Objectives/Purpose

Chronic cancer-related pain (CRP) has a significant negative impact on quality of life (QOL). Mindfulness is hypothesized to mitigate chronic CRP by regulating emotional reactions to pain, and it is already being used by many cancer survivors in order to improve QOL. In recent years, there has been an interest in the use of virtual reality to deliver mindfulness meditation. Virtual reality provides an immersive environment that may enhance one’s focused attention to present moment experiences, potentially making mindfulness less effortful and more efficacious for patients. There has been little research in this area for people with a history of cancer. The aim of this mixed-methods study is to evaluate the feasibility of a virtual reality guided mindfulness (VRGM) intervention offered to adult cancer patients with chronic CRP. Secondary objectives are to evaluate the benefits and mechanisms of action of VRGM and to explore participants’ experiences with study participation.

## 2. Methodology or Methods

This mixed-methods feasibility study will employ a single-arm, pretest–post-test design with semi-structured interviews. Fifteen cancer survivors will be enrolled in a 6-week, home-based intervention that consists of 15–30 min of daily VRGM practice. The primary outcome is feasibility as assessed by accrual rates, retention, adherence, questionnaire completion, and side-effect rates. Participants will be assessed on psychosocial outcome measures (primary outcome: pain) before and after the intervention and 6 weeks post-intervention. Qualitative information will provide subjective detail to complement quantitative data.

## 3. Impact on Practice or Results

Results can provide the foundation for hypotheses to be tested in future research. 

## 4. Discussion or Conclusions

This research may shed light on the feasibility and potential efficacy of a novel platform for mindfulness meditation delivery, which may enhance its benefits and practice sustainability for hard-to-treat problems such as chronic CRP.

## 

**85 Mindfulness-Based Cancer Recovery and Quality of Life in Cancer Patients: Is Childhood Trauma a Moderator?**

## 

NguyenTinaOberoiDeveshCarlsonLindaUniversity of Calgary, Calgary, AB, Canada

 

## 1. Background/Rationale or Objectives/Purpose

Pain, fatigue, sleep disturbances, mood disturbances, and decline in cognitive functions are side effects often seen due to cancer treatment. Most cancer patients will experience one or more of these side effects, which could significantly impact their quality of life. Mind–body interventions, such as mindfulness-based interventions, have proven to be effective in reducing the side effects cancer patients experience. The primary objective of this study is to investigate the effect of mindfulness-based cancer recovery (MBCR) on cancer patient’s quality of life. The secondary objective is to determine the moderation effects of childhood trauma between MBCR and quality of life of cancer patients.

## 2. Methodology or Methods

This is a subgroup analysis of the data from the Mindfulness-based cancer recovery and Tai Chi/Qigong (TCQ) for Cancer Health (MATCH) study. The MATCH study was a multi-site preference-based waitlist-controlled trial of MBCR vs. TCQ in cancer patients. Participants (N = 262) assigned to the mindfulness intervention in both the immediate and waitlist groups of the MATCH study were included in the current analysis. The immediate (experimental) group will be compared to the waitlist (control) group at baseline and post-intervention using Linear Mixed Modeling with quality of life as the primary outcome. 

## 3. Impact on Practice or Results

Preliminary analysis showed a significant group–time interaction for quality of life. Analysis of the moderating effect of childhood trauma is still in progress.

## 4. Discussion or Conclusions

Preliminary results indicated an increase in the quality of life of cancer patients who participated in MBCR (immediate) compared to the control (waitlist) group. Although the analysis is still in progress, the results of this study could be used to help guide how interventions are provided in the future to cancer patients with a history of childhood trauma.

## 

**91 The Use of Prebiotic and Probiotic Interventions for Treating Gastrointestinal and Mental Health Issues in Cancer Patients and Survivors: A Systematic Review**

## 

DeleemansJulie^1^GajtaniZenel^2^PiedalueKatherine-Ann^2^BaydounMohamad^2^CarlsonLinda^1^1University of Calgary Cumming School of Medicine, Calgary, AB, Canada2University of Calgary, Calgary, AB, Canada

 

## 1. Background/Rationale or Objectives/Purpose

Objective: Cancer treatments can cause significant gastrointestinal (GI) health issues and negatively affect patient’s mental health and quality of life. Novel, integrative strategies using prebiotics and probiotics have been explored for treating adverse treatment-related side effects. We evaluated the current literature for interventions using prebiotics or probiotics specifically to treat GI and mental health issues in cancer patients and survivors.

## 2. Methodology or Methods

Methods: Five databases (PubMed, MEDLINE (Ovid), CINHAL, PsychINFO, Web of Science) and gray literature were searched for studies with prebiotics or probiotics where GI and/or mental health outcomes were measured in adult, human cancer patients and survivors, and published before November 2020. 

## 3. Impact on Practice or Results

Results: Nine studies (N = 688 participants) meeting the inclusion criteria were identified (randomized controlled trials (n = 8), single-group pre–post studies (n = 1)). Six studies were done with patients on active cancer treatment, and three studies treated patients after anti-cancer therapies. Three studies used prebiotics, five studies used probiotics, and one study used a combination therapy. There was minimal evidence for effects of prebiotics on improvements in GI or mental health. Probiotics were associated somewhat with improvements in functional well-being (n = 1), fatigue (n = 1), and quality of life (n = 1). Probiotics were associated most with significant improvements in abdominal pain (n = 2), gas, bloating (n = 2), and especially diarrhea (n = 4).

## 4. Discussion or Conclusions

Conclusions: Studies specifically examining effects of prebiotics and probiotics on GI and mental health outcomes are scarce. Probiotic intervention may improve some GI health issues in cancer patients and survivors. Controlled trials with larger cohorts that also measure mental health outcomes are needed.

## 

**94 Effects of Online Mindfulness-Based Cancer Recovery on Common Chemotherapy Side Effects**

## 

AbolhosseiniShokouh^1^FlynnMichelle^1^SubnisUtkarsh^1^PiedalueKatherine-Anne^1^LupichukSasha^1^TangPatricia^1^WoleverRuth^2^CarlsonLinda^1^1University of Calgary, Calgary, AB, Canada2Vanderbilt University, Nashville, TN, USA

 

## 1. Background/Rationale or Objectives/Purpose

Cancer survivors undergoing chemotherapy experience poor quality of life due to side effects including fatigue, sleep disturbance, and cognitive decline. Previous research supports the efficacy of Mindfulness-Based Cancer Recovery (MBCR) in alleviating these symptoms following treatment. However, MBCR has not previously been investigated as an adjunctive treatment during chemotherapy. Although MBCR is often delivered in-person, online adaptations of the program demonstrate similar efficacy and increase treatment accessibility. The current study evaluated an online MBCR program delivered to cancer survivors during chemotherapy in terms of effects on self-reported fatigue, sleep disturbance, and cognitive decline. 

## 2. Methodology or Methods

This study is a secondary analysis of a randomized waitlist-controlled trial comparing online MBCR to a waitlist in stage I–III breast or II–III colorectal cancer survivors undergoing chemotherapy. Participants (MBCR, n = 13; waitlist, n = 7) received 12 weekly real-time sessions of mindfulness training on www.emindful.com. Participants self-reported fatigue (FACIT-F), sleep disturbance (PSQI), and cognitive decline (FACT-Cog) at baseline and post-intervention (MBCR) or post-wait (waitlist).

## 3. Impact on Practice or Results

Clinical significance was determined using previously established minimal clinically important differences (MCIDs) for fatigue (MCID = 3.00), sleep disturbance (MCID = 1.18), and cognitive decline (MCID = 6.00). More participants in the MBCR group reported clinically significant improvement in fatigue compared to the waitlist group (61.5% vs. 28.6%). No differences between groups were observed for sleep and cognitive function.

## 4. Discussion or Conclusions

This study provides preliminary evidence that online MBCR may be an effective intervention for reducing fatigue among cancer survivors undergoing chemotherapy. Future studies with larger samples and diverse cancer types are needed to further investigate the effects of online MBCR during chemotherapy.

## 

*Abstract Theme: Community-Based and Volunteer Cancer Care Services*

**14 Understanding What They Want and What Works: Supportive Care Needs and Preferences among Men Experiencing Cancer**

## 

MontielCorentin^1^PichéAlexia^2^BedrossianNathalie^2^DoréIsabelle^2^,^3^1Université du Québec à Montréal, Montréal, QC, Canada2Université de Montréal, Montréal, QC, Canada3Centre de recherche du CHUM, Montréal, QC, Canada

 

## 1. Background/Rationale or Objectives/Purpose

Support is essential to psychosocial functioning across the lifespan, even more during critical and stressful life events such as cancer diagnosis and treatments. However, male cancer patients are typically less likely to access available supportive care services compared to their female counterparts. This suggests that available interventions for cancer patients are not adjusted to men’s needs or preferences. The current study aims to build a better understanding of men’s experiences in order to develop appropriate supportive care interventions following the critical life event of cancer. 

## 2. Methodology or Methods

A total of 45 men (15 per age group: 18–39, 40–54, 55+) from the province of Québec diagnosed with cancer are being recruited through hospital oncology services, cancer associations, and community partners. Virtual focus groups and one-on-one telephone interviews with men are being conducted and transcripted, with data collection expected to be completed by March 15.

## 3. Impact on Practice or Results

Using directed content analysis, experience with cancer in terms of its impact on their lives, resulting needs, support experiences, and preferences will be coded using NVivo 12.

## 4. Discussion or Conclusions

This presentation will discuss forthcoming results and will highlight how men’s needs and support preferences stand out and interrelate. The presentation will also introduce how these results will be used, in collaboration with local community stakeholders and healthcare professionals, to develop (1) a pan-Canadian longitudinal survey and (2) supportive care pilot interventions that address and are well adapted to men’s needs and preferences in the context of cancer diagnosis and treatment.

## 

*Abstract Theme: Sociodemographic, Culture, and Sex/Gender Issues in Cancer*

**18 A Scoping Review of the Psychosocial Needs and Experiences of Transgender and Gender-Diverse Individuals Diagnosed with Cancer**

## 

SquiresLauren^1^BilashTristan^2^KamenCharles^3^GarlandSheila^1^,^4^1Memorial University, St. John’s, NL, Canada2Allan Blair Cancer Centre, Regina, SK, Canada3University of Rochester, Rochester, USA4Beatrice Hunter Cancer Research Institute, Halifax, NS, Canada

 

## 1. Background/Rationale or Objectives/Purpose

The psychosocial needs and experiences of transgender and gender-diverse (TGD) patients is an understudied area of oncology research. In response to calls to action from past researchers, we conducted a scoping literature review to provide an overview of the available research evidence. 

## 2. Methodology or Methods

Databases such as PubMed and PsycINFO were searched for studies published from January 2000 to August 2020 on the experiences of TGD people diagnosed with cancer. Following the literature search and initial analysis of the nine included studies, a consultation of key stakeholders (i.e., TGD people with lived cancer experience, people who work to support and advocate for the TGD community, and researchers in the area of TGD healthcare) was conducted via an online survey to gain important feedback to inform our final recommendations.

## 3. Impact on Practice or Results

The following key themes were identified from the nine included articles: (1) lack of coordination between gender-affirming care and cancer care; (2) impact of cancer care on gender affirmation; (3) navigating gendered assumptions; (4) variation in providers’ understanding of patients’ needs; and (5) lack of trans-specific cancer resources. Based on this literature review and stakeholder (n = 18) feedback, we offer recommendations for future research and clinical practice to ensure that the psychosocial needs of TGD patients are met.

## 4. Discussion or Conclusions

The present review is an important starting point for future research in the area of TGD cancer care. Sex and gender can play an integral part in the cancer journey, so the psychosocial experiences of TGD people diagnosed with cancer are often very different from those of their cisgender counterparts. It is essential that this discrepancy is reflected in the literature and clinical practice. 

## 

**33 Co-Creating Culturally Safe Cancer Survivorship Care with Pikwàkanagàn First Nations Community**

## 

GiffordWendy^1^DickPeggy^2^RolfeDanielle^1^ThomasRoanne^1^AwarZeina Al^1^GrahamIan^1^,^3^1University of Ottawa, Ottawa, ON, Canada2Algonquins of Pikwàkanagàn Health Services and Family Health Team, Pikwàkanagàn First Nation, ON, Canada3Ottawa Hospital Research Institute, Ottawa, ON, Canada

 

## 1. Background/Rationale or Objectives/Purpose

Cancer amongst First Nations people in Canada is increasing faster than overall Canadian rates. While First Nations peoples have demonstrated resiliency to the historical effects of colonization, the lack of culturally safe and responsive healthcare services have profoundly impacted trust and the utilization of health services. They continue to experience racism and distrust, and little has been done to incorporate their knowledge and values into cancer survivorship care. This study explored culturally safe healthcare practices to support cancer survivorship healing with Pikwàkanagàn First Nation, which is an Algonquin community in Ontario.

## 2. Methodology or Methods

The study was conducted in partnership with Pikwàkanagàn First Nation. Two focus group discussions were held with cancer survivors and family members (n = 16) and healthcare providers (n = 12). *Journey mapping* procedures were used to conceptualize culturally safe cancer survivorship care and barriers and supports to receiving it. Semi-structured individual interviews (n = 13) were held to further explore patient journeys. Discussions were audio- and video-recorded, transcribed verbatim, and analyzed thematically.

## 3. Impact on Practice or Results

Themes encompassed (1) broad definitions of family; (2) care for caregivers; (3) trauma-informed bereavement; (4) culture as healing; and (5) stories as cultural teachings. Health system barriers included institutional racism and lack of early, well-connected services within and between hospital and community.

## 4. Discussion or Conclusions

Deficit-based perspectives continue to inform Canadian healthcare systems. In our research, we have exposed new ways of thinking about culturally safety in cancer survivorship care. Sharing stories can create a legacy for individual and community healing, and radical innovative approaches are necessary to revolutionize and reconstruct culturally safe care with First Nations people.

## 

**41 Barriers and Challenges to the Breast Cancer Screening among Recent Immigrant Women from Northwest Africa (Maghreb) in Montreal: A Qualitative Study**

## 

KhlifiRabebUniversité de Sherbrooke, Longueuil, QC, Canada

 

## 1. Background/Rationale or Objectives/Purpose

Compared to immigrant women who have lived in Canada for more than 10 years and Canadian-born women, the use of mammography screening remains low among immigrant women who have lived in Canada for less than 10 years (respectively, 70% and 74.2% vs. 42%). This study aimed to identify the barriers and challenges to the use of mammography screening among recent immigrant women in Montreal, Canada.

## 2. Methodology or Methods

According to the conceptual framework of access to healthcare, qualitative research involving face-to-face interviews was conducted in Arabic or French (according to women’s preference) with twelve recent immigrant women aged 50 to 69 from Northwest Africa (Tunisia, Algeria, and Morocco) currently residing in Montreal. Each semi-structured interview lasted between 60 and 90 min. Interview data (verbatim) were translated into English and analyzed using the QDA Miner software.

## 3. Impact on Practice or Results

The results were grouped into five major themes to describe the barriers and the challenges to the mammography screening: “perception of needs and desire for screening”, “breast cancer screening seeking”, “breast cancer screening reaching”, “breast cancer screening utilization”, and “breast cancer screening consequences”.

## 4. Discussion or Conclusions

This study showed that the most important barrier to accessing mammography services was suboptimal recognition and accommodation of the unique ethnocultural needs of Maghrebi immigrant women by the healthcare system. These difficulties were further amplified by poor access to family doctors and healthcare system as well as the women’s economic circumstances.

## 

**42 Barriers to Breast Cancer Screening among Immigrant Women: A Literature Review**

## 

KhlifiRabebUniversité de Sherbrooke, Longueuil, QC, Canada

 

## 1. Background/Rationale or Objectives/Purpose

The breast cancer screening remains low among immigrant women from low and middle-income countries compared to non-immigrant women. We aimed to provide an update on the use of screening and the barriers that prevent immigrant women from undergoing the breast cancer screening using recently published studies.

## 2. Methodology or Methods

We conducted a literature review of the attitudes, knowledge, and practices regarding breast cancer prevention among immigrant women published since 2006 in the following databases: (1) biomedical and medical sciences’ databases: MEDLINE (Ovid), PubMed, EMBASE (Ovid), EMB Reviews (Ovid), CINAHL (EBSCO), and (2) social and human sciences’ databases: International Bibliography of the Social Sciences (IBSS), PAIS Index, Sociological Abstracts, Worldwide Political Science Abstracts, Social Sciences Citation Index (SSCI), Conference Proceedings Citation Index, and Social Science & Humanities (CPCISSH). We restricted our review to articles published between 2006 and 2016. There were no restrictions on language or type of publication.

## 3. Impact on Practice or Results

Out of 2235 articles found, 31 met our inclusion criteria. Using Lévesque’s patient-centered access to healthcare framework, the data were organized into five major categories: (1) “perception of needs and desire for screening”, (2) “breast cancer screening seeking”, (3) “breast cancer screening reaching”, (4) “breast cancer screening utilization”, and (5) “breast cancer screening consequences”.

## 4. Discussion or Conclusions

A culturally tailored breast cancer screening program might increase the participation of immigrant women in screening programs, but the major barriers such as a lack of knowledge and lower socioeconomic levels must also be addressed (e.g., a bigger campaign/more activity to increase knowledge; adapt messages to fit literacy levels, etc.).

## 

**48 Rural–Urban Disparities in Psychosocial and Financial Well-Being among Young Adult Cancer Survivors: A Yacprime Study**

## 

TulkJoshua^1^EatonGeoff^2^ChalifourKarine^2^GarlandSheila^1^,^3^1Department of Psychology, Memorial University of Newfoundland, St. John’s, NL, Canada2Young Adult Cancer Canada, St. John’s, NL, Canada3Discipline of Oncology, Memorial University of Newfoundland, St. John’s, NL, Canada

 

## 1. Background/Rationale or Objectives/Purpose

Rural-dwelling cancer survivors tend to report worse quality of life compared to urban-dwelling cancer survivors, including worse disease severity, greater mood disturbance, and financial burden. Young adult (YA) cancer survivors may be similarly susceptible to the psychosocial and financial difficulties of cancer. Few studies examine rural–urban differences among YAs.

## 2. Methodology or Methods

The Young Adults with Cancer in their Prime (YACPRIME) study recruited YA cancer survivors aged 15 to 39 years. Participants completed the Kessler Psychological Distress Scale (K10), Short Form Health Survey–12 (SF-12), Medical Outcomes Study Social Support Survey, and select survey questions on time off, connection to the YA community, out of pocket expenses, debts, assets, and ability to afford unexpected expenses. Independent samples *t*-tests and chi-squared tests examined group differences among variables. Hierarchical regressions examined moderators of the rurality–distress relationship.

## 3. Impact on Practice or Results

Twenty-five percent of participants (N = 508) reported they lived in a rural or remote area. The total sample had a mean age of 32.2 years (SD = 4.8). Rural-dwelling YA cancer survivors reported significant worse distress (t(468) = 2.82, *p* = 0.005, d = 0.30) and perceived physical health (t(396) = −2.06, *p* = 0.04, d = 0.23) than their urban-dwelling peers. Lower social support moderates experiences of elevated distress among rural YAs (β = −0.19, 95% CI (−0.155, −0.004), *p* = 0.04).

## 4. Discussion or Conclusions

Cancer care programs and providers must consider the unique concerns of YAs with cancer who live in rural areas. Treatment for rural-dwelling YAs with cancer may focus on connecting YAs with appropriate support networks.

## 

**76 Unmet Needs of Sexual and Gender Minorities in Psychosocial Oncology Care: A Scoping Review**

## 

Schulz-QuachChristian^1^,^2^ShapiroGilla^1^,^2^AlkhenainiHamad^1^MoritaJody^1^,^2^MathewAndrew^1^,^2^KennedyMargo^2^WaiAlan^1^,^2^1University of Toronto, Toronto, ON, Canada2University Health Network, Toronto, ON, Canada

 

## 1. Background/Rationale or Objectives/Purpose

Objectives: Sexual and gender minorities (SGM) face unique burdens of cancer that have a profound impact on mental health. The purpose of this review is to identify unmet needs of SGM patients in psychosocial oncology settings, review knowledge gaps in the existing literature, and guide future directions for research and tailored supportive interventions in SGM.

## 2. Methodology or Methods

Using the Arksey and O’Malley framework, a scoping review was conducted to identify relevant published and unpublished reviews. Systematic search of key words, abstracts, and titles was conducted in Pubmed/Medline. Studies were assessed for study type (quantitative, qualitative, and mixed), number of articles included, and year published. Thematic analysis was utilized to identify main themes.

## 3. Impact on Practice or Results

Seven systematic reviews summarizing 153 articles were identified. Main themes included (1) healthcare providers’ lack of knowledge, comfort, and confidence in caring for SGM patients; (2) barriers to patients disclosing their gender identity or sexual orientation; (3) SGM patients experience of discriminatory clinical encounters; (4) lack of inclusive support groups; and (5) heteronormative models of care.

## 4. Discussion or Conclusions

There is a substantial research gap in the existing literature on SGM patients’ psychosocial oncology needs. Most reviews recommended emphasizing an inclusive approach to future service development strategies. Limitations in the available literature include small sample sizes and a lack of population-based data. Future tailored supportive interventions are needed to (1) address SGM disparities, (2) improve mental health outcomes in SGM patients with cancer, and (3) inform the relationship between healthcare providers and SGM patients.

## 

**77 Personality as a Predictive Factor for Mental Distress in Patients with Prostate Cancer**

## 

IlieGabrielaDalhousie University, Halifax, NS, Canada

 

## 1. Background/Rationale or Objectives/Purpose

Prostate cancer patients face a number of psychosocial stressors predisposing them toward the development of poor mental health. Given the longevity of many patients following a diagnosis of prostate cancer, with 10-year survival rates > 70%, many patients are at risk for the development of mental health disorders throughout the duration of their care. Personality traits have been previously examined in oncology patients, with high neuroticism being associated with maladaptive health behaviors and physical health. The current study examines the relationship between personality characteristics, urinary symptoms, and psychologic distress in prostate cancer survivors.

## 2. Methodology or Methods

This study examined 47 men in the Canadian Maritime provinces who received active treatment for prostate cancer from May 2017 to December 2019 and completed an online quality of life survey. The ten-item personality inventory (TIPI) is a Likert-style survey that approximates the Big Five domains of personality: extraversion, agreeableness, conscientiousness, emotional stability (or neuroticism), and openness to experiences. Mental distress was measured using the Kessler psychological distress scale (K10). Urinary symptoms were assessed using the International Prostate Symptom Score (IPSS). 

## 3. Impact on Practice or Results

In the linear regression model, urinary symptoms (sr^2^ = 0.17) and neuroticism (sr^2^ = 0.14) accounted for the highest amount of variance of the outcome predicted by the model. Using logistic regression, personality characteristics were not found to be associated with choice of treatment modality.

## 4. Discussion or Conclusions

Prostate cancer patients with higher neurotic personality traits, who experience worse urinary symptoms, are predisposed toward experiencing higher mental distress during their survivorship. These findings suggest that multidisciplinary care is essential for the management of these patients, as well as screening for mental health issues and urinary symptoms.

## 

*Abstract Theme: Digital Health and Cancer Care*

**70 It’s Time to Address Fear of Cancer Recurrence in the Family Caregiver: Usability Study of an Online Version of the Fear of Recurrence Therapy (FORT)**

## 

LamarcheJani^1^LebelSophie^1^NissimRinat^2^AveryJonathan^3^MaheuChristine^4^LambertSylvie^4^LaiznerAndrea^5^JonesJennifer^2^EsplenMary Jane^6^WongJiahui^6^BaruchMelanie^7^TrachtenbergLianne^2^WoodcockJoyWulfSusanDrziakRosemaryPinciveroNatalie1University of Ottawa, Ottawa, ON, Canada2Princess Margaret Cancer Centre, Toronto, ON, Canada3University Health Network, Toronto, ON, Canada4McGill University, Montreal, QC, Canada5McGill University Health Centre, Montreal, QC, Canada6De Souza Institute, Toronto, ON, Canada7Cancer Care Manitoba, Winnipeg, MB, Canada

 

## 1. Background/Rationale or Objectives/Purpose

Family caregivers (FC) of cancer patients report equal or greater levels of fear of cancer recurrence (FCR) compared to cancer survivors. Due to the lack of literature on effective interventions for FCR in FC and the unique challenges face by FC, the present study aimed to adapt a group intervention called Fear of Recurrence Therapy (FORT) for this population. An advisory board was created to adapt FORT (its format and manuals) for family caregivers and to an online format. A usability study is now necessary to determine the acceptability and feasibility of this updated intervention (FC-FORT). 

## 2. Methodology or Methods

Family caregivers (N = 6–8) and therapists (N = 3) will be recruited through the University of Ottawa, the Princess Margaret Cancer Centre, the Cancer Chat mailing list, and social media. Participants will attend a pre-therapy meeting and complete a 6-week FC-FORT intervention via videoconference. After each session, participants will be asked to complete a short session feedback questionnaire. At the end of the intervention, they will take part in a brief exit interview. 

## 3. Impact on Practice or Results

Data have not yet been collected. However, we plan to present results relating to the usefulness, usability, desirability, value, accessibility, credibility, and general readiness of the sessions, as well as participants’ impressions of the online format and features.

## 4. Discussion or Conclusions

This project is the first to address FCR in FC. The results from this usability study will be presented back to the advisory board in order to further refine the FC-FORT content/format and guide this project’s upcoming pilot study.

## 

**71 Highlighting the Success of Breast Reconstruction Awareness (BRA) Day 2020: A Virtual Platform of Education, Awareness, and Support for Breast Reconstruction Surgery Post-Mastectomy**

## 

KhanRumaisa^1^FrattaroliSamantha^2^BrownMitchell^1^BurnettLaura^2^HeiseyRuth^1^GoulbourneElaine^1^RinaldoEmma^1^WuMelinda^1^MlodzikKate^1^RoebuckJaimie^1^1Women’s College Hospital, Toronto, ON, Canada2Canadian Cancer Society, Toronto, ON, Canada

 

## 1. Background/Rationale or Objectives/Purpose

Less than one in five women eligible for breast reconstruction following mastectomy undergo this surgery. Although many choose not to reconstruct for various reasons, there is a gap in awareness around reconstruction as an option to restore breast shape post-mastectomy.

## 2. Methodology or Methods

Breast Reconstruction Awareness (BRA) Day has empowered patients, caregivers, and healthcare providers with knowledge around the reconstruction process since 2011. In 2020, BRA Day went virtual with a national reach. In addition to traditional information sessions delivered by clinician experts, attendees benefitted from a discussion featuring patients, clinical social workers, and psychologists around strategies for decision making, coping and body positivity. Through a diverse virtual Show and Tell Lounge, images and 360-degree videos of actual reconstruction results were also displayed, allowing attendees to develop realistic expectations and engage with anonymous volunteers whose results were shared.

## 3. Impact on Practice or Results

Most (83%) respondents stated they were more aware of reconstruction options, 93% had a greater understanding of the process, and 80% felt more confident about their choices. Comments included, “BRA Day shares information I can’t find elsewhere and does so in an extremely supportive and unbiased way… it is important for patients to have this venue... it builds a sense of belonging.”.

## 4. Discussion or Conclusions

The success of this event demonstrates that a virtual platform is effective in enhancing reconstruction education and support. To provide more timely information, and to address suggestions for a year-round series, The Peter Gilgan Centre for Women’s Cancers aims to host an ongoing webinar series for reconstruction education in 2021.

## 

**81 Quality and Usefulness of Publicly Available Smartphone Applications for Cancer Caregivers**

## 

WassermanSydney^1^BiningMira^1^BrahimLydia Ould^1^BelzileEric^2^MagalhaesMona^2^LambertSylvie^1^,^2^1McGill University, Montreal, QC, Canada2St Mary’s Hospital, Montreal, QC, Canada

 

## 1. Background/Rationale or Objectives/Purpose

Cancer caregivers are often unpaid family and friends who face numerous challenges and may turn to mobile applications (apps) for support. Although many apps are freely available, their quality has yet to be evaluated. The aim of the study is to evaluate apps that support cancer caregivers in managing their role.

## 2. Methodology or Methods

A search of the iOS Apple App and Android Google Play stores was performed in October 2020 to identify apps for cancer caregivers. Two authors assessed their quality and usefulness using (1) the Mobile App Rating Scale (MARS) tool, (2) the Enlight tool (selected domains), and (3) an unmet needs checklist. Cluster analyses identified apps scoring highly across tools.

## 3. Impact on Practice or Results

In total, 24 apps were evaluated. The mean MARS score was adequate at 65.6% (3.28/5) (SD = 0.55, range: 2.27–4.23); engagement was the highest scoring item, and quality of information was the lowest. The combined score for therapeutic persuasiveness and alliance on the Enlight was fair at 60.7% (3.04/5) (SD = 0.85, range: 1.07–4.5). The privacy and security checklists resulted in means of 77.7% (SD = 18.1, range: 42.9–100.0%) and 41.3% (SD = 35.8, range: 0.0–100.0%), respectively. The unmet needs checklist yielded a mean of 43.3% (SD = 16.2, range: 9.4–69.7%), with the highest scoring item being “Help looking after own health.” The cluster analysis identified 12 apps that scored highly on both the MARS and Enlight. 

## 4. Discussion or Conclusions

Quality and privacy scores are high, whereas usefulness and security need improvement. The quality assessment of these apps will support cancer caregivers in navigating the best available virtual resources.

## 

*Abstract Theme: Exercise/Pre-Habilitation and Rehabilitation in Cancer*

**9 “Exercise Feeds My Well-Being and Keeps Me Stable”: Experiences of Women with Metastatic Breast Cancer and Leisure Physical Activity**

## 

ShallwaniShirinThomasRoanneKingJudyPoitrasStéphaneToupin-AprilKarineUniversity of Ottawa, Ottawa, ON, Canada

 

## 1. Background/Rationale or Objectives/Purpose

The objective of our study was to explore the experiences of people with metastatic cancer related to leisure physical activity (LPA). We also sought to understand the participants’ perspectives, beliefs, facilitators, and barriers, as well as reported preferences related to LPA.

## 2. Methodology or Methods

This qualitative exploratory study was informed by the interpretive description approach. Using an interview guide, we conducted semi-structured interviews via online video-conferencing with seven Canadian women diagnosed with metastatic breast cancer (MBC). Interviews were recorded, transcribed verbatim, and analyzed thematically.

## 3. Impact on Practice or Results

Interview data analysis revealed three important themes: (1) deliberate consideration of cancer- and treatment-related effects and activity modification are often required for women with MBC to be active; (2) participation in LPA is closely linked to psychosocial well-being for many participants; and (3) engaging in LPA positively impacts symptoms, physical function, and treatment tolerance for several women with MBC. 

## 4. Discussion or Conclusions

While the experiences of LPA for women with MBC are personal, participation in LPA frequently involves important consideration of medical effects related to metastatic cancer and its treatments. The psychosocial and physical effects of LPA are especially important for women with MBC. These findings highlight the distinct experiences and perspectives of women with MBC related to LPA and can help inform clinical and research efforts in the area of cancer rehabilitation.

## 

**15 Participant Perspectives of In-Person and Online Exercise Oncology Programs**

## 

DuchekDelaneyCulos-ReedNicoleBridelWilliamMcDonoughMeghanUniversity of Calgary, Calgary, AB, Canada

 

## 1. Background/Rationale or Objectives/Purpose

Exercise oncology participants transitioned from an in-person to an online exercise program due to COVID-19. Understanding differences in exercise setting preferences, exercise barriers and facilitators, and use of behavior change techniques will facilitate understanding of how to optimize the delivery of exercise oncology programs and thus enhance outcomes. 

## 2. Methodology or Methods

**Sample and setting:** Participants who have completed both in-person and online Alberta Cancer Exercise (ACE) classes were contacted to complete a survey. Of n = 124 potential participants, n = 57 (46%) completed the survey. Procedures: An online survey assessed participants’ exercise setting preferences, experiences with behavior change techniques, and exercise barriers and facilitators for in-person and online programs. Data were analyzed using descriptive statistics and content analysis.

## 3. Impact on Practice or Results

Exercise Preferences: Most participants indicated preferring in-person programs (60%), followed by online (31%), and no preference (9%). *Exercise Benefits and Barriers:* There were significantly fewer barriers (*p <* 0.01), but also fewer benefits (*p <* 0.01), to exercising online. *Behavior Change Techniques:* Content analysis indicated that generally, the online exercise setting was less conducive to providing social support. Feedback from instructors on technique and goal setting remained relatively constant across both settings.

## 4. Discussion or Conclusions

ACE participants who have participated in both in-person and online programs had fewer barriers to participation but also felt less supported in the online class setting. This was reflected in less behavior change technique support and fewer opportunities for social interaction with other participants. Findings indicate a need to foster supportive online environments for exercise oncology programs to continue to provide adequate exercise opportunities for cancer survivors during COVID-19.

## 

**20 An Exploration of Practices That Facilitate Social Support in Online Exercise Classes for People Living with Cancer**

## 

CraigBobbie-AnnMcDonoughMeghan H.Culos-ReedS. NicoleBridelWilliamUniversity of Calgary, Calgary, AB, Canada

 

## 1. Background/Rationale or Objectives/Purpose

Social support can be facilitated through exercise programs for people living with cancer, but there is limited literature on how best to foster it in the online environment. The purpose of this study is to examine (1) the current practices for training fitness professionals to facilitate social support in online exercise classes, (2) the experiences people living with cancer have with social support from peers and instructors in online exercise classes, and (3) barriers and facilitators for providing (by fitness professionals) and obtaining (by people living with cancer) support in online exercise classes. Feeney and Collins’ (2015) social support theory will function as a comparative context for interpretation and help identify specific interpersonal behaviors that facilitate support in order to inform intervention development.

## 2. Methodology or Methods

This qualitative study will use interpretive description methodology to examine exercise classes and instructor training from multiple perspectives. Data collection will include a review of staff training materials, participant observation of staff training sessions, observing exercise classes, and interviewing fitness instructors and people living with cancer, with a focus on social support facilitation, provision, and experiences. Prior to interviews, participants will complete an online demographic questionnaire and then complete a 60–90 min semi-structured interview. Data will be analyzed inductively and in light of existing social support theory.

## 3. Impact on practice or Results

Data collection is in progress.

## 4. Discussion or Conclusions

The proposed research will provide guidance on specific behaviors to promote and facilitate social support in online physical activity programs. This study will also provide information about ways to improve social support in exercise programs.

## 

**38 A Study Protocol for IMPACT: IMplementation of Physical Activity for Children and Adolescents on Treatment for Cancer or Blood Diseases**

## 

WurzAmanda^1^EllisKelsey^1^McLaughlinEmma^1^GuilcherGregory M.T.^2^McIntyreKrista^2^WilsonBeverly^3^FisherSara^3^Culos-ReedS. Nicole^1^1University of Calgary, Calgary, AB, Canada2Alberta Children’s Hospital, Calgary, AB, Canada3Stollery Children’s Hospital, Edmonton, AB, Canada

 

## 1. Background/Rationale or Objectives/Purpose

Physical activity (PA) can enhance well-being among youth diagnosed with oncological or hematological diseases. We developed a tailored, 1:1, online PA program (IMPACT) to promote PA in this cohort. The proposed single-group, mixed-methods project will assess the effect of IMPACT and explore markers of implementation.

## 2. Methodology or Methods

Youth aged 5–18 years (and at least one parent), with any oncological or hematological diagnosis, who are currently receiving, scheduled to receive treatment, or completed treatment <3 months in Alberta will be eligible to participate. Participants will be offered online PA sessions 2–3 times/week for 15–45 min/session for 8–12 weeks. Measures of PA behavior, physical, psychosocial, and cognitive functioning, medical symptoms, and resource usage will be collected at baseline, post-intervention, and 6 and 12 months after baseline to assess the effect of IMPACT. To explore implementation, metrics such as referral rates, participant adherence, adverse events, and PA fidelity will be collected throughout. Quality improvement interviews will be conducted every 6 months with a purposeful sample. Quantitative data will be analyzed with descriptive statistics, general linear mixed models, multilevel modeling, and responder analysis. Qualitative data will be analyzed with framework analysis using a hybrid inductive–deductive approach.

## 3. Impact on Practice or Results

Results will be among the first examining the effectiveness and implementation of an online PA program for youth with cancer or blood disease.

## 4. Discussion or Conclusions

Online delivery is the only way to offer PA during COVID-19, and it may be a feasible way to promote PA within the complex, highly variable pediatric oncology/hematology treatment timeline. This study could offer insights to improve care for this population.

## 

**90 ACE-Neuro: A Tailored Exercise Oncology Program for Neuro-Oncology Patients**

## 

DaunJulia T.^1^CapozziLauren C.^1^,^2^UrgoitiGloria Roldan^3^McDonoughMeghan H^1^EasawJay^4^McNeelyMargaret^5^,^6^FrancisGeorge J.^2^,^7^WildingMarie de Guzman^8^RadkeLori^8^LesiukChristine^3^Culos-ReedS. Nicole^7^,^9^,^10^1Faculty of Kinesiology, University of Calgary, Calgary, AB, Canada2Department of Clinical Neurosciences, Cumming School of Medicine, University of Calgary, Calgary, AB, Canada3Department of Medical Oncology, Tom Baker Cancer Centre, Calgary, AB, Canada4Department of Medical Oncology, Cross Cancer Institute, Edmonton, AB, Canada5Department of Physical Therapy, University of Alberta, Edmonton, AB, Canada6Department of Oncology, Cross Cancer Institute, Edmonton, AB, Canada7Department of Oncology, Cumming School of Medicine, University of Calgary, Calgary, AB, Canada8Cancer Care Alberta, Alberta Health Services, Calgary, AB, Canada9Faculty of Kinesiology, University of Calgary, Calgary, AB, Canada10Department of Psychosocial Resources, Tom Baker Cancer Centre, Calgary, AB, Canada

 

## 1. Background/Rationale or Objectives/Purpose

Due to the significant burden of disease and its treatments, exercise delivery to the neuro-oncology patient population is complex, creating a gap in patient care. This study will examine the implementation and effectiveness of the Alberta Cancer Exercise Neuro-Oncology program (ACE-Neuro). Specifically, we will examine (1) the impact of an online, tailored exercise oncology program on neuro-oncology patients’ physical and psychosocial outcomes, using both quantitative and qualitative methods, and (2) the effectiveness of the ACE-Neuro patient referral and triage process.

## 2. Methodology or Methods

Referred neuro-oncology patients >18 years will be seen in a study-based Rehabilitation Triage Clinic. Depending on overall health and functional status, patients will be triaged to either ACE-Neuro, rehabilitation oncology, or cancer physiatry. Outcomes related to this triage decision will be tracked. Once referred to ACE-Neuro, participants will complete online assessments of physical function, patient-reported outcomes, and objective physical activity at baseline, post-program completion (12-weeks), and at follow-up timepoints every 3 months until study completion (up to 24 months). ACE-Neuro includes weekly one-on-one online exercise delivery, health coaching to support behavior change, and access to an online group exercise session. Exercise and health coaching delivery will be led by a Clinical Exercise Physiologist. Participants will also be invited to a post-program qualitative interview to get perspectives on their experiences participating in ACE-Neuro.

## 3. Impact on Practice or Results

Recruitment for ACE-Neuro is ongoing until Spring 2023. 

## 4. Discussion or Conclusions

This implementation project will advance exercise oncology research and clinical practice within the cancer care system by working directly with patients, healthcare providers, and community partners to develop a framework that streamlines patient triage and provides a tailored online exercise program for neuro-oncology patients.

## 

*Abstract Theme: Implementation Science, Knowledge Translation, and Synthesis*

**54 Identifying Barriers and Enablers to the Use of Survivorship Care Plans Amongst Cancer Survivors using the Theoretical Domains Framework**

## 

LangmuirTori^1^MutsaersBrittany^2^MacDonald-LiskaCarrie^3^PresseauJustin^1^,^2^LarocqueGail^3^HarrisCheryl^1^,^2^ThavornKednapa^1^ChomienneMarie-Hélène^2^,^4^LebelSophie^2^1The Ottawa Hospital Research Institute, Ottawa, ON, Canada2University of Ottawa, Ottawa, ON, Canada3The Ottawa Hospital, Ottawa, ON, Canada4C.T. Lamont Primary Health Care Research Centre, Ottawa, ON, Canada

 

## 1. Background/Rationale or Objectives/Purpose

Survivorship care plans (SCPs) are recommended as tools to facilitate a smooth transition from tertiary hospital settings back to primary care after active cancer treatment is completed. There is mixed evidence on the effectiveness of SCPs due to variation in the content, format, and implementation. The purpose of this study is to identify barriers and enablers to SCP use among breast and colorectal cancer survivors.

## 2. Methodology or Methods

Twenty-six breast and colorectal cancer survivors who received an SCP from The Wellness Beyond Cancer Program in Ottawa, Ontario will be recruited. Survivors completed 30–45-min telephone interviews. The interview questions and data analysis were based on the Theoretical Domains Framework (TDF) to systematically identify barriers and enablers to SCP use. Directed content analysis was used to code text within the 14 TDF domains, and similar content within each domain were grouped together to create themes. 

## 3. Impact on Practice or Results

Preliminary results have identified four TDF domains representing enablers and barriers to SCP use by survivors. (1) Social Professional Role and Identity (survivors not needing to use SCPs because physicians take the lead in coordinating and providing follow-up care); (2) Knowledge (helpful information included in SCPs); (3) Reinforcement (positive experiences using SCPs increasing likelihood survivors will use their SCP again); (4) Behavioral regulation (suggestions of electronic and updatable formats for SCPs, including resources for social support).

## 4. Discussion or Conclusions

Identifying barriers and enablers to SCP use is an important first step in generating evidence-based recommendations for implementing SCP use across Canadian settings.

## 

**56 Primary Care Providers** **’ Perspectives on Providing Follow-Up Cancer Care: A Qualitative Analysis of Survivorship Care Plan Use**

## 

MairenaPaola Michelle Garcia^1^MutsaersBrittany^1^LangmuirTori^2^MacDonald-LiskaCarrie^3^PresseauJustin^1^,^2^LarocqueGail^3^HarrisCheryl^1^,^2^ChomienneMarie-Hélène^1^,^4^ThavornKednapa^2^LebelSophie^1^1University of Ottawa, Ottawa, ON, Canada2The Ottawa Hospital Research Institute, Ottawa, ON, Canada3The Ottawa Hospital, Ottawa, ON, Canada4C.T. Lamont Primary Health Care Research Centre, Ottawa, ON, Canada

 

## 1. Background/Rationale or Objectives/Purpose

Survivorship care plans (SCPs) encompass a summary of cancer treatment and follow-up care that cancer survivors need to pursue with their primary care providers (PCPs). SCPs are meant to enhance the communication and the organization regarding follow-up cancer care, ameliorate the survivors’ satisfaction, and ensure that survivorship care needs are addressed. This study’s objective was to investigate how primary care providers perceive their roles in providing follow-up cancer care.

## 2. Methodology or Methods

Thirteen PCPs in the Ottawa region who provide follow-up care to breast and colorectal cancer survivors will be recruited across urban and rural settings. Telephone interviews of 15–20 min were conducted with PCPs. The interview guide and directed content analysis were based on the Theoretical Domains Framework (TDF-2). Thematic analysis was used to identify themes and subthemes related to PCP’s views on SCPs and their role in providing follow-up cancer care within each domain.

## 3. Impact on Practice or Results

Preliminary results indicate the relevance of the following TDF domains: Knowledge (PCPs would like additional information in SCPs), Environmental Context and Resources (challenges coordinating care, increased workload), and Social Professional Role and Identity (inconsistencies in communication between PCPs and cancer specialists). 

## 4. Discussion or Conclusions

Understanding the perceptions of PCPs regarding their role in providing follow-up cancer care and SCP use will facilitate the implementation of SCPs as transition tools from tertiary care back to primary care.

## 

**57 Cancer Survivors** **’ Perspectives on Survivorship Care Plans: Qualitative Analysis Examining Differences in Men and Women, and Urban and Rural Settings**

## 

GiguèreLauriane^1^BabikerDina^1^MutsaersBrittany^1^MacDonald-LiskaCarrie^2^PresseauJustin^1^,^3^LarocqueGail^2^HarrisCheryl^1^,^3^ChomienneMarie-Hélène^1^,^4^ThavornKednapa^3^LebelSophie^1^1University of Ottawa, Ottawa, ON, Canada2The Ottawa Hospital, Ottawa, ON, Canada3The Ottawa Hospital Research Institute, Ottawa, ON, Canada4C.T. Lamont Primary Health Care Research Centre, Ottawa, ON, Canada

 

## 1. Background/Rationale or Objectives/Purpose

Survivorship Care Plans (SCPs) are used as transition tools for cancer survivors following active treatment. The objective of this study is to explore differences in how SCPs are used based on sex (male vs. female) and location (urban vs. rural settings in Ontario). 

## 2. Methodology or Methods

Twenty-six breast and colorectal cancer survivors who received SCPs from the Wellness Beyond Cancer Program living in urban or rural settings in or around Ottawa, ON will be recruited. Survivors will be interviewed for 30 to 45 min about their experience with their SCP. The interview was based on the Theoretical Domains Framework. Interviews were transcribed verbatim and coded using content and thematic analysis in NVivo 12.

## 3. Impact on Practice or Results

Results on differences in SCP use between men and women, and urban and rural settings will be presented. Initial analysis indicates that breast cancer survivors use the information in SCPs to manage uncertainty (i.e., referring to treatment summary, contact information) and suggests including additional information about peer support.

## 4. Discussion or Conclusions

Examining similarities and differences between how SCPs are used based on sex and urban vs. rural locations is important for informing recommendations for implementing SCPs. These results can be used to tailor SCPs to best meet any sex/gender-specific and/or location needs of cancer survivors across urban and rural settings.

## 

**66 Structural Factors Shaping the Implementation of a Physical Activity Program in a Clinical Oncology Setting in Montréal, Québec**

## 

Saint-OngeKadia^1^,^2^St-CyrJany^1^,^2^PlanteAudrey^2^,^3^MontminySarah^2^PichéAlexia^4^GauvinLise^2^,^3^DoréIsabelle^2^,^4^1Université du Québec à Montréal, Montreal, QC, Canada2Research Centre of the Centre Hospitalier de l’Université de Montréal, Montreal, QC, Canada3School of Public Health, Université de Montréal, Montreal, QC, Canada4School of Kinesiology and Physical Activity Sciences, Faculty of Medicine, Université de Montréal, Montreal, QC, Canada

 

## 1. Background/Rationale or Objectives/Purpose

Prevention programs shown to be effective in improving the quality of life and treatment experience of oncology patients often aim to reduce structural gaps in service provision. However, little is known about these gaps. Thus, we identified structural barriers and levers to the implementation of physical activity promotion in cancer.

## 2. Methodology or Methods

We conducted semi-structured interviews with 20 health professionals involved in physical activity promotion in a large clinical setting as well as with 21 oncology patients having completed a tailored physical activity program. Interview transcripts were submitted to inductive–deductive thematic analysis. Meaningful units of analysis (codes) were grouped into themes and then into categories in an iterative and collective process including constant inter-rater agreement and critical analysis.

## 3. Impact on Practice or Results

Participants named a need to better inform health professionals about evidence-based physical activity promotion during oncology treatment and how to implement it. Specialized kinesiologists were said to be an essential and yet scarce resource. Multilevel collaboration guided by strong leadership was said to be essential in gathering the necessary resources for program implementation. Greater funding and collaboration from governmental and health institutions was thought to be required in scaling-up the program to a provincial level. Lastly, social norms and expectations of cancer survivorship and treatment could support maintaining physical activity during cancer treatments. 

## 4. Discussion or Conclusions

The success of physical activity promotion in clinical settings appears to depend on multilevel implementation initiatives and partnerships that range from continuing health professional education to political advocacy.

This work was supported by an FSISSS grant from MedTEQ+, which is a partner of the MEI, Government of Quebec.

## 

**88 An Ideal Intervention for Cancer-Related Fatigue: What Do Patients, Community Support Providers, and Healthcare Providers Want?**

## 

RutkowskiNicoleJonesGeordenTrudelGenevièveLebelSophieUniversity of Ottawa, Ottawa, ON, Canada

 

## 1. Background/Rationale or Objectives/Purpose

Patients consistently rate cancer-related fatigue (CRF) as the most prevalent and debilitating symptom. Despite growing awareness of CRF, including the creation of guidelines on treatment and assessment and several evidence-based interventions that have shown efficacy in randomized control trials, sustainable implementation of interventions remain a concern. This needs assessment focused on exploring what an ideal intervention for CRF would look like from the perspective of patients, community support providers, and healthcare providers and the predicted barriers to its implementation in the Ottawa, Canada.

## 2. Methodology or Methods

Nine focus groups and four individual interviews with physicians were conducted using semi-structured interviews. Three participant populations were recruited: healthcare providers (HCPs; n = 32), community support providers (CSPs; n = 15), and cancer patients (n = 16). Data were coded into themes using content analysis. 

## 3. Impact on Practice or Results

The results of the focus groups revealed “one size does not fit all”; participants differed on their views of the location, provider, format, timing, and type of intervention that would be ideal for CRF. Predicted barriers to success included accessibility, online literacy, overload of information, unique patient characteristics, and systemic issues. To ensure sustainability, participants suggested training and engaging professionals or peers on the delivery of the CRF intervention. 

## 4. Discussion or Conclusions

As CRF is a common post-treatment symptom, it is imperative to offer survivors adequate support and resources to manage CRF. This needs assessment lays the groundwork for a community implementation of an evidence-based intervention for CRF in the Ottawa region.

## 

*Abstract Theme: Survivorship*

**5 Decision Making of Women Who Had Surgical Treatment for Breast Cancer: Qualitative Exploration of Patient Perspectives**

## 

EtchegaryHollyDarmonkowGeorgiaMemorial University, St. John’s, NL, Canada

 

## 1. Background/Rationale or Objectives/Purpose

Mastectomy (MT) rates are higher in Newfoundland and Labrador (NL) than in any other province in Canada, even in women diagnosed with early-stage breast cancer. We present qualitative data from women who made a surgical breast cancer treatment decision in order to better understand the decision-making environment and process.

## 2. Methodology or Methods

A descriptive, qualitative design was employed. Semi-structured interviews and focus groups were held with women in NL who underwent surgical treatment for breast cancer, including breast-conserving surgery (BCS) or mastectomy (MT).

## 3. Impact on Practice or Results

Thirty-five women participated. Of these, 74.3% had MT, while only 11.4% had BCS. Additionally, 14.3% had BCS initially followed by MT. The surgical treatment decision making context was heterogeneous. Women reported varying levels of time they had to make a surgical decision, diverse perceptions of decisional choice, opinions on the adequacy of information provided to inform a decision, and different levels of available formal and informal supports. Most reported they were satisfied with their surgical decision, although the context in which these decisions were made was clearly a challenging one.

## 4. Discussion or Conclusions

While most women were pleased with the surgical care they received, adequate time and thorough pre-surgical discussion were noted as necessary—but not always available. Women explained the importance of thinking through their personal circumstances and values so as to make informed surgical decisions. Post-surgical care and discussion of available psychosocial supports were proposed as areas that could be improved. 

## 

**13 Determinants of Cancer-Associated Stigma among Cancer Survivors in Newfoundland and Labrador**

## 

SavasSevtap^1^TenkorangEric Y.^2^WinsorMercy^3^SimmondsCharlene^3^StucklessTeri^4^1Division of Biomedical Sciences, Faculty of Medicine, Memorial University, St. John’s, NL, Canada2Department of Sociology, Faculty of Humanities and Social Sciences, Memorial University, St. John’s, NL, Canada3Health Research Unit (HRU), Faculty of Medicine, Memorial University, St. John’s, NL, Canada4Discipline of Oncology, Faculty of Medicine, Memorial University, St. John’s, NL, Canada

 

## 1. Background/Rationale or Objectives/Purpose

Objectives: We aimed to investigate the determinants (socioeconomic, demographic, and clinical) of cancer-related stigma among patients/survivors in Newfoundland and Labrador (NL).

## 2. Methodology or Methods

Sample and setting: A cross-sectional online survey was conducted from June 2019 to February 2020. Data were collected from 278 cancer patients/survivors. 

Procedures: Cancer-associated stigma was measured using a modified Cataldo lung cancer stigma scale consisting of 25 items. Responses were measured on a 4-point Likert scale. Exploratory Factor Analysis was used to identify the underlying constructs in participants’ responses. Relationships between two stigma constructs (social isolation and self-stigma) and participants’ socioeconomic, disease-related, and demographic characteristics were examined using multivariate regression analyses. 

## 3. Impact on Practice or Results

Results: Younger age (<45 years) was significantly associated with experiencing higher levels of both social isolation and self-stigma. Educational level and employment status were associated with social isolation but not self-stigma. Income levels were associated with both constructs. Interestingly, compared to individuals diagnosed with stage I disease, stage II and III patients experienced higher levels of social isolation, while stage IV patients experienced increased self-stigma. Indigenous persons had a higher risk of social isolation than Caucasians.

## 4. Discussion or Conclusions

Conclusions and Clinical implications: Our findings suggest measurable cancer-associated stigma within the Newfoundland and Labrador population. Specific demographic groups, such as indigenous persons and younger respondents, reported higher levels of stigma. Public health education about cancer-associated stigma should be emphasized in NL, and specific interventions targeting vulnerable groups should be implemented. 

## 

**22 Grief and Gratitude: Experiences of Two Generations of Cancer Survivors**

## 

AlstonNicole^1^SimpkinsNia^2^1Ok2Grieve, LLC, NY, USA2Charlotte, USA

 

## 1. Background/Rationale or Objectives/Purpose

It has been documented that among cancer survivors, hearing the words “all clear” does not mean “all well”. Furthermore, survivors are confronted with numerous challenges in the aftermath of treatment: depression, anxiety, PTSD, cognitive impairment, hearing loss, and many other quality of life issues. These challenges are juxtaposed with the general expectation that survivors should be happy to have fought cancer and won. This unique experiential presentation is an exploration of the lived experiences of two generations of African Americans, a breast cancer survivor, and a neuroblastoma survivor, ten years after being treated at the same time. 

## 2. Methodology or Methods

- Participants will explore aspects of quality of life challenges among cancer survivors, particularly adolescent survivors of color,
- Recognize the parallel between symptoms of prolonged grief disorder and the journey of cancer survivorship,
- Discuss strategies that practitioners may consider to support individuals and families.

## 3. Impact on Practice or Results

- Explore unique aspects of quality of life challenges among cancer survivors, particularly survivors of color,
- Recognize the parallel between symptoms of complicated grief and the journey of cancer survivorship,
- Discuss strategies that practitioners may use to support individuals and families.

## 4. Discussion or Conclusions

Increased preparation and support is needed for survivors, particularly among communities of color, as they re-enter life post-treatment.

## 

**26 Social Functioning in Survivors of Pediatric Acute lymphoblastic Leukemia: A Systematic Review**

## 

ChoSara^1^TromburgCourtney^2^ForbesCaitlin^1^TranAndrew^1^AllapitanElleine^1^SchulteFiona^1^1University of Calgary, Calgary, AB, Canada2University of Alberta, Edmonton, AB, Canada

 

## 1. Background/Rationale or Objectives/Purpose

The objectives of this review were (1) to summarize studies that described social outcomes in survivors of pediatric ALL across the lifespan and (2) to examine associations between treatment and non-treatment related risk and resilience factors on social outcomes. 

## 2. Methodology or Methods

Sample: The databases searched included EMBASE (Ovid), PsycINFO (EBSCO Information Services), and Web of Science (Thomson Reuters). Eligible studies (1) were original research; (2) were published in English; (3) included participants diagnosed with cancer between the ages 0 and 21; (4) described survivors who were at least 5 years from diagnosis and/or 2 years from completion of therapy; and (5) included quantitative assessment of social functioning outcomes. 

Procedures: The review yielded 3698 articles, of which 43 were included in the final review. Risk of bias was assessed using domains adapted from the Cochrane risk-of-bias tool. Quality of evidence was assessed according to the criteria from the Grading of Recommendations Assessment Development and Evaluation (GRADE).

## 3. Impact on Practice or Results

Only six studies included social functioning as a primary outcome. In studies that included a control group (n = 36), 66.7% reported worse social functioning for survivors of ALL compared to controls. There was some evidence (Grade B) suggesting treatment (e.g., cranial irradiation) and non-treatment (e.g., sex) factors were associated with social functioning.

## 4. Discussion or Conclusions

Survivors of pediatric ALL were at higher risk of social functioning difficulties compared to controls. However, evidence for treatment and non-treatment risk and resilience factors require stronger evidence. Information on modifiable factors that modulate social functioning may influence targets of intervention and follow-up guidelines.

## 

**29 Online Participatory Art Workshops: An Opportunity to Develop New Understandings of a Good Life in the Context of Cancer Survivorship**

## 

RheaultAlysson^1^ThomasRoanne^1^BoulangerJosée^1^GrassauPamela^2^EganMary^1^,^3^GiffordWendy^1^KingJudy^1^1University of Ottawa, Ottawa, ON, Canada2Carleton University, Ottawa, ON, Canada3Bruyère Research Institute, Ottawa, ON, Canada

 

## 1. Background/Rationale or Objectives/Purpose

There is a pervasive assumption that life with impairment is inferior to life without. However, emerging research challenges the preconception that life with impairment is associated with poor quality of life. In response to this, our team created a community-based research program to explore how creative practices can contribute to new understandings of a good life in the context of cancer survivorship. Considering the COVID-19 pandemic, we also aimed to explore how qualitative researchers using participatory and art-based methods may deliver similar programs online. 

## 2. Methodology or Methods

We offered a series of four online visual arts workshops to four women living in the Ottawa region who had experienced cancer. The workshops were facilitated by an arts-based survivorship researcher and a visual artist via Zoom. Workshops were video-recorded, whereas the individual pre- and post-workshop interviews were audio-recorded. All recordings were transcribed verbatim. Participants also photographed their artwork. Additionally, field notes were taken to document the research process. Data were analyzed thematically using NVivo12.

## 3. Impact on Practice or Results

Our preliminary analysis reveals that online art workshops present opportunities to (1) meet participants where they are; (2) engage participants in a supportive space; (3) and enhance the participatory nature of research.

## 4. Discussion or Conclusions

While our previous research demonstrates that in-person art workshops can contribute to well-being, our current study highlights the potential positive impact of online workshops as well. This method of delivery may broaden the access to similar cancer survivorship programs for other individuals who otherwise may be excluded from research.

## 

**35 Characterizing Pain in Long-Term Survivors of Childhood Cancer**

## 

PattonMichaela^1^ForsterVictoria^2^ForbesCaitlin^3^StokoeMehak^3^NoelMelanie^1^CarlsonLinda^1^BirnieKathryn^1^ReynoldsKathleen^3^SchulteFiona^1^,^3^1University of Calgary, Calgary, AB, Canada2The Hospital for Sick Children, Toronto, ON, Canada3Alberta Children’s Hospital, Calgary, AB, Canada

 

## 1. Background/Rationale or Objectives/Purpose

Many long-term survivors of childhood cancer (LTSCC) experience late- and long-term effects from their treatments, including pain. Yet, pain is poorly understood among LTSCC. The current study aimed to (1a) describe the prevalence and multiple dimensions of pain; (1b) identify patterns of chronic pain; and (2) test correlates of chronic pain in LTSCC. 

## 2. Methodology or Methods

Survivors (n = 140; 48.6% male, M_age_ = 17.3 years (Range = 8–25)) were recruited from across Canada. Participants completed the Pain Questionnaire, Pain Catastrophizing Scale, Patient-Reported Outcome Measurement Information System (PROMIS)—Pain Interference, Anxiety, and Depression scales, Child Post-Traumatic Stress Scale, and the Post-Traumatic Stress Disorder Checklist for the Diagnostic Statistical Manual of Mental Disorders (Version 5). 

## 3. Impact on Practice or Results

More than one-quarter (26%) of LTSCC reported experiencing chronic pain (i.e., pain lasting 3 months or more). An exploratory cluster analysis showed 20% of survivors had moderate to severe chronic pain based on measures of pain intensity and interference. The combination of higher post-traumatic stress symptoms, older current age, higher pain catastrophizing, and sex (being female) significantly predicted the presence of chronic pain, χ^2^(4, *N* = 107) = 28.10, *p* < 0.001. Higher pain catastrophizing (OR = 1.09; 95% CI = 1.02–1.16), older current age (OR = 1.20; 95% CI = 1.07–1.34), and higher post-traumatic stress symptoms (OR = 1.92; 95% CI = 1.01–3.63) significantly predicted chronic pain. 

## 4. Discussion or Conclusions

LTSCC should be screened for the presence and impact of chronic pain during long-term follow-up visits so that appropriate interventions can be offered and implemented. Future research should investigate pain and trauma interventions tailored for this population. 

## 

**36 Exploring Art Making as a Source of Metaphor for Women’s Cancer Experiences**

## 

NovyChristineRangerMarie-ChristineThomasRoanneUniversity of Ottawa, Ottawa, ON, Canada

 

## 1. Background/Rationale or Objectives/Purpose

As life expectancy following cancer treatment increases, there is a growing need to understand experiences of cancer survivorship. Metaphoric language is widely used in cancer discourse as a framework for understanding, particularly at the time of diagnosis and during treatment. However, research suggests that prevailing military and journey metaphors may gloss over variation in women’s cancer experiences. To address this gap, our team developed an arts-based community research program. Our objective was to explore the relationship between art making and metaphor as a language for self-expression.

## 2. Methodology or Methods

Eleven women participated in the program, which consisted of two series of 3-hour workshops held weekly for 3 weeks. The workshops took place in a cancer survivorship center and were co-led by an arts-based cancer researcher and a visual artist. Pre and post-workshop interviews as well as workshop evaluations were conducted with each participant. Interviews and workshops were recorded and transcribed verbatim. Additionally, each participant’s creative work was photographed. Data were analyzed thematically using NVivo.

## 3. Impact on Practice or Results

Our analysis revealed the ways in which art making enabled the participants to shape their own metaphoric thought and thus contribute a more experiential understanding of their cancer experiences. The following themes and associated metaphors are explored through our poster: (1) imperfection and impermanence, (2) transformation (of identity and outlook), (3) growth and regeneration.

## 4. Discussion or Conclusions

A more in-depth understanding of women’s cancer experiences is needed to inform post-treatment cancer care. Arts-based workshops offer one way for women to explore and articulate their insider knowledge.

## 

**40 Psychosocial Burden among Adolescent and Young Adult (AYA) Cancer Survivors and Tools to Support Care Delivery**

## 

KitchenJessicaDeCariaKristenTomehMarellOliveiraClaire deCanadian Partnership Against Cancer, Toronto, ON, Canada

 

## 1. Background/Rationale or Objectives/Purpose

Cancer survivors have psychosocial concerns after treatment, which impacts their ability to successfully return to life and work. Cancer survivors experience direct out-of-pocket and indirect costs associated with a cancer diagnosis, and 44% of those affected by cancer face substantial psychosocial burden, including distress, pain, and other negative experiences, which can lead to poorer health, clinical, and economic outcomes. Adolescent and young adults (AYAs) affected by cancer tend to be at great risk, given their vulnerable and highly dynamic life stage. Tools and resources for primary healthcare providers (PCPs) to support culturally safe, equity-focused patient-centric care for AYAs are needed.

## 2. Methodology or Methods

AYA cancer survivors (N = 50) were asked what they wished they had of asked their PCP after treatment but did not. The purpose was to hear directly from AYAs about their post-treatment experiences and needs, to inform the development of tools for PCPs. Qualitative content analysis was conducted, and two tools were developed to increase provider awareness and act as an aid to support the psychosocial needs of AYA cancer survivors.

## 3. Impact on Practice or Results

The psychosocial burden of cancer is substantial, particularly on those who are at greatest risk of being underserved. PCPs are empowered by patient-centric tools that help them support the unique psychosocial needs of AYA cancer survivors.

## 4. Discussion or Conclusions

More support is needed to address the psychosocial burden faced by AYA cancer patients, survivors, and their families, and new innovative approaches are required to empower PCPs to ensure the psychosocial needs of their patients no longer go unmet.

## 

**44 A Systematic Review of Mindfulness-Based Interventions for Psychological Well-Being and Quality of Life in Men with Prostate Cancer**

## 

NnateDaniel A.^1^AnyachukwuCanice C.^2^IgweSylvester E.^2^AbaraoguUkachukwu O.^3^1Department of Nursing and Community Health, School of Health and Life Sciences, Glasgow Caledonian University, Glasgow, UK2Department of Medical Rehabilitation, College of Medicine, University of Nigeria, Enugu, Nigeria3Department of Physiotherapy and Paramedicine, School of Health and Life Sciences, Glasgow Caledonian University, Glasgow, UK

 

## 1. Background/Rationale or Objectives/Purpose

Mindfulness-based interventions (MBIs) are increasingly being encouraged for managing treatment-related symptoms and psychological burden of cancer. Currently, much less is known about the extent to which mindfulness is effective in relieving the psychosocial distress experienced by men with prostate cancer.

## 2. Methodology or Methods

Quantitative literature search on Embase, CINAHL, MEDLINE, PsycINFO, PsycArticles, and WoS was conducted. Participants included men over 40 years who had a diagnosis of prostate cancer. Outcomes considered were psychological distress (anxiety, depression, cancer-specific distress), quality of life, and post-traumatic growth. The identified literature was assessed for quality using appropriate appraisal tools. Similar study results were pooled in meta-analyses, while narrative synthesis was used to summarize the findings from heterogeneous results, and statistical significance *p* ≤ 0.05.

## 3. Impact on Practice or Results

Four studies (three RCTs and one non-randomized study) met the inclusion criteria. MBIs for men with prostate cancer showed small to moderate effect for improving psychological outcomes. The pooled result of quality of life and post-traumatic growth in study participants showed moderate (d = −0.29 (−1.29, 0.71), *p* = 0.57) and large (d = 0.77 (−0.33, 1.88), *p* = 0.00) effects, respectively. The queries raised in our study are those concerned with the impact of comorbidity, sufficiency of research evidence, and stability of the overall effect estimate with the addition of new studies.

## 4. Discussion or Conclusions

Solid recommendations on mindfulness intervention for prostate cancer cannot be made based on the findings of this review due to limited research and inadequate methodological rigor of the published literature. Robust research is needed to draw a reliable conclusion about its sustained effect in men with advanced disease.

## 

**45 Emotion Regulation Patterns among Colorectal Cancer Survivors: Clustering and Associations with Personal Coping Resources**

## 

BazilianskySvetlana^1^,^2^CohenMiri^1^BenyAlexander^3^1University of Haifa, Haifa, Israel2RAMBAM Health Care Campus, Haifa, Israel3Assuta Hospital, Haifa, Israel

 

## 1. Background/Rationale or Objectives/Purpose

Emotion regulation patterns have a major impact on psychological adjustment and coping with challenges posed by cancer. Four main emotion regulation patterns are most commonly reported in the context of coping with cancer: repression, suppression, experiential avoidance, and cognitive reappraisal. The present study aimed to examine the relationships between these different emotion regulation patterns and their associations with two main personal resources: resilience and self-compassion.

## 2. Methodology or Methods

This cross-sectional study involved 153 colorectal cancer survivors with stages II–III of disease, 47% female, 53% male, aged 26–87, and 4–24 months since diagnosis who were consecutively recruited. During a follow-up visit at the oncology institute, participants completed questionnaires measuring levels of emotion regulation patterns, personal resilience, and self-compassion.

## 3. Impact on Practice or Results

A two-step cluster analysis identified three distinct subgroups of participants according to intensity of use of the four emotion regulation patterns clusters: Cluster 1, the suppression–avoidance dominant cluster; Cluster 2, the cognitive reappraisal dominant cluster; and Cluster 3, the repression dominant cluster. Personal resilience and self-compassion were higher in participants classified in Cluster 2 than the other clusters. In addition, Cluster 1 was characterized by a longer time since diagnosis and Cluster 2 was characterized by more male participants and higher levels of education and economic status.

## 4. Discussion or Conclusions

Healthcare professionals should be aware and identify the emotion regulation patterns used by cancer survivors and to strengthen the use of the more adaptive patterns.

## 

**46 A Qualitative Study of Women** **’s Experiences and Concerns of the Perinatal Period after Cancer**

## 

VanstoneRuth^1^FergusKaren^1^LadhaniNoor Niyar N.^2^WarnerEllen^2^1York University, Toronto, ON, Canada2Sunnybrook Health Sciences Centre, Toronto, ON, Canada

 

## 1. Background/Rationale or Objectives/Purpose

The present investigation sought to better understand women’s experiences of pregnancy and the postpartum period following cancer treatment through a qualitative analysis.

## 2. Methodology or Methods

Ten women participated in a semi-structured, one-on-one interview either over telephone or video conferencing (Zoom). Participants all had a past cancer diagnosis; no active disease; were 45 years of age or younger; currently in the perinatal period; and were able to speak English fluently. The study employed a grounded theory analysis by which verbatim interview (textual) data were analyzed using a constant comparison method until data saturation was reached.

## 3. Impact on Practice or Results

A grounded theory analysis yielded *I’m So Happy, But Also Terrified*, as the core category, which was indicative of the duality of emotional experience that characterized the perinatal period for these women. Additionally, four constitutive higher-order categories revealed how women go through a process of (1) grief related to potential fertility loss; (2) conditional joy during and after pregnancy due to the lingering weight of cancer; (3) frustration with a lack of resources regarding perinatal health after cancer; and (4) hope as they enter into motherhood.

## 4. Discussion or Conclusions

These results suggest that women in the perinatal period with a history of cancer may be at an increased risk for psychological distress and require additional fertility and reproductive resources both during and after cancer treatment. This research is an important step in further understanding women’s experiences of pregnancy after cancer and may help to inform future research and healthcare practices, in addition to improving perinatal care after cancer. 

## 

**47 Pain and Sensory Abnormalities in Survivors of Childhood Cancer: A Quantitative Sensory Testing Study**

## 

TutelmanPerri^1^,^2^ChambersChristine^1^,^2^CornelissenLaura^3^,^4^FernandezConrad^1^,^2^FlandersAnnette^2^HeathcoteLauren^5^SchulteFiona^6^,^7^MacLeodJulia^8^SternMaya^9^TeamThe Pain in Childhood Cancer Survivors^1^1Dalhousie University, Halifax, NS, Canada2IWK Health Centre, Halifax, NS, Canada3Boston Children’s Hospital, Boston, MA, USA4Harvard Medical School, Boston, MA, USA5Stanford University School of Medicine, Stanford, CA, USA6University of Calgary, Calgary, AB, Canada7Alberta Children’s Hospital, Calgary, AB, Canada8Patient Partner, Halifax, NS, Canada9Patient Partner, Toronto, ON, Canada

 

## 1. Background/Rationale or Objectives/Purpose

Childhood cancer survivors are at risk for long-term challenges such as pain. Mechanisms of pain persistence in this population are unclear but may be due to alterations in central and peripheral sensory processing. Quantitative sensory testing (QST) was used to examine patterns of pain and sensory processing compared to age- and sex-matched normative data.

## 2. Methodology or Methods

Participants were 57 survivors of childhood cancer (52% male, *M_age_* = 13.5, *SD* = 3.2, range = 8–17). The most common diagnoses were acute lymphoblastic leukemia (43%) and Wilms tumor (13%). On average, children were 7.1 years (*SD* = 4.1, range = 1.2–16.5) post-treatment. A standardized QST protocol assessed sensory detection and pain thresholds at the right thenar eminence. 

## 3. Impact on Practice or Results

Almost all survivors (86%) had at least one abnormal QST parameter. Overall, loss of function was found for cold detection *t*(108) = 5.08, *p* < 0.001, warm detection *t*(108) = 3.21, *p* < 0.01, cold pain *t*(108) = 3.45, *p* < 0.001, and mechanical pain *t*(108) = 6.70, *p* < 0.001 thresholds compared to normative data. Gain of function was found for mechanical pain sensitivity *t*(92) = 7.60, *p* < 0.001. Ten survivors (19%) exhibited mechanical allodynia. History of leukemia was associated with less sensitivity to cold *r*(108) = −0.31, *p* < 0.05 and greater sensitivity to mechanical *r*(108) = −0.29, *p* < 0.05 pain. Children with higher levels of pain catastrophizing and anxiety displayed greater sensitivity to cold *r*(108) = 0.38, *p* < 0.01 and mechanical *r*(108) = 0.27, *p* < 0.05 pain, respectively.

## 4. Discussion or Conclusions

QST revealed decreased sensory detection and increased pain sensitization in childhood cancer survivors compared to healthy children, which may be the result of their illness, treatment exposures, and/or social–environmental factors. Certain survivors may be at increased risk based on their clinical (e.g., leukemia) and psychological (e.g., catastrophizing, anxiety) profiles. 

## 

**62 Cancer Survivorship, Positivity, Meaning-Making, and Post-Traumatic Growth: A Qualitative Investigation into the Post-Treatment Juncture**

## 

HermanDavid^1^,^2^FergusKaren^1^,^2^IanakievaIana^1^,^2^GandhiManisha^2^LeaheyAngela^2^1York University, Toronto, ON, Canada2Odette Cancer Centre, Sunnybrook Health Sciences Centre, Toronto, ON, Canada

 

## 1. Background/Rationale or Objectives/Purpose

In addition to the significant losses and distress associated with having had cancer, a survivor’s illness experience may also provide an inimitable opportunity to experience personal growth and discover what is most meaningful and significant in one’s life. This study investigated the positive value that cancer survivors derive from their illness experience during the first six months after completing primary treatment. One’s experience during this time is critical, as it often sets the stage for the rest of his–her post-cancer journey. 

## 2. Methodology or Methods

We sought to develop a qualitative, inductive, and comprehensive understanding of how positive value is construed by cancer survivors. Six patients, ranging in age from 51 to 78, who had recently completed treatment for a primary cancer (breast, lymphoma, urinary, skin, and rectal) participated in a narrative-care based interview. Interviews were transcribed verbatim and, using a grounded theory analysis of the transcripts, we constructed a five-tier hierarchical framework for understanding how individuals draw benefit from the challenging experience of cancer. 

## 3. Impact on Practice or Results

Our analysis yielded five main categories forming the core category ‘Reclaiming Oneself and Discovering Others Despite the Losses’: (1) *Accepting What’s Beyond My Control*, (2) *Discovering What’s Most Important*, (3) *Cultivating a Positive Perspective*, (4) *Web of Social Connectedness*, and (5) *Becoming More Agentic*. These categories elucidate the key constituents of positive value as described by our sample.

## 4. Discussion or Conclusions

Our findings contribute to the growing literature on post-traumatic growth and meaning-focused coping and can be used to inform researchers/clinicians who adopt a strengths-based, narrative-reframing approach to treating cancer patients.

## 

**68 The Association between Survivorship Care Plans and Patient-Reported Confidence with Follow-Up Cancer Care Provided by Primary Care Providers**

## 

ChuAlannaMutsaersBrittanyLebelSophieUniversity of Ottawa, Ottawa, ON, Canada

 

## 1. Background/Rationale or Objectives/Purpose

Survivorship Care Plans (SCPs) aim to improve the continuity of follow-up care for a growing number of cancer survivors transitioning from hospital to primary care providers (PCPs). However, evidence of the efficacy of SCPs is mixed. This study seeks to identify sociodemographic factors (SDFs) associated with receiving a SCP and examine the relationship between SCPs and confidence in follow-up care delivered by PCPs.

## 2. Methodology or Methods

A cross-sectional retrospective analysis of the Canadian Partnership Against Cancer’s *Experiences of Cancer Patients in Transition Study* was conducted (n = 9970). An adjusted logistic regression model examined the association between SDFs and odds of receiving a SCP. Separate adjusted multinomial logistic regression models assessed the relationship between SCPs and PCP care outcomes. Separate bivariate analyses (chi-squared) were conducted for each relationship. 

## 3. Impact on Practice or Results

In an adjusted model that contained all SDFs, odds of receiving an SCP were significantly increased for males, high school or less education, and unpaid employment and decreased for graduate education and unsure metastatic status. Adjusting for all SDFs, individuals who received an SCP had significantly higher odds of the following: having a general practitioner (GP) or oncologist and a GP responsible for follow-up care compared to an oncologist alone, having felt their PCPs were involved, and agreeing that their PCPs understood their needs, knew where to find supports and services, were able to refer them directly to services, and were confident that their PCPs could meet their follow-up care needs. 

## 4. Discussion or Conclusions

Results support the provision of SCPs to survivors to improve confidence in follow-up care provided by PCPs.

## 

**72 Supporting the Needs of Adolescent and Young Adult Cancer Survivors: Identifying the Gaps in Post-Treatment Care**

## 

ChapmanStacy^1^,^2^EmanueleCristina^3^VoraTushar^2^GuptaAbha^2^,^3^AveryJonathan^2^,^4^1University of Manitoba, Winnipeg, MB, Canada2Princess Margaret Cancer Centre, Toronto, ON, Canada3The Hospital for Sick Children (SickKids), Toronto, ON, Canada4University of British Columbia, Vancouver, BC, Canada

 

## 1. Background/Rationale or Objectives/Purpose

Adolescent and young adults (AYA), defined as individuals between 15 and 39 years of age, who have completed their cancer treatment face increased risk of secondary malignancies, decreased fertility, socioeconomic hardships, and difficulties coping. Despite its importance, post-treatment care is lacking for this population. To determine the level of post-treatment care currently being provided and to identify areas for improvement, we conducted two virtual/online focus groups with AYA diagnosed with cancer. 

## 2. Methodology or Methods

Focus groups were conducted using the Microsoft Teams virtual platform. AYA participants were recruited from outpatient disease site clinics at the Princess Margaret Cancer Centre (UHN, Toronto). A discussion guide was used to navigate different areas of post-treatment care. Recordings were transcribed verbatim and analyzed using thematic analysis. 

## 3. Impact on Practice or Results

A total of 10 AYAs participated in focus groups. The participants’ ages ranged from 21 to 43 years old. We included individuals older than 39 who were diagnosed when they were younger. Findings indicate an overall lack of life stage-focused support and information to address the consequences from cancer treatments. Three themes describe suggestions for improvement: (1) having peer-driven support; (2) incorporating a hybrid of online and in-person programming; and (3) finding ways to integrate general practitioners (GPs).

## 4. Discussion or Conclusions

Findings suggest the need for a hybrid model of online and in-person support created and delivered by AYA that would also involve ways to integrate GPs. A partnership with the Canadian Cancer Society has been formed to address this need on a national scale.

## 

**75 A Silent Epidemic of Depression among Prostate Cancer Survivors**

## 

IlieGabrielaDalhousie University, Halifax, NS, Canada

 

## 1. Background/Rationale or Objectives/Purpose

Prostate and skin cancer are among the most prevalent forms of cancer among men and have favorable survival rates compared to other more aggressive forms of cancers. Recent studies have shown that among men with a lifetime history of prostate cancer, the prevalence of depression is higher compared to men without a history of prostate cancer. Here, we extend previous findings and examine the role of socioeconomic status in this relationship in a population-based sample of men.

## 2. Methodology or Methods

The analytic sample included 6585 male participants aged 49–69 who completed a lifestyle survey within the 2009–2015 surveillance cycle of the Atlantic PATH study. The primary outcome was screening positive for mild, moderate, or severe depression using the Patient Health Questionnaire (PHQ-9). The main predictor variable was cancer survivorship status (the presence of a lifetime history of prostate, skin, other forms of cancer, or absence of a lifetime cancer diagnosis). Covariates included age, education, marital status, household income, province, ethnicity, comorbidity, and survivorship time.

## 3. Impact on Practice or Results

Almost 15% of men in this sample screened positive for mild, moderate, or severe depression. The odds of screening positive for depression were 2.60 times higher for survivors of prostate cancer than men with a history of any other form of cancer. Odds ratios were 10.23 or 4.00 times higher for survivors of prostate or skin cancer who also reported low household income to screen positive for depression compared to their counterparts.

## 4. Discussion or Conclusions

Results corroborate and extend recent studies pointing to a silent epidemic of depression among prostate cancer survivors and show that this association is moderated by household income. Delivering mental health screening and support to cancer survivors during the cancer journey, especially those with low household incomes, is warranted.

## 

**87 Primary Care Providers Needs Assessment in Supporting Cancer Survivors with Return to Work**

## 

MaheuChristine^1^,^2^ParkinsonMaureen^3^FlanaganTanya^4^1McGill University, Montreal, QC, Canada2McGill University Health Centre, Montreal, QC, Canada3BC Cancer, Vancouver, BC, Canada4Canadian Partnership Against Cancer, Toronto, ON, Canada

 

## 1. Background/Rationale or Objectives/Purpose

**Objectives/purpose:** Primary care providers ((PCP); Family Physicians and Nurse Practitioners) play a crucial role in supporting the return to work (RTW) of cancer survivors. However, they note the lack of knowledge and skills to advise on cancer’s impact on work-related topics as a major barrier. To fill this gap, we are developing and implementing an e-course for PCP on return to work of cancer survivors.

## 2. Methodology or Methods

**Sample and setting:** The team composed of vocational and rehabilitation specialists invited an advisory group composed of PCPs, specialists in oncology and psychiatry, and cancer survivors to inform the development and implementation of the e-course. **Procedures:** As part of education curriculum development guidelines, a needs assessment survey was conducted with PCP to identify needs, barriers, and utilization of resources and information to help them support survivors with RTW.

## 3. Impact on Practice or Results

**Results**: Fifty PCPs responded to the survey and identified the following needs for the e-course content: the need for knowledge and communication about treatment, follow-ups, and effects of cancer over time; treatment guidance to manage symptoms and psychological concerns; knowledge of vocational rehabilitation; better understanding of workplace demands; how to assess fitness to return to work; and knowledge of resources to assist with RTW of survivors. The Canadian bilingual Cancer and Work website (www.cancerandwork.ca) was identified as a helpful resource to them in supporting RTW.

## 4. Discussion or Conclusions

**Conclusion and Clinical Implications:** The e-curriculum for PCP (funded by the Canadian Partnership Cancer) to be developed and implemented for summer 2021 will be freely available and will fill the gap in the lack of training and resources in this area and will provide preliminary data on its utility to increase PCP perceived self-efficacy to support the RTW of cancer survivors. 

## 

**92 The Chemo-Gut Study: Pilot Data Exploring Health Behaviors, Quality of Life, and Gastrointestinal Health in Cancer Survivors**

## 

DeleemansJulie M.ReimerRaylene A.CarlsonLinda E.University of Calgary Cumming School of Medicine, Calgary, AB, Canada

 

## 1. Background/Rationale or Objectives/Purpose

Chemotherapy adversely affects the gut microbiota, which may contribute to persistent gastrointestinal (GI) and mental health issues. We assessed survivor’s health behaviors, quality of life (QoL), and GI health, and we investigate relationships between chemotherapy, GI, and QoL outcomes.

## 2. Methodology or Methods

A locally designed patient-reported outcome tool and Global Health PROMIS measure were used. Cancer survivors (N = 292) aged 18 years or older, living in Canada who completed anti-cancer therapies were included. Descriptive statistics, frequencies, and one-way ANOVA’s explored health behaviors, QoL, and GI issues.

## 3. Impact on Practice or Results

Mean current age was 47.4 years (SD = 14.8); 82% were female. Mean age at diagnosis was 41.6 years (SD = 15.1). The most common diagnosis was breast cancer (43%). Most survivors received chemotherapy (84%). In the last 2 years, 45% used antibiotics, and 34% used probiotics. Nearly two-thirds (66%) reported engaging in physical activity for 5 or fewer hours per week, and 55% rated their diet as moderately healthy. Persistent GI issues include constipation (54%), diarrhea (51%), bloating, and pain (53%). Mean GI issue duration was 30 months (SD = 31.7), with 75% of survivors experiencing GI issues for up to 48 months post-treatment. The severity of GI symptom interference was moderate to high for 43% of survivors. Chemotherapy was significantly associated with constipation, diarrhea, bloating and pain (*p’s* < 0.001), and nausea (*p* = 0.019). Physical health was “fair” (M = 42.6, SD = 8.2). Mental health was “good” (M = 45.3, SD = 8.0). GI interference was significantly associated with mental and physical (*p’s* < 0.001) health outcomes, as greater interference was associated with poorer outcomes.

## 4. Discussion or Conclusions

Three-quarters of cancer survivors experienced persistent GI issues for up to 48 months post-treatment. Chemotherapy was associated with more GI issues, and greater GI symptom interference was associated with poorer physical and mental health.

## 

**106 Returning to Work Following Cancer: Strategies, Insights, and Reflective Exercises to Get You Moving Forward**

## 

LadouceurMelinaBarrett-RobillardPatriciaWoodardStephanieOttawa Regional Cancer Foundation, Ottawa, ON, Canada

 

## 1. Background/Rationale or Objectives/Purpose

Both the Canadian Partnership Against Cancer and Young Adult Cancer Canada report that returning to work is one of the biggest concerns for patients post-treatment. Our presentation, aimed at both patients and professionals, shines a light on key learnings from 10 years of delivering a return to work program to our clients. In our presentation, we cover topics such as coping with side effects that can affect work life, handling questions from colleagues, as well as ways to foster more feelings of inclusion and understanding in the workplace. We will also focus on learning from our differences by having a client share their story on work following cancer. Lastly, we will explore how a reflective exercise we have given our clients helped them in uncovering what was key for them in life and work moving forward.

## 2. Methodology or Methods

Our “Rebalancing Life and Work After Cancer” program supports patients in figuring out the answer to the question: “What now?” Through this multi-week program, we provide education, allow opportunities for discussion, creative problem solving, and use health and life coaching techniques to help our clients in developing a plan on how they can successfully return to work. 

## 3. Impact on Practice or Results

Participants who complete our program tend to return to work feeling more empowered and confident. This is a direct result of the development of their personal plan, which revolves around a better understanding of how to manage side effects and planning in advance for how they will handle common challenges when returning to work. 

## 4. Discussion or Conclusions

We plan to continue to offer coaching, which has proven to assist patients in navigating this challenging transition. Future ambitions are to also develop programs and partnerships with employers and insurance companies.

## 

**109 Psychiatric Disorder Incidence among Adolescents and Young Adults Aged 15–39 with Cancer: A Population-Based Cohort Study**

## 

CherakStephana^1^FiestKirsten^1^McKillopSarah^2^DiazRuth^3^BarrRonald^4^PattenScott^1^RosgenBrianna^1^DeleemansJulie^1^Fidler-BenaoudiaMiranda^1^1University of Calgary, Calgary, AB, Canada2University of Alberta, Edmonton, AB, Canada3Alberta Health Services, Calgary, AB, Canada4McMaster University, Hamilton, ON, Canada

 

## 1. Background/Rationale or Objectives/Purpose

Adolescent and young adult (AYA) cancer survivors have distinct patterns of cancer-associated late effects. It is unclear if AYA cancer survivors are at increased risk for psychiatric disorders.

## 2. Methodology or Methods

We performed a population-based cohort study of five-year survivors of cancer who were diagnosed at 15–39 years of age during the period 1991 to 2013. The incidence of seven psychiatric disorders (i.e., anxiety, depressive, severe psychiatric, trauma-and-stressor-related, substance use, psychotic including bipolar, and suicide and self-harm attempts) were assessed three years after the first cancer diagnosis. Demographic and cancer- and treatment-related variables were explored as risk factors for psychiatric disorders. 

## 3. Impact on Practice or Results

Among 12,116 five-year AYA cancer survivors (*n* = 4634 (38%) males; *n* = 7482 (62%) females), 9602 (79%; *n* = 4634 (48%) males; *n* = 7482 (78%) females) were diagnosed with at least one of seven psychiatric disorders after three years post-cancer diagnosis. Survivors of all cancers were most often diagnosed with anxiety (males, 55.7%, 95% confidence interval (CI) 54.2–57.1; females, 73.8%, 95%CI 72.8–74.8), depressive (males, 44.7%, 95%CI 43.4–46.3; females, 62.6%, 95%CI 61.5–63.7), and severe psychiatric disorders (males, 38.0%, 95%CI 36.6–39.5; females, 54.2%, 95%CI 53.1–55.4). Compared to females, males were diagnosed more frequently with substance use disorders (14.3%, 95%CI 13.3–15.3 versus females 9.5%, 95%CI 8.8–10.1), whereas females of all ages more frequently experienced trauma- and stressor-related disorders 34.8%, 95%CI 33.7–35.8 versus males 21.7%, 95%CI 20.5–22.9). Substance use disorders, psychotic disorders, and suicide attempts/self-harm were most common among AYA cancer survivors aged 15–19 years at first cancer diagnosis.

## 4. Discussion or Conclusions

Anxiety disorders, depressive disorders, and severe psychiatric disorders are common amongst five-year survivors of AYA cancer. Preventive strategies for AYAs diagnosed with cancer, particularly at an early age, are needed to mitigate the risk of severe and potentially fatal outcomes due to long-term psychiatric disorders. 

## 

**111 Factors Associated with Orthorexia Symptoms and Disordered Eating Behaviors in Young Women with Cancer**

## 

WatermanMeghanGarlandSheilaMemorial University of Newfoundland, St. John’s, NL, Canada

 

## 1. Background/Rationale or Objectives/Purpose

A cancer diagnosis can motivate people to modify lifestyle behaviors that they believe could influence their prognosis or recurrence risk. Orthorexia (ON) is a disordered eating behavior (DEB) that involves a fixation on health-conscious eating behaviors. This research explored the presence of ON symptoms in a sample of young adult (YA) females with cancer and explored what factors may be associated with ON and DEB symptom severity.

## 2. Methodology or Methods

Ninety-three YA females with cancer between the ages of 19 and 39 participated in an online survey. The Düsseldorf Orthorexia Scale and the Eating Habits Questionnaire measured ON severity and DEBs. Linear regressions were used to identify associations between ON severity, DEBs, fear of cancer recurrence, body image dissatisfaction, intolerance of uncertainty, and internet use.

## 3. Impact on Practice or Results

The mean age was 31 with an average of 17 years of education. Overall, 36.7% of participants reported clinically significant ON symptoms and 20.4% were at risk for ON. More intolerance of uncertainty was related to increased ON severity (β = 211, *p* = 0.05). Greater fear of recurrence was associated with more problems resulting from healthy eating (β = 213, *p* = 0.05). Less time spent online for personal reasons was related to more positive feelings associated with healthy eating (β = −0.206, *p* = 0.05). Greater dissatisfaction with body image was related to more perceived knowledge of healthy eating (β = −0.236, *p* = 0.03).

## 4. Discussion or Conclusions

YA females with cancer are showing symptoms of ON that are associated with potentially modifiable psychological factors.

## 

**112 Changes in Sedentary Time and Associations with Quality of Life in Cancer Survivors during the COVID-19 Pandemic**

## 

NevilleAlyssaTabaczynskiAllysonWhitehornAlexisBastasDeniseTrinhLindaUniversity of Toronto, Faculty of Kinesiology and Physical Education, Toronto, ON, Canada

 

## 1. Background/Rationale or Objectives/Purpose

The impact of sedentary time (SED) on quality of life (QoL) in cancer survivors during the COVID-19 pandemic remains unknown. The purpose of this study was to compare total and domain-specific SED before and during the pandemic and examine associations on QoL in a global sample of cancer survivors. 

## 2. Methodology or Methods

In an online survey, cancer survivors self-reported SED for transportation, work, television, computer, and other leisure activities before and during the pandemic using the Domain-Specific Sitting Time Questionnaire. QoL was assessed via the Functional Assessment of Cancer Therapy (FACT)-General, FACT-Fatigue, and Trial Outcome Index-Fatigue scales. Paired *t*-tests compared SED before and during the pandemic. Analysis of covariance compared QoL between survivors who remained high (≥8 h/day), remained low (<8 h/day), increased (<8 h/day to ≥8 h/day), or decreased (≥8 h/day to <8 h/day) SED.

## 3. Impact on Practice or Results

Cancer survivors (N = 505; M_age_ = 48.2 ± 15.4) were primarily females (69.5%) and diagnosed with breast cancer (28.3%). Among survivors, 60.2% remained high, 20% remained low, 7.5% increased SED, and 12.3% decreased SED. Despite no differences in total daily SED before and during the pandemic, screen time (e.g., television, computer) significantly increased (*p’s* < 0.001), but SED during transportation, work, and other leisure activities decreased (*p’s* < 0.02). Subgroup analyses revealed that those with <3 comorbidities achieving low SED had significantly better QoL on the FACT-General and FACT-Fatigue, whose scores were clinically meaningful compared to those who increased SED (*p’s* < 0.007). 

## 4. Discussion or Conclusions

Survivors did not change total daily SED during the pandemic but increased screen time. Increasing SED can negatively impact QoL for those with comorbidities. Behavioral strategies for cancer survivors should focus on reducing SED to improve QoL outcomes with an emphasis on reducing screen time.

## 

*Abstract Theme: Palliative and End-Of-Life Care*

**4 Reflecting on Palliative Care Integration in Healthcare across Canada: A Brief Qualitative Report**

## 

QureshiMaryam^1^RobinsonMaggie^2^SinnarajahAynharan^3^,^4^CharySrini^3^,^4^GrootJanet De^2^,^3^FeldstainAndrea^2^,^3^1Werklund School of Education, University of Calgary, Calgary, AB, Canada2Department of Psychosocial and Rehabilitation Oncology, Alberta Health Services, Calgary, AB, Canada3Department of Oncology Cumming School of Medicine, Calgary, AB, Canada4Palliative and End of Life Care, Alberta Health Services, Calgary, AB, Canada

 

## 1. Background/Rationale or Objectives/Purpose

Past studies have identified ‘interdisciplinary integrated care’ as a hallmark of effective palliative care. Although palliative care models attempt to show how integration functions, little literature practically explores how integration is fostered and maintained. In this study, we asked palliative care clinicians across Canada to comment on how palliative is integrated with medical services in their region. Our objective was to map the current state of palliative care integration.

## 2. Methodology or Methods

This is an analysis of data from a larger study, wherein clinicians provided written responses to questions evaluating their experience of palliative care in Canada. We contacted directors of Palliative Care programs across Canada and used snowball sampling to reach any clinician working in palliative care. We used a qualitative content analysis approach to identify categories in clinician responses.

## 3. Impact on Practice or Results

Clinicians identified the need for formalized relationships and collaboration pathways with other services, to streamline referral and consultation. Clinicians also identified the need to better educate/train family physicians in the community, which would lead to more buy-in, shared understandings, consultation, and referral. Clinicians described integrating well with oncology. Lastly, clinicians considered integration as a complex process with departmental, provincial, and national involvement.

## 4. Discussion or Conclusions

The needs identified by our clinicians mirror qualities of successfully integrated palliative care programs. Canada has been identified as a leader in palliative care, and our results highlight areas in policy, education, practice, and research that could further benefit our programs. Lastly, we highlight how integration is nuanced, contextual, and multi-tiered, requiring individualized solutions for each region. 

## 

**8 The Development and Validation of a Patient-Reported Compassion Measure for Research and Practice: The Sinclair Compassion Questionnaire (SCQ)**

## 

SinclairShane^1^HackThomas^2^MacInnisCara^1^JaggiPriya^1^BossHarrison^1^McClementSusan^3^SinnarajahAynharan^1^ThompsonGenevieve^3^1University of Calgary, Calgary, AB, Canada2University of Manitoba, Winnipeg, MB, Canada3University of Manitoba, Winnipeg, MB, Canada

 

## 1. Background/Rationale or Objectives/Purpose

To present the results of a nationally funded multi-center study to develop and validate a patient-reported experience measure of compassion.

## 2. Methodology or Methods

Participants (n = 633) living with advanced cancer and other incurable, life-limiting illnesses were recruited from four care settings (acute care, hospice, long-term care (LTC), and home care) in Calgary and Winnipeg. 

Candidate items were first generated from each of the domains of the Patient Compassion Model. After conducting a modified Delphi approach garnering feedback from international subject matter experts and patient advisors, cognitive interviews with patients, 54 candidate items were administered to 303 patients in the Exploratory Factor Analysis (EFA) phase, with 65 patients repeating the measure one day later, to assess test–retest reliability. In the Confirmatory Factor Analysis (CFA) phase of the study, the final 15-item measure was administered to 330 patients along with other measures to establish convergent and divergent validity.

## 3. Impact on Practice or Results

EFA and CFA confirmed that the 15-item SCQ loads on a single factor—compassion—with factor loadings for the 15 items ranging between 0.76 and 0.86, test–retest reliability (0.74–0.89), and internal reliability (Cronbach’s alpha 0.961). The SCQ was positively correlated with the SCCCS (r = 0.75, *p* < 0.001) and PPEQ (r = 0.60, *p* < 0.001). Patients reporting higher experiences of compassion had significantly greater well-being and lower depression (ESAS-r).

## 4. Discussion or Conclusions

The SCQ is a valid and reliable patient-reported compassion measure, providing researchers with a ‘gold standard’ to conduct high-quality compassion research while providing healthcare providers with a clinical tool for routinely assessing compassion.

## 

**65 Advance Care Planning Education Module: A Video Module for Healthcare Providers to Increase Their Knowledge of Advance Care Planning**

## 

TysonEmilyMcknightErinHorstTeddiPalliative Care Resource Team, Health and Social Services, Whitehorse, YT, Canada

 

## 1. Background/Rationale or Objectives/Purpose

This video module was created by the Palliative Care Resource Team for Health Care Providers (HCPs) to increase their knowledge of Advance Care Planning (ACP): what it is and is not, why it is important, and when it is appropriate to initiate ACP conversations. This module is intended to increase HCP comfort and confidence in having ACP conversations with clients by reviewing the five steps of ACP (Think, Learn, Decide, Talk, Record). This module reflects how these conversations may happen in a rural and remote setting (Yukon).

## 2. Methodology or Methods

This video module is intended to be shared with HCPs throughout the Yukon territory and across all care settings. The module features local HCPs including a hospital social worker, Palliative Care Physician, Community Liaison Coordinator, and Palliative Care RN as they follow “Joe’s” story throughout his illness trajectory. This module highlights the various opportunities along “Joe’s” journey for ACP conversations. This module emphasizes the importance of ACP conversations, how the conversations fit into a palliative approach to care as well as to clarify the difference between ACP and Goals of Care conversations.

## 3. Impact on Practice or Results

This module is currently available and online can be accessed by all HCPs via the Yukon Government’s online education platform “YG Learn”.

## 4. Discussion or Conclusions

The Palliative Care Resource Team will host a virtual viewing of this module on ACP Day (April 16) followed by a Q and A session with the team as well as a Palliative Care Physician who collaborated with the team in the development of the module. A survey will be sent out afterwards to all participants to assess if the learning objectives of the module were achieved.

## 

**73 A Longitudinal Study of Medical Assistance in Dying (MAiD) in Patients with Advanced Cancer and their Caregivers**

## 

LiMadelineShapiroGilla K.RydallAnneBarbeauAnneKleinRoberta Y.FitzgibbonKylePanjwaniAlizaZimmermannCamillaWongRebeccaRodinGaryPrincess Margaret Cancer Centre, Toronto, ON, Canada

 

## 1. Background/Rationale or Objectives/Purpose

Little is known about the emergence of the desire for death (DD) in patients, its impact on caregivers, and to what extent supportive care interventions affect DD and requests for Medical Assistance in Dying (MAiD). This longitudinal study is designed to determine the prevalence, predictors, and experience of DD and MAiD in patients with advanced cancer, and the impact on their primary caregivers.

## 2. Methodology or Methods

***Sample and Setting**:* In this CIHR-funded 5 year study, a cohort of 600 patients with advanced cancer and their primary caregivers will be recruited at the Princess Margaret Cancer Centre to a mixed methods study.

***Procedures**:* Subjects will be assessed at baseline and every 6 months for diagnostic information, sociodemographic and psychological characteristics, medical status, quality of life, physical and psychological distress, attitudes about the DD and MAiD, communication with physicians, advance care planning, and use of psychosocial and palliative care interventions. Caregivers will additionally be assessed for relationship quality, caregiver experience, and at 6 months post-patient death, quality of death, depression, traumatic stress, and grief. Qualitative interviews will be conducted in a subset of both patients and caregivers selected using quota sampling methods.

## 3. Impact on Practice or Results

Preliminary results not yet available. Feedback is sought on (1) strategies to decrease questionnaire burden in study subjects, (2) study acceptability to patients, and (3) additional variables of interest.

## 4. Discussion or Conclusions

The findings from this study may assist healthcare providers in their initial conversations with patients and caregivers about MAiD and guide changes being considered to broaden MAiD legislation and policy.

## 

**78 Applying the Social Cognitive Processing Theory to Advanced Care Planning and Medical Assistance in Dying among Patients with Incurable Cancers**

## 

PanjwaniAliza A.KleinRoberta Y.RydallAnneShapiroGilla K.FitzgibbonKyleBarbeauAnneRodinGaryLiMadelinePrincess Margaret Cancer Centre, Toronto, ON, Canada

 

## 1. Background/Rationale or Objectives/Purpose

In Canada, Medical Assistance in Dying (MAiD) is not yet allowed as part of advance care planning (ACP). Understanding psychosocial and behavioral factors underlying ACP and MAiD may inform proposed changes to upcoming legislation. The Social Cognitive Processing (SCP) theory posits that intrapersonal factors (e.g., intolerance of uncertainty (IU), cognitive inflexibility (CI), experiential avoidance (EA)) interact with interpersonal factors (e.g., relationships with family/friends and physicians) to influence adjustment to illness. Accordingly, we will examine and compare the interaction of intrapersonal and intrapersonal factors on ACP and MAiD requests/attitudes.

## 2. Methodology or Methods

***Sample and setting**:* This proposed sub-study of a larger mixed-methods longitudinal study of MAiD in advanced cancer patients at Princess Margaret Cancer Centre will recruit 120 participants.

***Procedures**:* Participants will complete measures of IU, CI, EA, and ACP engagement, attachment style, satisfaction with physician care and communication, attitudes toward MAiD, death anxiety, and depression. Data on demographic and medical variables, advance directives, and MAiD requests will also be collected. Structural equation modeling will test a moderated mediation model.

## 3. Impact on Practice or Results

Pending. Feedback on (1) theoretical and empirical relationship between IU and cognitive inflexibility; (2) inclusion of distress variables in the models.

## 4. Discussion or Conclusions

The proposed research will test an SCP framework in ACP and MAiD and identify targets for psychotherapeutic interventions to support planning for end of life. Findings may help determine whether the psychological processes underlying ACP and MAiD operate similarly or are distinct, so as to inform considerations on including MAiD as an advance directive.

## 

**93 Understanding Symptom Trajectories and Supportive Care Resource Utilization in Cancer Patients Who Completed Medical Assistance in Dying (MAID)**

## 

WatsonLinda^1^,^2^RussellBrooke^2^SchulteFiona^2^SilviusJim^1^KellyBrian^2^,^3^BultzBarry^2^,^3^1Alberta Health Services, Calgary, AB, Canada2University of Calgary, Calgary, AB, Canada3University of Newcastle, Newcastle, NSW, Australia

 

## 1. Background/Rationale or Objectives/Purpose

This study explores the relationship between Patient-Reported Outcome (PRO) data collected for ambulatory oncology patients who completed MAID and their use of psychosocial, rehabilitation, and pain clinic supportive care services.

## 2. Methodology or Methods

The study sample includes a total of 337 cancer patients who completed MAID between July 2017 and January 2019 in Alberta, Canada. Descriptive statistics were applied for sociodemographic and disease-specific data of the cohort. A mixed effect model (MEM) was used to examine the trajectory of PRO data and the use of supportive care services. Symptom complexity levels were modeled over time using the Generalized Estimating Equation (GEE). 

## 3. Impact on Practice or Results

Of the 337 cancer patients included in this cohort, 162 (48.1%) were women and 175 were men (51.9%). The mean age at MAID was 72.7 years (SD = 12.0, range = 26–98). The most common tumor group was gastrointestinal (25.5%), followed by lung (15.7%) and genitourinary (12.5%). More than half (N = 193, 57.3%) completed at least one PRO measure in the 12 months prior to MAID. A significant main effect of time was observed for symptom complexity levels and nine individual symptoms, indicating patients reported escalating symptoms as their MAID date approached. Supportive care utilization data points to an underutilization of supportive care resources in the last 12 months of life.

## 4. Discussion or Conclusions

This PRO and supportive care data illustrate potential areas for quality improvement related to symptom management and supportive care utilization for patients in the last 12 months of life. 

## 

*Abstract Theme: Primary, Secondary, and Tertiary Cancer Prevention*

**2 The Importance of Relationship Quality for Skin Self-Exam Support in Melanoma Follow-Up Care**

## 

CastelliSamanthaMackaySarahBergeronCatherineKornerAnnettMcGill, Montreal, QC, Canada

 

## 1. Background/Rationale or Objectives/Purpose

Patients with melanoma use a variety of cognitive and behavioral strategies to cope with the physical and psychological challenges of the disease, such as support seeking. Skin self-examination (SSE) is a means for the early detection of subsequent melanomas among individuals at increased risk and is considered more effective when done with support from others. Given that melanoma can occur anywhere on the body, patients may feel more comfortable seeking self-exam support from a romantic partner. However, little is known about how this kind of partner support is impacted by the quality of the relationship.

**Aim**: The present study examines whether relationship quality mediates the association between support seeking and perceived support for SSE. 

## 2. Methodology or Methods

We conducted a mediation analysis on a sample of 189 melanoma survivors (18y+) who completed measures of coping (Brief COPE), relationship quality (DAS), and perceived support for skin self-examination (SSE-support).

## 3. Impact on Practice or Results

Relationship quality accounted for the association between support seeking and partner support for SSE (before mediator β = 0.20, *p* < 0.05; after mediator β = 0.14, *p* = 0.13).

## 4. Discussion or Conclusions

Findings suggest that relationship quality plays an important role in melanoma survivors’ support seeking strategies. 

**Clinical implications:** Clinicians providing educational interventions on the early detection of melanoma through SSE may address how to effectively communicate support needs within an intimate relationship. It can also be beneficial to include the romantic partner in the education on how to examine the whole body for early signs of skin cancer.

## 

**19 Men’s Perspectives for Future Melanoma Prevention Programs**

## 

Benchimol-ElkaimBrandon^1^MoranChelsea^2^KörnerAnnett^1^GellerAlan^3^CoroiuAdina^3^1McGill University, Montreal, QC, Canada2University of Calgary, Calgary, AB, Canada3Harvard University, Cambridge, MA, USA

 

## 1. Background/Rationale or Objectives/Purpose

Melanoma is the deadliest skin cancer. Middle-age men are often diagnosed with more advanced melanoma, have worse prognosis, and face higher mortality rates compared to women in the same age groups and younger men. Currently, there are no early detection interventions that specifically target men. The present study aims to identify relevant components for future programs and campaigns targeting skin cancer prevention and early detection in men. 

## 2. Methodology or Methods

Semi-structured telephone interviews were conducted with male melanoma patients. Transcripts were coded to develop a comprehensive understanding of recommendations for future prevention interventions. The analysis was conducted across the entire sample, and we looked for similarities and differences in patient perspectives by disease severity (tumor thickness 0–2 mm versus 2–4 mm).

## 3. Impact on Practice or Results

The sample included 38 men (*M_age diagnosis_ =* 63.5 years, SD = 11.8, 21 with 2–4 mm thickness). We identified several themes relevant to future melanoma prevention campaigns, including limited awareness of risk factors for melanoma, limited knowledge about melanoma early detection, concerns about costs of participation in prevention programs (e.g., financial, time, psychological), and barriers to engagement (e.g., feelings of apathy and invincibility, the applicability of delivery method). The themes were similar across the two severity groups.

## 4. Discussion or Conclusions

Our study suggests that future public health campaigns targeting men’s risk for melanoma might benefit from including information about disease prevention and risk factors and address certain concerns and barriers identified herein. Our next step is to develop and test a brief social media campaign targeting men’s awareness about melanoma. 

## 

*Abstract Theme: Innovation in Psychosocial Oncology Interventions*

**12 Spirituality and Cancer Weekend Online Retreat—An Educational and Experiential Workshop**

## 

RutledgeRobDalhousie University, Halifax, NS, Canada

 

## 1. Background/Rationale or Objectives/Purpose

People affected by cancer often struggle with existential and spiritual issues, yet few explore this facet of their humanity with a psychosocial professional. 

The purpose of this workshop is to (a) describe the agenda and evaluation of a weekend retreat specifically addressing spirituality in the context of a cancer diagnosis, (b) provide the workshop attendees with an experience of some of the retreat exercises, and an opportunity to discuss their personal/professional reflections about spirituality in cancer care, and (c) share techniques of maintaining group focus and connection using the Zoom platform for day-long or multi-day programs. 

## 2. Methodology or Methods

Twenty-seven cancer survivors/family members from multiple provinces/states on 24 screens joined a hospital chaplain, psychotherapist, and oncologist for a 2.5 day (16 h) weekend retreat program offered by Zoom. The agenda included didactic non-religious teaching, meditation, visualization, art therapy, and small and large group discussion within a supportive–expressive support group. 

## 3. Impact on Practice or Results

Twenty people completed self-administered questionnaires by email at the end of the weekend. The average score for the entire retreat was 9.5 out of 10. The evaluation comments were overwhelmingly positive. All elements of the program scored relatively high. 

## 4. Discussion or Conclusions

The Spirituality and Cancer weekend online retreat was highly rated by the attendees. The Zoom platform offers a medium in which we can successfully offer programming to distant attendees either as a standalone or as an adjunct to in-person programming. 

## 

**53 Reauthoring Self through Online Digital Storytelling as Art Therapy during COVID**

## 

KlassenBevan^1^DuffMiriam^2^1WHEAT Institute, Winnipeg, MB, Canada2CancerCare Manitoba, Winnipeg, MB, Canada

 

## 1. Background/Rationale or Objectives/Purpose

COVID-19 set conditions for CancerCare Manitoba to pivot delivery of psychosocial services. Therapeutic digital storytelling (TDS) offered a natural online fit. Using film production techniques, TDS empowers participants to create short videos that hold feelings and reframe stories in a clinical context.

## 2. Methodology or Methods

Young adults are in the process of defining self when cancer disrupts their lives. Since technology is a primary way this group communicates, TDS provides relevant intervention for meaning making.

TDS is based on filmmaking psychotherapy methods (Joshua Cohen, Lauren Johnson, Penelope Orr) and narrative therapy (Michael White). Informed by these perspectives, two approaches were developed by an art therapy student with a filmmaking background and a psychosocial oncology clinician trained in expressive arts therapy.

A TDS group pilot was proposed for young adults. Due to time constraints, there was limited uptake; therefore, program development was facilitated with a patient consultant. Subsequent program variations showed increased participation. Individual TDS counselling sessions were offered to a young father with brain cancer who aspired to create a legacy video and reauthor his personal story. Participants reported an increase in hope, confidence, connectedness, and playful curiosity. 

## 3. Impact on Practice or Results

TDS offers an innovative online process to support cancer patients in eliciting emotional content, meaning making, and identity integration. The digital story is an immersive medium to understand patient experience.

## 4. Discussion or Conclusions

TDS requires participants to possess technology literacy and invested curiosity. A knowledgeable therapist can facilitate this process using just-in-time learning to support self-efficacy. TDS is a potent multi-modal process we are only beginning to understand. 

## 

**89 The Sibling Support Project: Supporting Siblings of Children with Brain Tumors through Innovative Educational Tools**

## 

ThuraisinghamNilaniWassermanSydneyTraceyLeahSaikaleyThomasWangChuyuePépinMéganeFilionFrançoiseGrugel-ParkAdrianaMcGill University, Montréal, QC, Canada

 

## 1. Background/Rationale or Objectives/Purpose

An estimated 50,000 Canadians are diagnosed with a brain tumor every year. The Brain Tumour Foundation of Canada (BTFC) has created numerous programs to support this population, although there still lacks sufficient support for the siblings of children with brain tumors. The goal of this project was to fill this gap by creating an accessible and age-appropriate support resource for the siblings and families of children with brain tumors.

## 2. Methodology or Methods

The Population Health Promotion Model and the Dual Coding Theory were the chosen learning frameworks for establishing the two bilingual educational resources: (1) the interactive storybook that allows for siblings to explore and gather information independently, and (2) the e-booklet, which is designed for older siblings or for parents. Evidence-based information was derived from research studies and interviews with a social worker and affected families.

## 3. Impact on Practice or Results

A survey among staff from the BTFC and professors indicated that 100% of participants (a) found that the interactive storybook and e-booklet will support siblings and parents of children with brain tumors, (b) thought the storybook’s language was child friendly, (c) thought the e-booklet content was clear, concise, and pertinent, and (d) claimed that they will recommend these resources to BTFC families.

## 4. Discussion or Conclusions

The BTFC believed that this project will have a lasting impact by permitting children to voice their concerns and develop coping skills after their sibling’s diagnosis. The storybook accommodated the health literacy of the younger target audience. Collaboration with the BTFC allowed for dissemination of resources, rendering them openly accessible.

## 

**107 Access to Speech Language Pathology Services for Head and Neck Cancer Patients in Ontario**

## 

WangJenneyZwickerVictoriaNazeri-RadNargesQuilliamElizabethFoxColleenWrightMeaghanPeizaPatrice deOntario Health, Toronto, ON, Canada

 

## 1. Background/Rationale or Objectives/Purpose

Ontario Health–Cancer Care Ontario (OH-CCO) recommends that all head and neck cancer patients should receive care from a Speech Language Pathologist (SLP) during their cancer treatment. Furthermore, SLP and other specialized Psychosocial Oncology (PSO) providers should be part of the core care team for this patient population. Currently, nine of the 14 regional cancer centers (RCCs) in Ontario treat head and neck cancer patients, but only four RCCs have SLPs. The purpose of this analysis was to understand current levels of access to and gaps in SLP services for head and neck cancer patients in hospital-based outpatient settings.

## 2. Methodology or Methods

SLP utilization data from RCCs was analyzed to understand the percentage of head and neck patients currently accessing outpatient SLP services, to identify variations in care and gaps in SLP services, and to inform planning to increase access to SLP services. 

## 3. Impact on Practice or Results

The results of the analysis suggest that many head and neck cancer patients are unable to get timely access, or do not have any access, to outpatient SLP services. This represents a significant gap in head and neck cancer patients’ standard of care. This is also an opportunity to improve organizational capacity and increase SLP staffing to meet patient needs.

## 4. Discussion or Conclusions

OH-CCO is exploring opportunities to introduce new SLP quality indicators to track performance and will work with partners in the RCCs to ensure accurate reporting of SLP data. Performance will be socialized with regional stakeholders to promote increased access to SLP services for head and neck patients.

## 

*Abstract Theme: Oncology Clinicians: Workplace Issues, Multidisciplinary Collaboration, and Resilience*

**28 Toward a Shared Conception of Resilience at Work among Cancer Care Professionals: Mobilizing the EnRICH Framework**

## 

GiordanoEmilie^1^TremblayDominique^1^,^2^TurcotteAnnie^1^1University of Sherbrooke, Longueuil, QC, Canada2Centre de recherche Charles-Le Moyne—Saguenay–Lac-Saint-Jean sur les innovations en santé (CR-CSIS), Longueuil, QC, Canada

 

## 1. Background/Rationale or Objectives/Purpose

Cancer care professionals experience difficult situations on a daily basis, which have been recently compounded by adversity related to the COVID-19 pandemic. Researchers, managers, and professionals are seeking to achieve a shared conception of resilience at work in order to support its development. This presentation aims to address this challenge by presenting the explanatory potential of the EnRICH framework applied to the cancer care context.

## 2. Methodology or Methods

The central concept in the EnRICH framework is the adaptive capacity at the heart of a resilient community. This model is well suited to the cancer care context, where professionals work in interdisciplinary teams. Based on a salutogenic approach, the framework focuses on health promotion rather than disease management. Leadership, resource management, communication, and engagement encourage the identification of strategic interventions. Consideration of culture and of the complexity of the process of developing resilience allow for interventions adapted to this context.

## 3. Impact on Practice or Results

The EnRICH framework provides a representation of the factors and processes that help target upstream and downstream interventions to promote resilience. The framework also enables the identification of adaptive capacities that can be used to operationalize resilience.

## 4. Discussion or Conclusions

Originally applied in situations of disaster, this framework also holds promise for day-to-day adversity, as it emphasizes continual development of resilience. Its use on the cancer care context can guide future research as well as front-line interventions.

## 

**59 Using Video Vignette Methodology to Support Cancer Team Resilience at Work: A Qualitative Study**

## 

TurcotteAnnieTremblayDominiqueGiordanoÉmilieLessardSylvieUniversity of Sherbrooke, Longueuil, QC, Canada

 

## 1. Background/Rationale or Objectives/Purpose

The importance of provider resilience to face day-to-day challenges has come to the fore in the pandemic context. Interventions to support resilience at work among cancer teams remain understudied. This presentation reports on the utilization of video vignettes to identify context elements and mechanisms likely to enhance team resilience at work. 

## 2. Methodology or Methods

Three short videos depicting professionals as they deliver news of a cancer diagnosis or disease progression were used to explore how teams face and bounce back from stressful situations. Qualitative data were collected in three focus groups where interdisciplinary team members viewed and discussed each video segment, which was followed by debriefing sessions with managers and professional representatives. Analysis used a descriptive–interpretive approach and coding strategy built on an adapted version of Gucciardi’s multilevel conceptual model of facilitating factors. 

## 3. Impact on Practice or Results

Video vignettes are relevant in exploring clinical situations and context issues experienced by cancer teams. They elucidate teaming and reflexive mechanisms activated to create healthy work environments and team resilience. They highlight that alongside informal support, it is important to set aside formal and regular moments to identify and access resources, support one another, handle difficult clinical situations, consolidate team cohesion, and understand team leader decisions. 

## 4. Discussion or Conclusions

Our results suggest that the vignette method helps cancer teams reflect on their resilience and recognize their strengths, challenges, and opportunities to build on these strengths. Results support the integration of video vignettes into interventions aimed at supporting team resilience in the Quebec Cancer Network.

## 

*Abstract Theme: Cancer Treatment-Related Symptom and Toxicity Management*

**79 Prevalence and Predictors of Longitudinal Trajectories of Distress in Cancer Populations: A Latent Class Growth Analysis Approach**

## 

PanjwaniAliza^1^SadeCristina^2^GaoJianhui^1^Espin-GarciaOsvaldo^1^LiMadeline^1^1Princess Margaret Cancer Centre, Toronto, ON, Canada2Instituto Nacional del Cancer, Santiago, RM, Chile

 

## 1. Background/Rationale or Objectives/Purpose

This study uses latent class growth analysis (LCGA) in one of the largest cohorts of individuals diagnosed with cancer to identify the following: (1) prevalence of distress across cancer types; (2) longitudinal distress trajectory classes; and (3) baseline covariates that predict trajectory class membership. 

## 2. Methodology or Methods

*Sample and setting:* Adult ambulatory oncology patients across all 12 tumor sites at the Princess Margaret Cancer Centre who completed the Distress Assessment and Response Tool (DART) as part of routine care between March 2013 and March 2018 and had at least one follow-up screening (n = 25,534) were included. 

*Procedure*: Distress analysis was based on DART data, including ESAS-r anxiety and depression, PHQ-9, and GAD-7. Other DART elements (i.e., psychiatric history, ESAS-r physical symptom burden, ECOG functional status, and SDI-21/CPC social difficulties) were included as predictors. Demographic, medical, and psychosocial service use data were also extracted.

## 3. Impact on Practice or Results

Five distinct latent classes of distress were identified using ESAS-r anxiety and depression, respectively: high chronic (4.4%, 2.9%), delayed (15%, 12.9%), subsyndromal (12%, 9.5%), recovered (13.9%, 13.6%), and resilient (54.8%, 61.1%). PHQ-9 and GAD-7 identified fewer, more longitudinally stable latent classes. Group membership in the delayed category included physical symptom burden for ESAS-r anxiety and depression; advanced cancer stage was only significant for depression. High-chronic membership for depression was predicted by physical symptom burden (OR: 1.3) and greater cognitive impairment (OR: 2.6) but not advanced cancer stage. Analyses of psychosocial and cancer-site specific predictors are being finalized.

## 4. Discussion or Conclusions

Most cancer patients show a resilient distress trajectory, while 20% remain high in their distress over time or show delayed symptoms. Identifying predictors of distress trajectories will inform prophylactic and tailored psychological interventions for these vulnerable populations.

## 

*Abstract Theme: Pandemics and Cancer Care Issues*

**31 Delivering Tele-Health Psychosocial Care in Times of COVID-19: Lessons Learned in Supporting Oncology Patients during a Worldwide Pandemic**

## 

ThibodeauKimberleyBernatchezMarie SolangeHamelMarcMcGill University Health Centre, Montreal, QC, Canada

 

## 1. Background/Rationale or Objectives/Purpose

With the surge of the COVID-19 pandemic, the psychosocial needs of patients with cancer became even more pressing. The patient’s emotional distress rose as institutions were forced to delay oncology treatments and refuse to allow patients to be accompanied to their appointments or be with a loved one when admitted to the hospital. Patients’ emotional distress also increased with the fear of being exposed to the virus. Social isolation and uncertainty became common stressors. For mental health professionals, the challenge became how to continue providing quality psychosocial care. Tele-health interventions became the new model of care delivery. For many therapists, this was unchartered territory. The following workshop will highlight lessons learned in the delivery of psychosocial care to oncology patients since COVID-19. 

## 2. Methodology or Methods

1. Present common stressors identified by patients in times of COVID-19 (many that were not present prior to the pandemic);
2. Highlight unique challenges for psychosocial therapists in delivering interventions (adjusting to tele-health practice, building a therapeutic alliance at a distance);
3. From clinical vignettes, identify lessons learned from remote psychosocial practice;
4. Invite participants to exchange on how the pandemic affected their work.

## 3. Impact on Practice or Results

Our short experience delivering psychosocial interventions remotely indicate that it is feasible and well accepted by patients.

## 4. Discussion or Conclusions

While the pandemic will someday be a thing of the past, tele-health psychosocial practice is here to stay. Many healthcare professionals have had to adapt without much experience. Many lessons have been learned and others remain. This workshop will be an opportunity to begin this exchange.

## 

**43 Medical Assistance in Dying (MAiD) and COVID-19: The Impact of the Pandemic on the Experience of Patients and Their Family Caregivers**

## 

TongEryn^1^NissimRinat^1^,^2^ThangarasaTharshika^1^,^2^SelbyDebbie^3^BeanSally^3^Isenberg-GrzedaElie^2^,^3^RodinGary^1^,^2^LiMadeline^1^,^2^HalesSarah^1^,^2^1Department of Supportive Care, Princess Margaret Cancer Centre, University Health Network, Toronto, ON, Canada2Department of Psychiatry, University of Toronto, Toronto, ON, Canada3Sunnybrook Health Sciences Centre, Toronto, ON, Canada

 

## 1. Background/Rationale or Objectives/Purpose

The COVID-19 pandemic and its containment measures have drastically altered the end-of-life and grief experiences, including medical assistance in dying (MAiD). The purpose of this study is to understand how the pandemic impacted the MAiD experience of patients and caregivers.

## 2. Methodology or Methods

Patients requesting MAiD during COVID-19 and their caregivers were recruited from the University Health Network, Sunnybrook Health Sciences Centre, and GTA MAiD Community group between March 2020 and March 2021. Semi-structured interviews were conducted with seven patients (86% cancer diagnosis) and nine caregivers about their experience with MAiD after the request. Six months following patient death, eight bereaved caregivers were re-interviewed or recruited to participate in an interview to explore their bereavement experience. Transcripts were analyzed using thematic analysis.

## 3. Impact on Practice or Results

Qualitative analysis revealed several themes to understand the impact of COVID-19 on the MAiD experience. Both patients and caregivers felt an increasing sense of isolation and disconnection, which impacted all phases of the MAiD process, from assessments to intervention day, and extended into bereavement for caregivers. The circumstances of COVID-19 also accelerated the decision-making process around MAiD. 

## 4. Discussion or Conclusions

Participants provided important insights into the challenging impact of the pandemic on the MAiD, quality of dying, and grief experiences. The importance of recognizing the social and relational dimensions of the MAiD experience is highlighted, particularly in the context of COVID-19 and its containment measures. These findings may help inform clinical practice to better support the MAiD and bereavement processes during the pandemic and beyond.

## 

**80 Coping with the COVID-19 Pandemic While Living with Cancer: An Exploratory Cross-Sectional Study Examining Psychological Distress and Post-Traumatic Growth**

## 

ZhangKaren^1^,^2^LukosiusDenise Bryant^3^,^4^PondGreg^4^EllisPeter^1^,^4^SussmanJonathan^1^,^4^TozerRichard^1^,^4^BrownMichael^1^,^2^WasermanJessica^1^,^2^LocisRuth^1^MeyersRalph^1^,^4^1Juravinski Cancer Centre, Hamilton, ON, Canada2Department of Psychiatry and Behavioural Neurosciences, McMaster University, Hamilton, ON, Canada3School of Nursing, McMaster University, Hamilton, ON, Canada4Department of Oncology, McMaster University, Hamilton, ON, Canada

 

## 1. Background/Rationale or Objectives/Purpose

While preliminary findings show that patients with cancer report high levels of depression during the COVID-19 pandemic, the clinical picture is convoluted by data indicating that they also report lower levels of anxiety than the general population. To date, no study has examined cancer patients’ adjustment to the pandemic in terms of both psychological distress and resilience (post-traumatic growth). There is also little known about whether health beliefs and behaviors in response to COVID may be associated with a patient’s negative and/or positive adjustment to the pandemic. This study aims to (1) identify levels of psychological distress and post-traumatic growth among patients diagnosed with cancer during pandemic; and (2) assess whether pandemic-related behavioral responses and illness perceptions predict psychological distress and post-traumatic growth. 

## 2. Methodology or Methods

A target sample size of 104 patients attending appointments at the Juravinski Cancer Centre were recruited between February and May 2021. Inclusion criteria included patients who are newly diagnosed, on active treatment, and/or within 5 years post active treatment of cancer.

Standardized questionnaires assessing demographic information, post-traumatic growth, psychological distress, illness perceptions, and behavioral responses to the COVID-19 pandemic were administered as electronic or paper copy surveys. Disease-related information were extracted from patient health records. Descriptive, univariate, and multivariable logistics and linear regression analyses will be conducted. 

## 3. Impact on Practice or Results

Data collection is currently in progress. 

## 4. Discussion or Conclusions

Study results will inform the development of evidence-based self-management interventions aimed at reducing psychological distress and leveraging resilience among patients diagnosed with cancer. 

## 

**104 Canadian Cancer Society** **’s Cancer Information Helpline: An Effective Adjunct Model for Meeting the Informational, Emotional, and Practical Needs of Canadians Affected by Cancer Prior to and during the COVID-19 Pandemic**

## 

LeeSharon^1^HollyBradley^2^FitchMargaret I.^3^BurnettLaura^1^CoyTiffany^1^1Canadian Cancer Society, Hamilton, ON, Canada2Canadian Cancer Society, Toronto, ON, Canada3University of Toronto, Toronto, ON, Canada

 

## 1. Background/Rationale or Objectives/Purpose

Cancer creates a wide range of psychosocial needs throughout the cancer continuum. In 2020, the pandemic exacerbated the experience of Canadians given its disruption to the cancer care system, isolation, and interruption of practical programs.

The Cancer Information Helpline (CIH) is a model of care that provides compassionate emotional support and information on any aspect of cancer and assistance in finding cancer-related services.

## 2. Methodology or Methods

In 2020, the CIH implemented a post-inquiry evaluation of a select subset of clients to assess the immediate impact of the service. A thematic analysis of the brief survey responses was undertaken and revealed the most frequently identified reasons for calling, satisfaction indices, and psychosocial outcomes. 

Overlapping the timeframe of the CIH survey content related to COVID-19 addressed in CIH inquiries, such as access to practical supports and magnified isolation impacting clients’ psychosocial needs, was also analyzed. 

Findings from both analyses will be presented. 

## 3. Impact on Practice or Results

Results affirm the wide range of psychosocial needs for which Canadians seek help and the additional burden of the pandemic. These results reinforce the value of a high-quality resource, such as the CIH, to serve as an adjunct to the cancer care system.

## 4. Discussion or Conclusions

Several topics will be discussed including person-centered care for Canadians affected by cancer and the complications from COVID-19, the CIH as a proven model of care, healthcare provider referral to CIH, and the Community Services Locator, which is a national tool to locate cancer-related services. 

## 

**110 Returning to Work after Breast Cancer: Careers Impacted by COVID-19**

## 

WouterlootEliseUniversity of Toronto, Toronto, ON, Canada

 

## 1. Background/Rationale or Objectives/Purpose

**Objective.** For cancer survivors, returning to work means returning to normalcy and a sense of well-being. Yet many face challenges that prevent them from working in a full and fulfilling way after cancer. This poster is part of a qualitative dissertation on how breast cancer survivors overcome career obstacles and find new meaning in their working life. The aim is to understand their return-to-work experiences, including how they adapt, and whether their personal purpose and goals for work have changed since having cancer.

**Framework.** Their career planning, goal setting, and ability to overcome obstacles are viewed through the lens of the Career Human Agency Theory (Chen, 2015). 

## 2. Methodology or Methods

**Sample.** During the summer of 2020, semi-structured interviews were conducted via phone or video calls with 12 Canadian breast cancer survivors. During these interviews, all participants spontaneously raised the variable impact of the COVID-19 pandemic on their post-cancer return to work. 

## 3. Impact on Practice or Results

**Results.** Thematic analysis revealed how they experience job loss, unplanned early return to work, concerns about working when immunocompromised, social isolation as a reminder of cancer treatment, and in some cases, gratitude, or relief for the extended working from home arrangements. 

## 4. Discussion or Conclusions

**Implications.** These findings will shed light on the impact of the COVID-19 pandemic on lives already touched by a critical illness and help to inform the support available for cancer survivors returning to work in the coming year.

## 

*Abstract Theme: Other Value-Based and Person-Centered Cancer Care*

**6 Physiological Synchrony in Cancer Consultations**

## 

VigierMarta^1^,^2^ThorsonKatherine^3^AndritschElisabeth^1^FarkasClemens^1^SchwerdtfegerAndreas^2^1Medical University Graz, Graz, STM, Austria2University of Graz, Graz, STM, Austria3Barnard College Columbia University, New York, NY, USA

 

## 1. Background/Rationale or Objectives/Purpose

Several studies reported different types of influence (i.e., behaviors, subjective experiences) to occur in doctor–patient interactions. The current research examined whether doctors and patients might influence each other’s physiological responses.

## 2. Methodology or Methods

We collected interbeat interval responses (IBI) during oncological consultations (N = 102 unique doctor–patient interactions) to examine the physiological linkage between doctors and patients. Precisely, we estimated whether the physiological response of one person—the “sender”—at one time interval predicts the physiological response of the other person—the “receiver”—at a subsequent time interval and whether this changes as a function of role (doctor vs. patient) and length of a doctor–patient relationship.

## 3. Impact on Practice or Results

We found that physiological linkage between doctors and patients varied by an interaction between role and relationship length (in a non-linear fashion): b = −0.01, *p* = 0.005, R2 = 0.07. Doctors’ physiological responses predicted patients’ physiological responses in medium-length relationships: *ps* < 0.05. However, patients were not linked to their doctors in shorter or longer relationships. Doctors were never significantly linked to their patients: *ps* > 0.13.

## 4. Discussion or Conclusions

These findings suggest that physiological influence from doctors to patients can occur over time and might potentially impact patients’ health.

## 

**17 Relationship Quality and Hopelessness in Ovarian Cancer Patients: The Role of Emotion Dysregulation**

## 

AtlasMaya^1^FergusonSarah^2^HartTae^1^1Ryerson University, Toronto, ON, Canada2University Health Network, Toronto, ON, Canada

 

## 1. Background/Rationale or Objectives/Purpose

Hopelessness is a common response to illness and is associated with suicidal ideation and lower quality of life. Poor relationship quality contributes to greater hopelessness in gynecological cancer patients, yet the mechanism underlying this association is unclear. The *social influence hypothesis* suggests that emotion dysregulation may be a mediator. The current study aimed to uncover if (1) emotion dysregulation mediated the association between relationship quality and hopelessness and (2) if certain emotion dysregulation facets were unique in explaining this association.

## 2. Methodology or Methods

**Sample and setting:** Women with a diagnosis of ovarian cancer in an intimate partnership (N = 197) were recruited from the Princess Margaret Hospital in Toronto, Ontario as part of a larger study. 

**Procedures:** Patients completed validated self-report questionnaires. Using structural equation modeling, mediational models were tested with emotion dysregulation as a latent construct with six subscales (Model 1) and six emotion dysregulation subscales as mediators (Model 2). 

## 3. Impact on Practice or Results

Model 1 exhibited good fit, χ^2^ (15) = 23.13, p = 0.08, CFI = 0.98, TLI = 0.96, SRMR = 0.04, RMSEA = 0.06. The total model accounted for 6.2% of the variance in hopelessness. Model 2 was not statistically supported. 

## 4. Discussion or Conclusions

The current study provides preliminary support for the *social influence hypothesis* in cancer populations. While statistically significant, the small variance accounted for by this model suggests that among ovarian cancer patients, emotion dysregulation may not be a fruitful target to reduce hopelessness. 

## 

**32 Development of an Electronic Caregiver-Reported Outcomes (CROs) Program: A Qualitative Study on Caregivers’ Preferences**

## 

LambertSylvie^1^,^2^LobanKatya^1^,^2^FariaRosana^3^MagalhaesMona^2^IbbersonCindy^2^ProjeanKarine^2^ClaveriaAlexandra^2^LazarisAriana^2^1McGill University, Montreal, QC, Canada2St. Mary’s Research Centre, Montreal, QC, Canada3St. Mary’s Hospital Centre, Montreal, QC, Canada

 

## 1. Background/Rationale or Objectives/Purpose

The COVID-19 pandemic has uncovered the significant roles and responsibilities that caregivers take on and the ensuing distress they experience. The purpose of this study was to explore the acceptability of screening for caregiver-reported outcomes (CROs) among cancer caregivers and identify key components for a CRO screening program.

## 2. Methodology or Methods

Phone/online semi-structured interviews were conducted with 21 cancer caregivers recruited from cancer centers, online, and community organizations. Interview questions focused on the following: acceptability of CRO screening, types of CROs and timing/frequency of screening, preferred resources following screening, and communication of CROs to patients and clinicians. Interviews were audio-recorded, verbatim transcribed, and coded.

## 3. Impact on Practice or Results

All caregivers welcomed the possibility of completing CRO questionnaires and saw it as an opportunity for reflection. Caregivers prioritized screening for emotional symptoms followed by physical symptoms and practical issues. Most caregivers wanted results shared with the patient’s treating team. Conversely, the majority did not want results to be shared with the patient nor their primary care physician. Most caregivers viewed CRO screening as a means to an end that would lead to the offer of resources; it is potentially a one-stop-shop program where they could find self-management information and guidance on caring for the patient. Opinions regarding the timing and frequency of CRO screening differed; many felt it was not appropriate in the initial diagnosis phase as caregivers are settling into their role. Periodic administration (15–20 min maximum) was deemed appropriate.

## 4. Discussion or Conclusions

This is one of the first studies to examine how screening for caregivers’ distress can be integrated in cancer care. 

## 

**60 Caregiver Re-Negotiation of Sense-Of-Self: Considerations for Caregiver-Reported Outcomes in the Context of Colorectal Cancer**

## 

AveryJonathan^1^,^2^HowardFuchsia^1^TorrejonMaria^3^BeckScott^1^,^3^LynchKelsey^1^PorcinoAntony^3^ThorneSally^1^WolffAngela^4^BoardPatient Partner Advisory^1^McKenzieMichael^3^1University of British Columbia, Vancouver, BC, Canada2Princess Margaret Cancer Centre, Toronto, ON, Canada3BC Cancer, Vancouver, BC, Canada4Trinity Western University, Langley, BC, Canada

 

## 1. Background/Rationale or Objectives/Purpose

Objective/Purpose: The colorectal cancer (CRC) journey can be complex and demanding on caregivers, contributing to caregiver burden. Our purpose was to explore if/how caregiving for a person with CRC affects the caregiver’s sense of self, that is the way an individual defines, locates, and differentiates self from others. Findings will be used to develop and integrate caregiver reported outcomes (CROs) to better support CRC caregivers.

## 2. Methodology or Methods

Methods: In this patient-oriented, qualitative interpretive description research, our interdisciplinary team recruited participants primarily from British Columbia with a subgroup from other provinces. We conducted and analyzed semi-structured interviews with 25 caregivers and 37 patients with CRC using inductive coding and constant comparative methods. 

## 3. Impact on Practice or Results

Results: As caregivers assumed new roles and responsibilities to ensure the patient had the care required, the caregivers constantly re-negotiated their sense of self. This re-negotiation included shifts in how they relate to others and their ability/inability to retain some semblance of an eroding sense of individuality as more time was spent caring and advocating for the patient and less time carrying for themselves. Re-negotiation contributed to (1) varying degrees of distress, (2) a degrading of sense of self, and (3) a loss of connection with others. Caregivers commonly attempted to find a silver lining as a coping strategy throughout the CRC trajectory. 

## 4. Discussion or Conclusions

Discussion/Conclusions: Recognition of caregiver re-negotiation of sense of self, the negative impact on some, and the strategies used to cope provide direction for the development and/or adaptation of relevant CROs. 

## 

**97 “Should We Do This Together?” A Scale to Assess the Dyadic Expectations (DE) of Couples Coping with Cancer**

## 

BovenschenKendra^1^KörnerAnnett^2^BrosseauDanielle C.^1^1The King’s University, Edmonton, AB, Canada2McGill University, Montreal, QC, Canada

 

## 1. Background/Rationale or Objectives/Purpose

To maximize available psychosocial resources, personalized support for couples coping with cancer is proving necessary. The present study focused on developing a scale to assess patients’ and/or partners’ dyadic expectations (DE). DE represent the extent to which couples believe the multidimensional impacts of cancer should be managed together or individually.

## 2. Methodology or Methods

Participants were recruited from two urban cancer centers. Patients (N = 252) were diagnosed with cancer within the last two years, completing active treatment (or within 6 months of completion), and in a committed relationship for at least one year. Partners (N = 209) were in a committed relationship with an eligible patient. Participants completed a cross-sectional survey that included the seven-item DE scale and measures used to investigate validity hypotheses. Exploratory factor analyses using principal axis factoring with direct oblimin rotation was employed to investigate the factor structure of the scale among patients and partners respectively. 

## 3. Impact on Practice or Results

A unidimensional scale explaining 59% of the variance in patients’ DE and 58% of the variance in partners’ DE was identified. Item loadings ranged from 0.66 to 0.79 for patients and 0.55 to 0.78 among partners. The singular dimension of the scale suggests that patients and partners consistently reported DE across domains of cancer-related coping. Levels of DE and evidence of the scale’s reliability and validity will also be reported. 

## 4. Discussion or Conclusions

The DE scale is quickly administered and appropriately reflected by a total score. This tool can optimize psychosocial resource use by helping clinicians discern which patients would benefit the most from couple-focused psychosocial support.

## 

**108 How Patients** **’ Personal Interests, Culture, and Life Experiences Can Lead to New Pathways in Goal Attainment**

## 

LadouceurMelinaBarrett-RobillardPatriciaWoodardStephanieOttawa Regional Cancer Foundation, Ottawa, ON, Canada

 

## 1. Background/Rationale or Objectives/Purpose

Over the last decade, there has been a growing recognition of the importance of understanding a patient’s culture, interests, and values in clinical practice and offering more person-centered care. Approaches such as Health Coaching, Strength-based Social Work Practice, and Acceptance and Commitment Therapy help to uncover a person’s strengths, values, and motivations and utilize powerful questioning to help patients discover how they might make health behavior changes. These approaches, by their very nature, attempt to be sensitive to the importance of culture in people’s lives. This symposium aims to provide healthcare professionals with powerful questions they can integrate in their practice to help patients in making changes toward their health. We will be sharing stories from our clients, questions we asked them, and the unique strategies they came up with to attain their goals, which were strategies that fit into their lives and resonate with who they are.

## 2. Methodology or Methods

Powerful questioning is a key component of our health coaching intervention. We have helped more than 3000 families through our Cancer Coaching program.

## 3. Impact on Practice or Results

Nearly all (97%) of our clients say they are better able to cope with life, 93% feel more confident that they can do something about their health, and 90% say that their quality of life has improved. According to the Harvard Institute of Coaching, coaching outperforms advice-giving based approaches in 80% of clinical trials.

## 4. Discussion or Conclusions

Whether it’s Aboriginal bead making as a way to cope with stress or the sense of purpose gained by a cancer patient who pushes himself to be active by tending to the garden, our clients have unique ways of coping and creating behavior changes, by connecting with what is meaningful to them.

## 

**116 The Unique Psychosocial Needs of Brain Tumor Patients: Revamping Support Needs Surveys and Practices of the 21st Century**

## 

MashmoushiYasmina^1^FongGeoffrey^1^,^2^1University of Waterloo, Waterloo, ON, Canada2Ontario Institute for Cancer Research, Toronto, ON, Canada

 

## 1. Background/Rationale or Objectives/Purpose

Although cancer patient support needs surveys and support care practices routinely employ a “one-size-fits-all” approach, distinct cancer populations identify unique challenges/needs, resilience factors, and coping methods. Brain tumor patients are one example. Since the brain is such a fundamental part of who we are as individuals (i.e., encompasses our essential, everyday cognitive toolkit, dictates our very personalities and moods, and stores our most precious of memories), brain tumor patients endure psychosocial challenges not shared by other cancer patient populations. Moreover, their cancer diagnosis poses unique challenges that render their experiences divergent from those of neurological patient populations.

## 2. Methodology or Methods

Methodology consists of an online, anonymous survey sent out through the BTFC. A mixed-methods approach, whereby both qualitative and quantitative data are collected and analyzed, was used.

## 3. Impact on Practice or Results

Results are tentative and expected to be finalized by 25 June 2021. 

## 4. Discussion or Conclusions

The goal of this research was to pinpoint emergent challenges particular to the neuro-oncological diagnosis, which render the dual nature of the diagnosis (oncological and neurological) greater than the sum of its parts. These emergent properties have been neglected by needs questionnaires administered to neuro-oncological patients (e.g., the SCNS-LF59 given to cancer patients and brain tumor patients) and by psychosocial care provision services (counseling/psychological services) catered to this patient population. Implications also exist for neuro-oncologists: Increased awareness of these challenges/needs can increase sensitive administration of care to patients and greater understanding of patients’ wishes concerning treatment and end-of-life care. These unique psychosocial challenges/needs of brain tumor patients also coincide with unique resilience factors for this population. Awareness and dissemination of these factors to patients, supportive care providers, and medical care providers can help better foster resilience in this patient population.

## Author Name and Program Codes

Ommos, Cara8Abaraogu, Ukachukwu O.44Abolhosseini, Shokouh
94
Adams-Beavereye, Dakota114Al Awar, Zeina33Alanazi, Jamilah
10
Alkhenaini, Hamad76Allapitan, Elleine26Alston, Nicole
22
An, Ekaterina58Andritsch, Elisabeth6Anyachukwu, Canice C.44Atlas, Maya
17
Attieh, Samar
39
Audrey, Plante96Avery, Jonathan37, 58, 60, 70, 72Babiker, Dina
57
Babinski, Stephanie
69
Balneaves, Lynda G.
49
Bansal, Mannat61, 63Barbeau, Anne73, 78Barr, Ronald109Barrett-Robillard, Patricia106, 108Baruch, Melanie70Bastas, Denise112Baydoun, Mohamad82, 91Baziliansky, Svetlana
45
Bean, Sally43Beattie, Sara23Beck, Scott60Bedrossian, Nathalie14Belzile, Eric81Benchimol-Elkaim, Brandon
19
Bender, Jacqueline58, 69Beny, Alexander45Bergeron, Catherine2Bernatchez, Marie Solange31Big Smoke, Arrow50Bilash, Tristan18Bining, Mira81Birnie, Katie35, 82Black, Kathleen
55
Bomfim, Emiliana
114
Boss, Harrison8Boulanger, Josée29Bovenschen, Kendra
97
Bridel, William15, 20, 63Brosseau, Danielle C.97Brown, Michael80Brown, Mitchell71Bryant Lukosius, Denise80Bubber, Vikram
27
Bultz, Barry93Burnett, Laura104, 71Burr, MISt, Sabrina51Capozzi, Lauren C.90Carlson, Linda35, 82, 85, 91, 92, 94Cartwright, Stephen82Caru, Maxime3Castelli, Samantha
2
Chalifour, Karine48, 69Chambers, Christine47Chapman, Stacy72Chary, Srini4Chen, Amy61Cherak, Stephana
109
Cho, Sara
26
Chomienne, Marie-Hélène54, 56, 57Chu, Alanna58, 68Chung, Joanna7Claveria, Alexandra32Cohen, Miri45Cornelissen, Laura47Coroiu, Adina19Coy, Tiffany104Craig, Bobbie-Ann
20
Crowell, Philip39Culos-Reed, Nicole11, 15, 20, 38, 61, 63, 90D’Addario, Joseph51D’Agostino, Norma69Darmonkow, Georgia5Daun, Julia T.61, 90De Groot, Janet4de Guzman Wilding, Marie90de Oliveira, Claire40de Peiza, Patrice107DeCaria, Kristen
40
Deleemans, Julie109, 91, 92Diaz, Ruth109Dick, Peggy33Doré, Isabelle14, 66, 96Doumanian, Rita51Drake, Emily69Drziak, Rosemary70Duchek, Delaney15, 61Duff, Miriam53, 55Duval, Michel3Easaw, Jay90Eaton, Geoff48Edwards, Annemarie37Egan, Mary29Ellis, Kelsey11, 38Ellis, Peter80Emanuele, Cristina72Espin-Garcia, Osvaldo79Esplen, Mary Jane70Ester, Manuel61Etchegary, Holly
5
Faria, Rosana32Farkas, Clemens6Feldstain, Andrea4Fergus, Karen46, 62Ferguson, Sarah17Fernandez, Conrad47, 7Fidler-Benaoudia, Miranda109Fiest, Kirsten109Filion, Françoise89Fisher, Sara38Fitch, Margaret I.104Fitzgibbon, Kyle58, 73, 78Flanagan, Tanya87Flanders, Annette47Flynn, Michelle94Fong, Geoffrey116Forbes, Caitlin26, 35Forster, Victoria35Fox, Colleen107Francis, George J.90Frattaroli, Samantha
71
Gajtani, Zenel91Gandhi, Manisha62Gao, Jianhui79Garland, Sheila111, 18, 48, 69Gauvin, Lise66, 96Geller, Alan19Gifford, Wendy29, 33Giguère, Lauriane
57
Gillis, Hope39Giordano, Émilie28, 59Goulbourne, Elaine71Graham, Ian33Grassau, Pamela29Green, Craig82Grugel-Park, Adriana89Guilcher, Gregory 7, 38Gupta, Abha69, 72Hack, Thomas8Hales, Sarah43, 58Hamel, Marc31Harris, Cheryl54, 56, 57Harris, Christine37Hart, Tae17Hayward, Emilie N.49Heathcote, Lauren47Heisey, Ruth71Herman, David
62
Holly, Bradley
104
Horst, Teddi65Howard, Fuchsia60, 69Howlett, Melissa23Hunt, Nigel10Ianakieva, Iana62Ibberson, Cindy32Igwe, Sylvester E.44Ilie, Gabriela75, 77Isenberg-Grzeda, Elie43Jaggi, Priya8Jivani, Khairun37Jones, Georden88Jones, Jennifer70Kamen, Charles18Kelly, Brian93Kennedy, Margo76Khan, Amina69Khan, Rumaisa
71
Khlifi, Rabeb100, 41, 42Khu, Melanie23Kiflen, Ruhi
37
King, Judy29, 9King-Shier, Kathryn63Kitchen, Jessica
40
Klassen, Bevan
53
Klein, Roberta Y73, 78Kubant, Alla58Kuhn, Susan7Körner, Annett2, 19, 97Ladhani, Noor Niyar N.46Ladouceur, Melina106, 108Laizner, Andréa Maria39, 70Lamarche, Jani
70
Lambert, Sylvie32, 70, 81Langmuir, Tori54, 56Larocque, Gail54, 56, 57Laverdière, Caroline3Lazaris, Ariana32Leahey, Angela62Lebel, Sophie39, 54, 56, 57, 68, 70, 88Lee, N., Virginia
51
Lee, Sharon
104
Lesiuk, Christine90Lessard, Sylvie59Letendre, Angeline
50
Letoruneau, Nicole7Levesque, Ariane
3
Li, Madeline43, 73, 78, 79Loban, Katya32Locis, Ruth80Loiselle, Carmen G.39Lupichuk, Sasha94Lynch, Kelsey60MacDonald-Liska, Carrie54, 56, 57Mackay, Sarah2MacLeod, Julia47Magalhaes, Mona32, 81Maheu, Christine70, 87Mairena, Paola Michelle Garcia
56
Male, Dana
74
Mashmoushi, Yasmina
116
Mathew, Andrew76McClement, Susan8McDonough, Meghan15, 20, 90McIntyre, Krista38McKenzie, Michael60McKillop, Sarah109Mcknight, Erin65McLaughlin, Emma11, 38McLeod, Albert
95
Mclver, Christine39McNeely, Margaret90Meyers, Ralph80Miljanovski, Melissa58Mlodzik, Kate71Montiel, Corentin
14
Montminy, Sarah66, 96Moran, Chelsea19, 23Morita, Jody76Mouchantaf, B.A., Rola51Mutsaers, Brittany54, 56, 57, 68Nazeri-Rad, Narges107Neville, Alyssa
112
Nguyen, Tina
85
Nissim, Rinat43, 70Nnate, Daniel A.
44
Noel, Melanie35Novy, Christine
36
Oberoi, Devesh85Ould Brahim, Lydia81Pagnutti, Teresa39Pain in Childhood Cancer Survivors Team, the47Panjwani, Aliza73, 78, 79Parkinson, Maureen87Patten, Scott109Patton, Michaela35, 82Petricone-Westwood, Danielle50Piché, Alexia14, 66, 96Piedalue, Katherine-Anne91, 94Pincivero, Natalie70Pink, Jennifer74Plante, Audrey66Poitras, Stéphane9Pond, Greg80Porcino, Antony49, 60Presseau, Justin54, 56, 57Projean, Karine32Punnett, Angela7Pépin, Mégane89Quilliam, Elizabeth107Qureshi, Maryam
4
Radke, Lori90Raffin Bouchal, Shelley7Raithby, Tess74Ranger, Marie-Christine36Rapoport, Adam7Reimer, Raylene A.92Reynolds, Kathleen35Rheault, Alysson
29
Rinaldo, Emma71Robinson, Maggie4Rodin, Gary43, 73, 78Roebuck, Jaimie71Roldan Urgoiti, Gloria90Rolfe, Danielle33Rosgen, Brianna109Russell, Brooke93Rutkowski, Nicole
88
Rutledge, Rob
12
Rydall, Anne73, 78Sade, Cristina79Saikaley, Thomas89Saint-Onge, Kadia
66
Savas, Sevtap13, 39Scheidl, Jessica23Schulte, Fiona11, 23, 26, 35, 47, 7, 93Schulz-Quach, Christian
76
Schwerdtfeger, Andreas6Selby, Debbie43Shallwani, Shirin
9
Shapiro, Gilla58, 73, 76, 78Silvius, Jim93Simmonds, Charlene13Simpkins, Nia
22
Sinclair, Shane7, 8Sinnarajah, Aynharan4, 8Squires, Lauren
18
St-Cyr, Jany66, 96St-Onge, Kadia96Stern, Maya
47
Stokoe, Mehak35Stuckless, Teri13Subnis, Utkarsh94Sultan, Serge3Sung, Lillian11Sussman, Jonathan80Szewczyk, Paulina82Tabaczynski, Allyson112Tang, Patricia94Taylor-Brown, Jill49Tenkorang, Eric Y.13Thangarasa, Tharshika43Thavorn, Kednapa54, 56, 57Thibodeau, Kimberley31, 39Thomas, Roanne29, 33, 36, 9Thompson, Genevieve8Thorne, Sally60Thorson, Katherine6Thuraisingham, Nilani
89
Tomeh, Marell
40
Tong, Eryn43, 58Torrejon, Maria60Toupin-April, Karine9Tozer, Richard80Tracey, Leah89Trachtenberg, Lianne70Tran, Andrew26Tremblay, Dominique28, 59Trinh, Linda112Tromburg, Courtney26Truant, Tracy L.O.49Trudel, Geneviève88Tsimicalis, Argerie69Tulk, Joshua
48
Turcotte, Annie28, 59Tutelman, Perri
47
Tyson, Emily
65
Vanstone, Ruth
46
Vigier, Marta
6
Vora, Tushar72Wai, Alan76Wang, Chuyue89Wang, Jenney
107
Warner, Ellen46Waserman, Jessica80Wasserman, Sydney81, 89Waterman, Meghan
111
Watling, Cody Z.49Watson, Linda
93
Whitehorn, Alexis112Wilson, Beverly38Winsor, Mercy13Wolever, Ruth94Wolff, Angela60Wong, Jiahui70Wong, Rebecca73Woodard, Stephanie106, 108Woodcock, Joy70Wouterloot, Elise
110
Wright, Meaghan107Wu, Melinda71Wulf, Susan70Wurz, Amanda11, 38, 61Zhang, Karen
80
Zimmermann, Camilla73Zwicker, Victoria107

